# Estimating health care delivery system value for each US state and testing key associations

**DOI:** 10.1111/1475-6773.13676

**Published:** 2021-05-24

**Authors:** Joseph L Dieleman, Alexander S Kaldjian, Maitreyi Sahu, Carina Chen, Angela Liu, Abby Chapin, Kirstin Woody Scott, Aleksandr Aravkin, Peng Zheng, Ali Mokdad, Christopher JL Murray, Kevin Schulman, Arnold Milstein

**Affiliations:** ^1^ Institute for Health Metrics and Evaluation, Hans Rosling Center University of Washington Seattle Washington USA; ^2^ Bluesquare SA Brussels Belgium; ^3^ Department of Health Policy and Management, Bloomberg School of Public Health Johns Hopkins University Baltimore Maryland USA; ^4^ Department of Emergency Medicine University of Michigan Ann Arbor Michigan USA; ^5^ Institute for Health Metrics and Evaluation and Department of Applied Mathematics University of Washington Seattle Washington USA; ^6^ Clinical Excellence Research Center Stanford University Stanford California USA

**Keywords:** comparative health systems, health care costs, state health policies

## Abstract

**Objective:**

To estimate health care systems' value in treating major illnesses for each US state and identify system characteristics associated with value.

**Data sources:**

Annual condition‐specific death and incidence estimates for each US state from the Global Burden Disease 2019 Study and annual health care spending per person for each state from the National Health Expenditure Accounts.

**Study design:**

Using non‐linear meta‐stochastic frontier analysis, mortality incidence ratios for 136 major treatable illnesses were regressed separately on per capita health care spending and key covariates such as age, obesity, smoking, and educational attainment. State‐ and year‐specific inefficiency estimates were extracted for each health condition and combined to create a single estimate of health care delivery system value for each US state for each year, 1991–2014. The association between changes in health care value and changes in 23 key health care system characteristics and state policies was measured.

**Data collection/extraction methods:**

Not applicable.

**Principal findings:**

US state with relatively high spending per person or relatively poor health‐outcomes were shown to have low health care delivery system value. New Jersey, Maryland, Florida, Arizona, and New York attained the highest value scores in 2014 (81 [95% uncertainty interval 72‐88], 80 [72‐87], 80 [71‐86], 77 [69‐84], and 77 [66‐85], respectively), after controlling for health care spending, age, obesity, smoking, physical activity, race, and educational attainment. Greater market concentration of hospitals and of insurers were associated with worse health care value (*p*‐value ranging from <0.01 to 0.02). Higher hospital geographic density and use were also associated with worse health care value (*p*‐value ranging from 0.03 to 0.05). Enrollment in Medicare Advantage HMOs was associated with better value, as was more generous Medicaid income eligibility (*p*‐value 0.04 and 0.01).

**Conclusions:**

Substantial variation in the value of health care exists across states. Key health system characteristics such as market concentration and provider density were associated with value.

**What is known and what this study adds**

**What is known**

- Health care value has been defined as “health outcomes achieved per dollar spent.”
- Health outcomes and spending vary dramatically across US states, but there has been little rigorous research estimating health care value for each US state.
- Health system characteristics such as provider and insurance market concentration and provider density have been shown to be associated with the cost of health care.

**What this study adds**

- Relative to spending on health and exogenous factors, such as obesity rates, smoking rates, age, and education level, New Jersey, Maryland, Florida, Arizona, and New York had high value health care delivery systems in 2014.
- Rankings of health care delivery system value across US states have changed dramatically across time.
- Provider and insurance market concentration and provider density are associated with worse health care delivery system value.

## 1 INTRODUCTION

Porter and colleagues famously defined health care value as “health outcomes achieved per dollar spent.”^1^, ^2^ Within the US, health outcomes and health care spending vary dramatically. In 2014, state‐specific life‐expectancy spanned from 74.9 years (in Mississippi) to 81.2 years (in Hawaii), while health care spending per person spanned $5982 (in Utah) to $11,064 (in Alaska). This variation (and the lack of concordance between high spenders and high health achievers) suggests that health care value is likely to also vary across US states. Estimating health care value and the association between state‐specific measures of health care value and key health care system characteristics may provide transferable insights for state health policy‐making.

Despite the simplicity of Porter and colleagues' definition, many things impact health care value, including many things that are determined outside of the health sector. Previous efforts to measure health care value have generally not controlled for these non‐health care determinants of health. In addition, previous efforts have been limited to cross‐sectional analysis, have not adjusted for varying prices across the US, have focused on a limited set of health outcomes or on process measures, and in many cases have not focused on measuring value by US state.^3^, ^4^, ^5^, ^6^, ^7^, ^8^ These limitations are important because evaluations of how health system characteristics are associated with health system value should compare apples with apples, and not be confounded by factors such as age of the population, obesity and physical activity rates, education and income rates, and economy‐wide prices.

To address this gap, this study pursued three main objectives: (1) identify which US states had the highest and lowest levels of health care delivery system value after adjusting for prices and key population characteristics, (2) to assess how value rankings for each state have changed between 1991–2014, and (3) evaluate which health system characteristics and policies were associated with higher health care value. Our estimate of value focuses on health care delivery systems' ability to treat diseases, and does not directly consider disease prevention.

## 2 METHODS

This research was conducted in two stages. In the first stage, health care delivery system value (which is referred to as health care value hereafter) was estimated. This study operationalizes the “health outcomes per dollar spent” definition of health care value by assessing the relationship between 136 health condition‐specific mortality incidence (MI) ratios and price‐adjusted per‐capita personal health care spending. To compare apples with apples, we adjusted the MI ratios for population characteristics that are known to impact disease severity and therefore also MI ratios, but are largely determined outside of the health care system. This means that if two US states have similar health outcomes (measured using MI ratios) and similar health care spending levels, the state with an older, more obese, or less educated population will be considered higher value, as treating health conditions among these groups has been shown to be more clinically complex. We use price‐adjusted US dollars to reflect the purchasing power in each state, which varies dramatically across the US. By using price‐adjusted dollars (and not price‐adjustments that are specific to the health sector) the measure of health care value is measured relative to the items that could have been purchased in each US state if the money spent on health had been spent on something else.

MI ratios are calculated by taking deaths per incident cases for each year, for each US state, for the population less than 75 years. This relationship is assessed using frontier analysis. Frontier analysis is a common method used in economics to estimate the optimal output of a system given fixed inputs.^9^ For this analysis, it was assumed that optimal output was minimizing states' MI ratio for a diverse set of health conditions, given the state's per‐capita health spending, and controlling for other key population health determinants (e.g., population age structure, obesity, education). Inefficiency was defined as the gap between the modeled optimal MI ratio for a given level of health care spending and the actual MI ratio for that state, year, and health condition assessed.

This process estimated health care inefficiency for each US state for each year (1991 through 2014) for 136 health conditions, for which mortality and incidence data were available. For each US state and year, the 136 health condition‐specific inefficiency estimates were combined to generate a single estimate of health care value that could be compared across states. While statistically sophisticated and dependent on a broad set of health outcomes, spending, and risk estimates, this health care value estimate quite directly reflects “health outcomes achieved per dollar spent” on health care. That said, our estimate of health care value is based on health care delivery system's ability to prevent mortality, given an incident case. Thus, our analysis does not consider the quality of outcomes (i.e., not directly correlated with mortality) or health care spending on prevention. If a state were to spend disproportionally more on prevention, it is possible that they could have a lower estimated measure of value because relative to states with similar health care spending levels, this state was poor at treating incident cases.

In the second stage of this study, the association between health care value and state policies and health care system characteristics was estimated using linear regression. More information about both stages is found below and in the accompanying Supplement.

### 2.1 Data

Health condition‐specific, age‐specific incidence and mortality rates for 136 causes of death were obtained from the Global Burden of Disease (GBD) Study.^10^ The GBD is a systematic analysis of health outcomes that estimates mortality, morbidity, disease prevalence, and incidence for 249 health conditions globally, and for each of the 50 states and District of Columbia across the entirety of this study time period (1991–2014). We included in our study the 136 causes of death that had both death and incidence estimates available. To generate incidence and mortality rates estimates for the US, the GBD study drew information from National Center for Health Statistics National Vital Registration Statistics System, as well as over 2400 cause‐specific studies or databases. To calculate health condition‐specific MI ratios for each US state and year, the number of deaths for those less than 75 years were divided by the number of incident cases for the same population. Excluding the oldest population groups focused our analysis on ages in which the health care delivery system is most able to prevent mortality, and where there is the most variation in outcomes. For the highest age groups, the age‐specific MI ratios climb exponentially for all states, which makes evaluating state‐specific performance difficult.

Personal health care spending estimates for all 50 states and the District of Columbia were obtained from the Centers for Medicare & Medicaid Services for years 1991–2014.^11^ These estimates were inclusive of all payers (Medicare, Medicaid, private health insurance, out‐of‐pocket, and other payers and programs) and of all health goods and services including hospital services, physician and clinical services, dental services, home health care, nursing care facilities, drug, and other nondurable products, and durable medical equipment. All spending estimates were inflation and price adjusted to reflect economy‐wide state‐specific 2019 US dollars, using implicit regional price deflation estimates across states, obtained from the Bureau of Economic Analysis.^12^

Several covariates were included in our models to adjust for underlying health risk in each state. Covariates obtained from the Global Burden of Disease study included: percentage of the population over the age of 65 years old, number of cigarettes or cigarette equivalents consumed per adult aged 15 years of older, mean years of education per capita for those 15 years and older, prevalence of obesity, and mean physical activity (minutes per week, lagged 10 years).^10^ In addition, percentage of the non‐Hispanic white population was obtained from US Census Bureau data.^13^ Adjusting for these controls is non‐trivial (as shown in the Appendix) and differentiates this research from previous measures of value. We adjusted for these controls because we believe that if, for any given health condition, being older, educated, obese, physically inactive, or of a particular race systematically leads to higher MI ratios, these characteristics of the population should be considered (and the effects removed) when evaluating the health care delivery system's value.

Twenty‐three state policy indicators or health system characteristic were available to assess for this study (see Appendix for additional variable details). These indicators are related to provider and insurance market concentration; insurance coverage, enrollment, premiums, reimbursement rates, and eligibility; service utilization; and provider and service density. To measure hospital and insurance market concentration, this study used the Herfindahl‐Hirschman Index (HHI), which is a commonly used indicator of market concentration across sectors such that larger values indicate that the market is more concentrated in a single or small group of hospitals or insurers.^14^, ^15^ HHI data for were obtained from the Kaiser Family Foundation (for large group, small group, and individual insurers) and the Health care Cost and Utilization Project Hospital Market Structure files (for hospitals), and was available for all 50 states for 2011–2014 and 5 years between 1997 and 2009 respectively.^16^, ^17^ The percentage of the population covered by private and public health insurance at the time of survey for 2008–2014 was obtained from the Kaiser Family Foundation using underlying data from the American Community Survey.^16^ In addition, Medicare and Medicaid coverage levels along with a variety of enrollment variables were obtained from the Kaiser Family Foundation.^16^ Medicare variables included Medicare Advantage (local HMO, PPO or any) enrollment (2006–2014), Medicare prescription drug plan enrollment (2007–2014), and average Medicare prescription drug plan premiums (2006–2014). Medicaid variables included Medicaid income eligibility thresholds for children (2000–2014) and pregnant women (2003–2014) as a percentage of the federal poverty line (i.e., a higher threshold indicating a more generous threshold covering more people), and whether there was an increase in Medicaid rates for any provider, inpatient rates, outpatient rates, or physician rates (all available 2003–2014). Indicators of health care utilization, including hospital admissions per person, hospital inpatient days per person, and hospital outpatient visits per person were available for 1999–2014, and were obtained from the Kaiser Family Foundation.^16^ Measures of provider and service density included: numbers of hospitals per capita, hospital beds per capita, number of employed medical doctors (of any specialty) per capita, and number of employed pharmacists/pharmaceutical assistants per capita; hospitals were obtained for 1999–2014 from Kaiser Family Foundation, and the latter three variables were available for all years included in the study and obtained from GBD Study.^10^, ^16^ Some indicators such hospital concentration reflect both health care system characteristics and state policies such as the content and enforcement of state anti‐trust laws.

### 2.2 Estimating health care value

This study measured health care value by estimating a measure of inefficiency for each state, year, and health condition using frontier analysis. Inefficiency was defined as the gap between actual health outcomes relative to the optimal modeled health outcomes (known as the frontier), conditional on key covariates detailed above. Thus, states with a high MI ratio for key health conditions and high spending would be the most inefficient. In order to adjust for national trends that impact the entire country, year‐specific national means were subtracted from state‐ and year‐specific MI ratios. Meta‐stochastic frontier analysis was used for each health condition separately. In order to adjust for different levels of disease severity and drivers of health that vary across states and are determined by factors largely outside of the health sector, the frontier analysis included the following covariates: state‐ and year‐specific educational attainment per person, the fraction of the population greater than 65 years, obesity rate, the number of cigarettes sold per person, and physical activity rate from 10 years prior. Including these covariates allows us to adjust for their systematic impact on disease severity, which impacts MI ratios. See the Appendix for a summary of sensitivity analyses that details how to control variable inclusion was modified for certain health conditions and how model specification affected the estimate of health care value.

State‐ and year‐specific estimates of health care value were constructed by taking the weighted mean of the inverse of the normalized cause‐specific inefficiency estimates. Weights were calculated by computing the year‐specific proportion of total deaths attributable to each health condition such that the measure of value is most influenced by the conditions with the greatest mortality in the US. This process produced a single estimate of health care value for each state and year, with relatively more importance placed on health conditions with higher national mortality rates.

### 2.3 Estimating the association between health care value and state characteristics and policies

To test the association between health care value and health system characteristics and policies, panel linear regression using heteroscedasticity‐robust standard errors was used.^18^ Because the data on health system characteristics and policies was sparse, separate regressions were used for each health system characteristic or policy indicator. State fixed effects were included in the regression to remove time‐invariant state‐specific confounding and to measure the association between changes in health system characteristics and policies with changes in health care value.

### 2.4 Quantifying uncertainty

To quantify the impact of data uncertainty, each step of the analysis was conducted on the estimated 1000 draws of the underlying health outcome estimates produced by the GBD study. The mean of the 1000 estimates for health care value for each state and year was reported as the point estimate, and the 2.5 and 97.5 percentiles of the 1000 estimates were reported as lower‐ and upper‐bounds of the confidence intervals, respectively. The linear regression analysis was completed separately for each of the 1000 health care value estimates, and model and data uncertainty estimates were combined using simulation.

## 3 RESULTS

**FIGURE 1:**
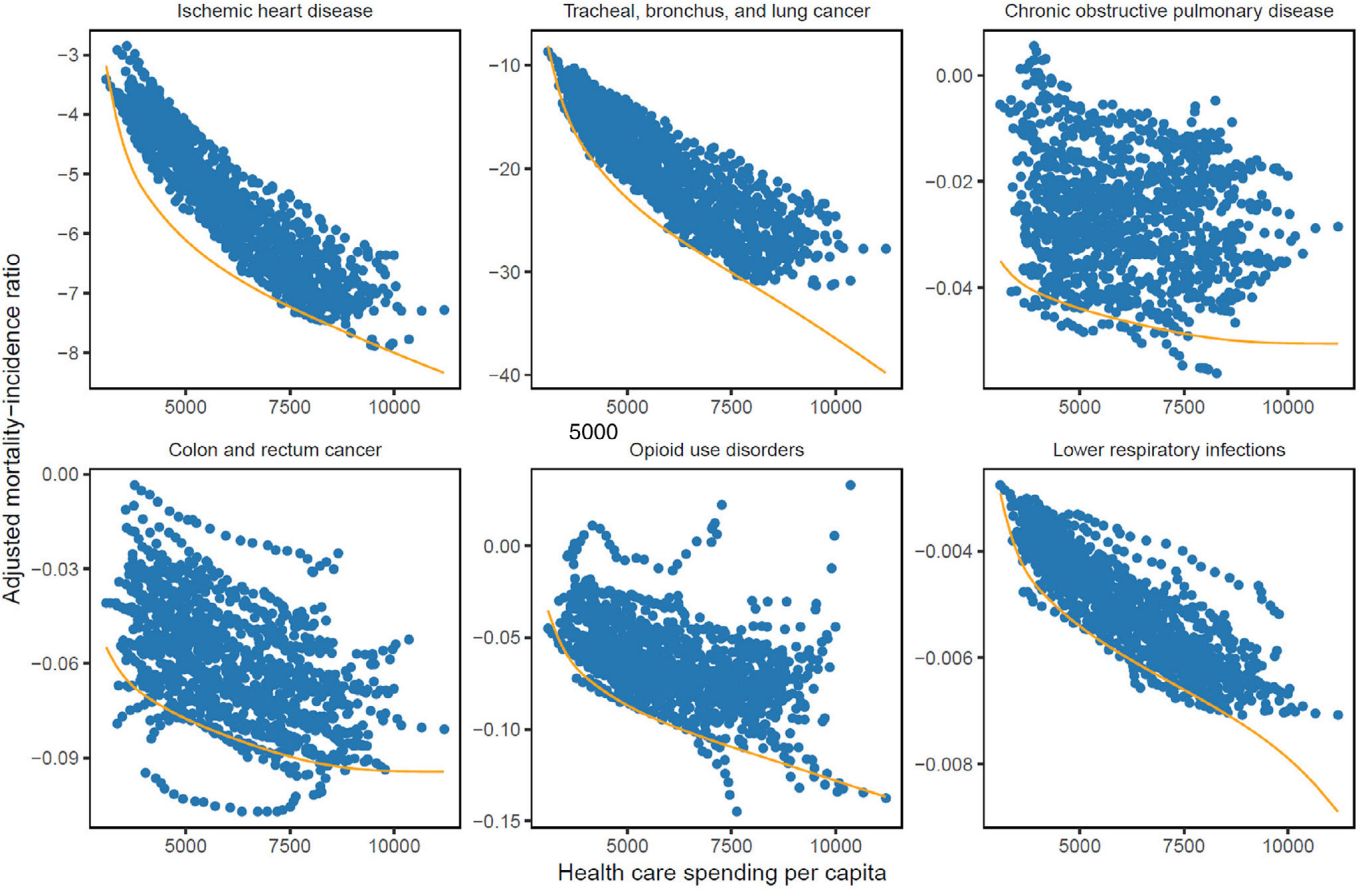
Mortality‐incidence frontiers for the six health conditions with the most mortality in the US.Notes: This figure shows the adjusted mortality‐incidence frontier for all US states, 1991–2014, for the six health conditions with the most mortality in the US in 2014. The effect of key covariates has been removed. The red line illustrates modeled frontier line which represents the best attainable outcomes, relative to each health spending level. The vertical gap between the modeled frontier line and observed data (the dots) illustrates the health condition‐, year‐, state‐specific inefficiency estimates. Panel A: Ischemic heart disease, Panel B: Tracheal, bronchus, and lung cancer, Panel C: Chronic obstructive pulmonary disease, Panel D: Colon and rectum cancer, Panel E: Opioid use disorders, and Panel F: Lower respiratory inflections [Color figure can be viewed at wileyonlinelibrary.com]

Figure 1 illustrates the frontier estimates for the six health conditions that accounted for the most deaths in the US in 2014: ischemic heart disease; tracheal, bronchus, and lung cancer; chronic obstructive pulmonary disease (COPD); colon and rectum cancer; opioid use disorders; and lower respiratory infection. These figures show that the MI ratios tend to go down as states spend more on health, holding other controlled for health determinants constant, although the relationship is stronger for conditions such as COPD than for others (e.g., ischemic heart disease). The modeled frontier line illustrates the best attainable outcomes, relative to each health spending level. The vertical gap between the modeled frontier line and observed data (the dots) illustrates the health condition‐, year‐, state‐specific inefficiency estimates.

**FIGURE 2:**
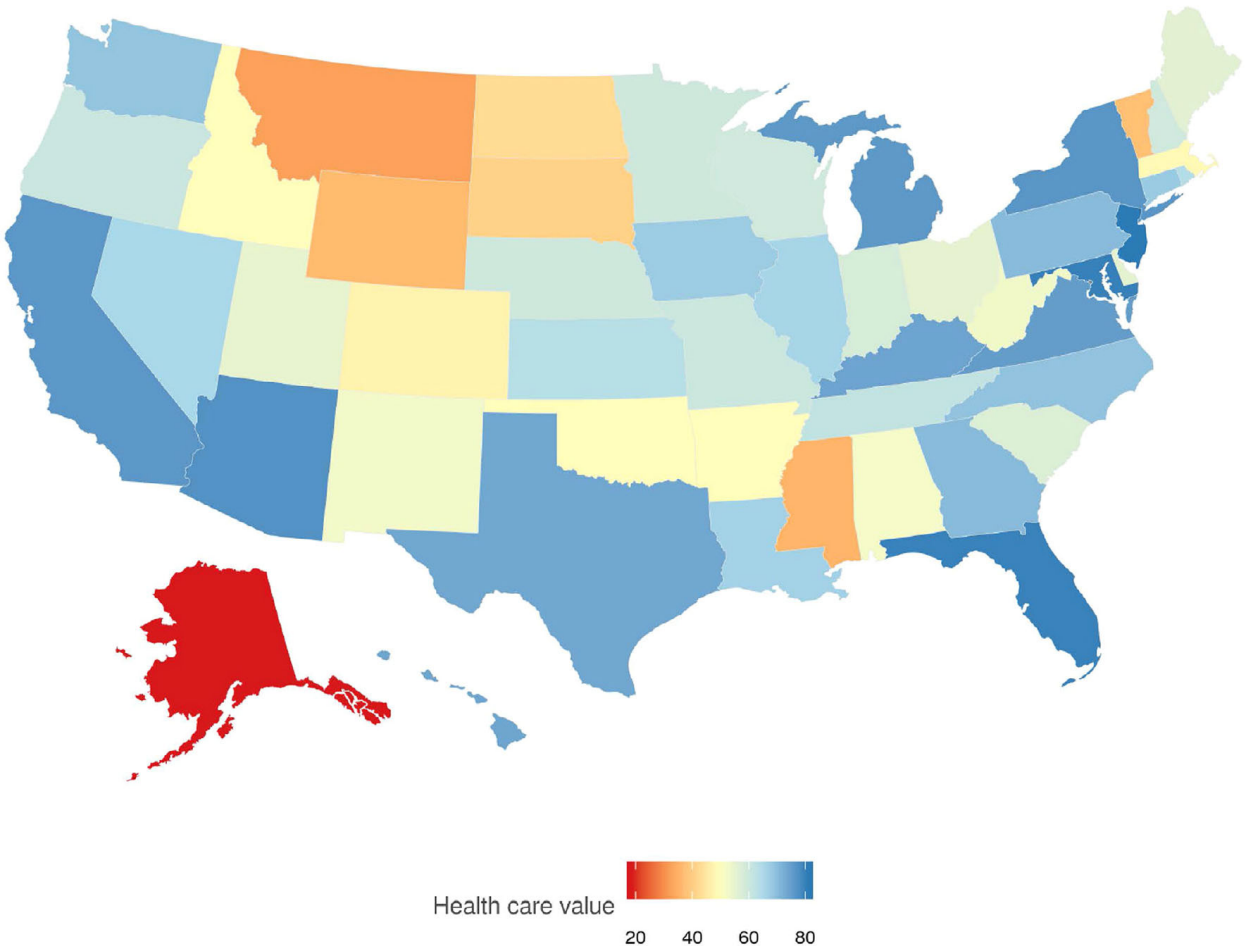
Health care value for each US state in 2014.Notes: This figure shows estimates of US health care delivery system value in 2014. 81 (New Jersey) is the highest score and identifies the state with the most health care delivery system value, given other key inputs such as age, diseases incidence, obesity and education rates. 19 (Alaska) is the lowest score and identifies the state with least health care delivery system value [Color figure can be viewed at wileyonlinelibrary.com]

**FIGURE 3:**
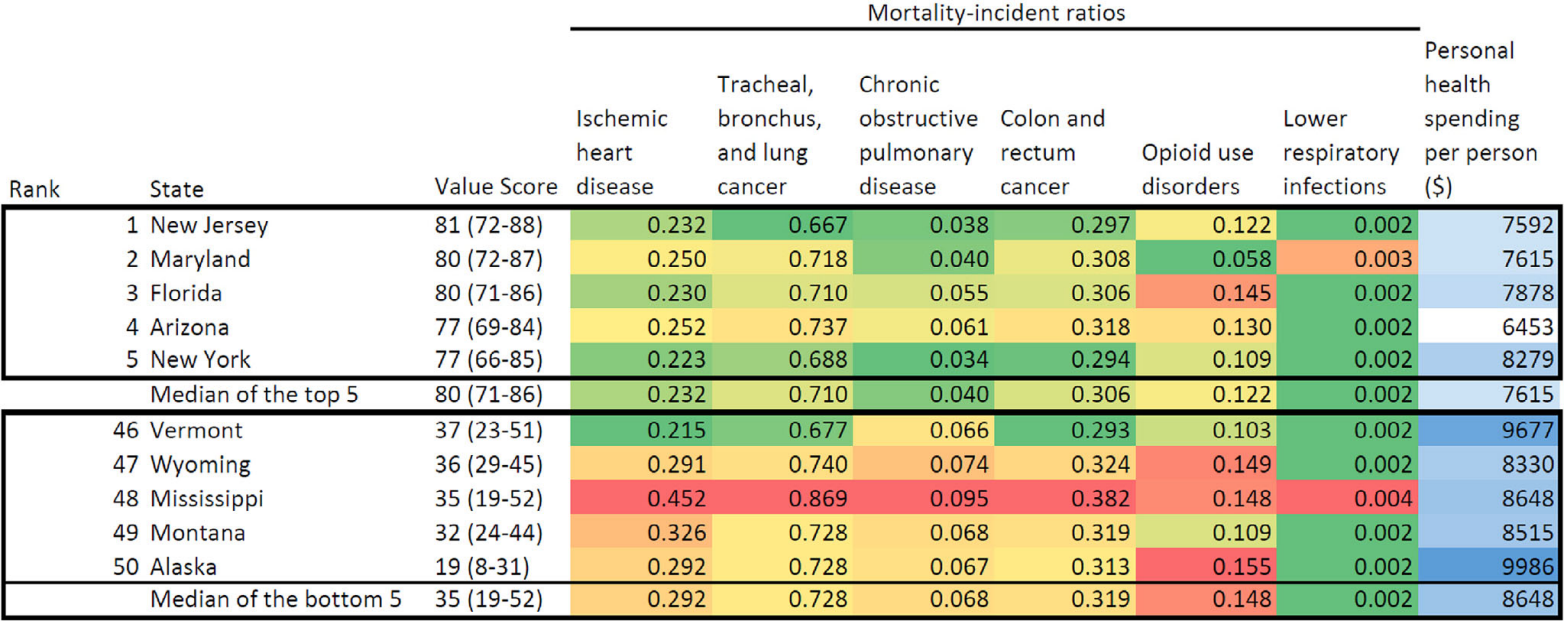
Health system value, mortality‐incidence ratio, and health care spending for the US states with the highest and lowest value, 2014.Notes: This figure shows estimates of US health care delivery system value, unadjusted mortality incidence ratios, and health care spending in 2014 [Color figure can be viewed at wileyonlinelibrary.com]

Figure 2 illustrates the health care value estimate for each state in 2014. Figure 3 shows that New Jersey, Maryland, Florida, Arizona, and New York were the states with the highest health care value in 2014. Across these five states, the median MI ratio in 2014 for ischemic heart disease; tracheal, bronchus, and lung cancer; COPD; colon and rectum cancer, opioid use disorders; and lower respiratory infections were 0.232, 0.710, 0.040, 0.306, 0.122, and 0.002, and, the median spending for these states was $7615 per person on health. Figure 3 also reports that Alaska, Montana, Mississippi, Wyoming, and Vermont were the states with the lowest health care value in 2014. Across these five states, the median MI ratio in 2014 for ischemic heart disease; tracheal, bronchus, and lung cancer; COPD; colon and rectum cancer, opioid use disorders; and lower respiratory infections were 0.292, 0.728, 0.068, 0.319, 0.148, and 0.002, and, the median health spending for these states was $8648 per person on health. Not all substantially rural states ranked poorly; Iowa ranked favorably. States with the highest value score, in general, had smaller MI ratios and less health care spending, while states with the lowest value scores, in general, had larger MI ratios and more spending.

**FIGURE 4:**
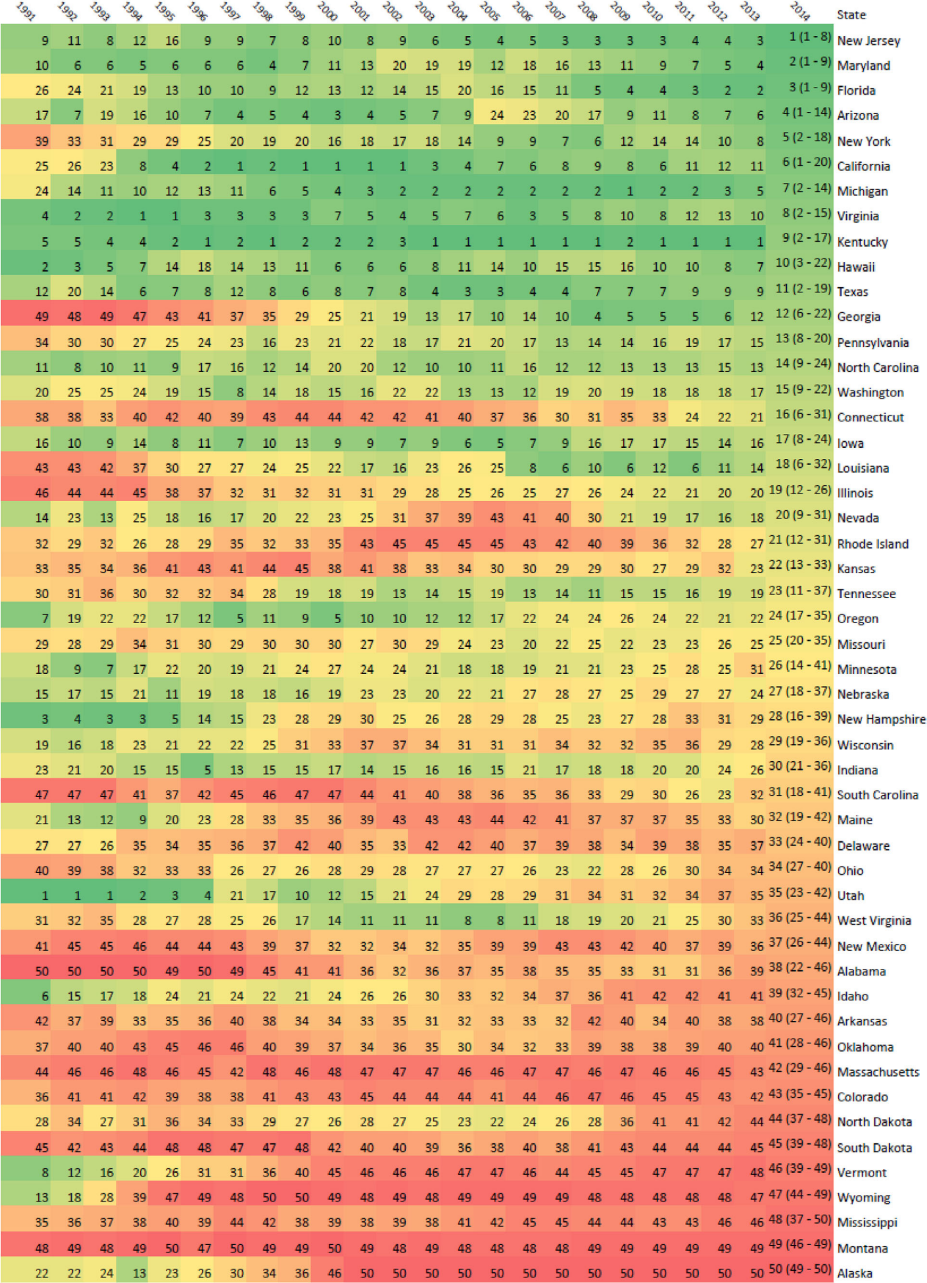
Health care value relative ranking across time, 1991 through 2014.Notes: Relative ranking of health care delivery system value for each US state, for 1991 through 2014 [Color figure can be viewed at wileyonlinelibrary.com]

Figure 4 illustrates the rank of each states value score from 1991 to 2014. In absolute terms, Georgia and New York increased the most from the rank of 49 to 12, and 39 to 5 over 23 years between 1991 and 2014, respectively. Vermont and Utah decreased the most from rank of 8 to 46, and 1 to 35 for 1991 to 2014, respectively.

**FIGURE 5:**
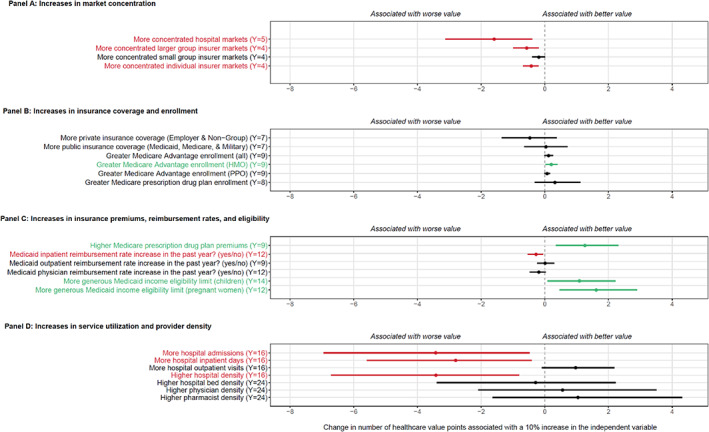
The association between health care value and health system characteristics and policies.Notes: Estimated coefficients are each from a separate bivariate, log‐linear regression, with the 95% confidence interval shown, representing the difference in health care value associated with a 10% increase in the health care system characteristic or policy. US state fixed effects were also included in order to estimate the association between changes in health system characteristics and policies with changes in health care value, for each US state. Number of years of data availability are listed for each variable. Green font indicates characteristics positively associated with value and statistically significant (alpha = 0.05), while red font indicates characteristics negatively associated with value and statistically significant [Color figure can be viewed at wileyonlinelibrary.com]

Figure 5 illustrates the estimated regression coefficients assessing the relationship between state health care value ratings and system characteristics and policies. Panel A shows that increases in health system market concentration (hospital, large group insurers, and individual insurers) are significantly associated with reductions in health care value (*p*‐values = 0.02, 0.01, and < 0.01, respectively). A 10% increase in hospital market concentration was associated with a 1.59 (95% CI, 0.39‐3.12) point reduction in health care value. Panel B shows that private insurance coverage was associated with a reduction in value, while public insurance was associated with increases in value, although neither was statistically significant (*p*‐value 0.29 and 0.92, respectively). Medicare Advantage enrollment (HMO) was positively associated with health care value (*p*‐value 0.04) while Medicare Advantage enrollment (PPO and all types) were found to have a positive but not statistically significant relationship with health care value (*p*‐value 0.09 and 0.15, respectively). Panel C shows that increases in Medicare prescription drug plan premiums and increases in Medicaid income eligibility were significantly associated with increases in value, while increases in Medicaid inpatient reimbursement rates were significantly associated with reductions in value (*p*‐value 0.02). A 10% increase in Medicaid income eligibility for children, for example, was associated with a 1.08 (95% CI, 0.09‐2.22) point increase in health care value. Panel D shows that the use of inpatient services (admissions and bed‐days) was significantly associated with reductions in health care value (*p*‐value 0.05 and 0.04, respectively). A 10% increase in hospital admissions, for example, was associated with a 3.43 (95% CI, 0.48‐6.95) point reduction in health care value. Outpatient services were positively but not statistically significantly associated with increases in health care value (*p*‐value 0.10). Finally, for health care capacity proxies, there was no association between the number of hospital beds or provider density with health care value; however, hospital density was negatively associated with health care value (*p*‐value 0.03).

## 4 DISCUSSION

In summary, this study generated novel estimates of health care value for each US state in order to test the association of health care system characteristics and state policies with health care value. These health care delivery system value estimates focus on the health systems ability to treat a broad set of conditions, and controlled for key drivers of health that are generally determined outside of the health sector, such as the age of the population, obesity and physical activity rates, education levels, and economy‐wide prices. The states that attained the highest health care value by 2014 were New Jersey, Maryland, Florida, Arizona, and New York (i.e., these states had low mortality‐incidence ratios relative to their price‐adjusted health care spending, age, obesity, smoking, educational attainment, and other non‐health care determinants). Between 1991 and 2014, Georgia, New York, and Florida witnessed the greatest absolute increases in health care value. The states rated with the lowest health care value levels in 2014 were Alaska, Montana, Mississippi, Wyoming, and Vermont. Four conclusions could be drawn from the policy analysis: (i) greater market concentration of hospitals and of insurers was associated with worse health care value, (ii) higher hospital geographic density and use were also associated with worse health care value, and (iii) enrollment in Medicare Advantage HMOs and (iv) generous Medicaid income eligibility were associated with better value.

A number of state‐level rankings of health care value exist, including the Commonwealth Fund's State Scorecard on Health System Performance, Altarum's Health Care Affordability Scorecard, and several other non‐peer reviewed rankings.^3^, ^4^, ^5^, ^6^, ^7^, ^8^ A detailed comparison between the findings from this study and these prior rankings are described and shown in section 6 of the Appendix. State rankings from this study differ from state rankings from prior studies due to three key methodological differences. First, our study assessed treatment, and not prevention. States that invest a great deal in prevention may be inadvertently penalized in our analysis, relative to other studies.

Second, this study controls for population characteristics that are generally outside of the control of the health care system. This approach offsets advantages enjoyed by health care systems in US states with younger and healthier and populations that typically enjoy less severe cases and better response to treatment. For example, states such as Colorado or Utah attained lower value rankings (ranking of 43 and 35, respectively) in our study than in prior studies that did not adjust for incidence of diseases, age, and other non‐health care determinants of mortality. On the contrary, states such as Florida and Kentucky achieved higher health care value rankings (third and ninth), as they spent relatively low amounts on health care and have disproportionally older populations with greater exposure to mortality risks such as obesity or tobacco use. As shown in Section 6 of the supplement, the health care value rankings from this study align more with other measures of value when the estimates from this study were not adjusted for these factors. This study shows that accounting for key population characteristics such as age, education, and obesity rates, which are known to influence health outcomes but are largely determined outside of the health care delivery system, has a meaningful impact on the estimation of health care value.

A third distinction between this measure of health care value and other existing estimates is that this measure is based on both health outcomes and health spending levels. Many previous measures of health care value focus on health outcomes *or* spending,^3^, ^5^, ^6^, ^8^ or focus on the health care process measures, but not true health outcomes.^4^, ^6^ Some states that are known for performing highly in terms of health outcomes, such as Massachusetts and Vermont, do worse in this estimate of health care value because they spend disproportionally more on health (ranked 42nd and 46th, respectively, and also because they have relatively more educated populations with fewer risk factors). Conversely, Georgia, which had health outcomes commensurate with the national mean, but spends much less, had a relatively high ranking (12th).

An important component of this analysis is that it generated annual estimates of health care value for substantial time span (1991 through 2014). Having a 24‐year panel of estimates enabled the secondary analyses that measured the association between changes in health care value and changes in state health system characteristics and policies. This research highlights that state health policy and health system characteristics matter and were associated with health care value.

This study shows that market concentration was negatively associated with a state's health care value rating. Hospital and insurer markets have become increasingly concentrated since the 1990s.^14^, ^19^, ^20^ Prior research has shown that hospital mergers and acquisitions are associated with substantial increases in prices for health services charged to private insurers, while hospitals in competitive markets focus more on reducing costs.^19^, ^21^, ^22^, ^23^ Furthermore, hospital consolidation has been associated with reduced patient satisfaction of care.^24^ Adding a single rival hospital has been shown to increase survival rates from emergency heart attacks by nearly 10%.^25^ Market reforms introduced by the National Health Service in England to encourage patient choice and create quality competition between hospitals have been associated with reduced mortality.^26^ In addition, greater competition among insurers has been shown to be strongly associated with reduced premiums; adding (or eliminating) a single insurer to a marketplace can lead to decreases (or increases) in premiums of 4.5‐16.6%.^23^, ^27^, ^28^ This research provides further support for state policies which encourage greater competition among hospitals and insurers, such as restricting and severely penalizing anticompetitive behaviors.^14^, ^29^, ^30^

This study also highlights that some aspects of Medicaid and Medicare were associated with better value. More generous Medicaid eligibility criteria for pregnant women and children was associated with higher health care value, and increases in Medicaid reimbursement rates are negatively associated with value. The percentage of Medicare eligible enrolled in Medicare Advantage, in particular Medicare Advantage HMOs, was significantly associated with health care value. In contrast to traditional Medicare fee‐for‐service, Medicare Advantage plans can improve value via greater selectivity of physicians and hospitals. Efforts to expand insurance coverage can be combined with other strategies to ensure that these efforts to improve the value as well as access.^29^

These results also highlight the disparate relationship between health care spending and health outcomes, even after controlling for key drivers largely determined outside of the health sector. For some health conditions, such as opioid use disorders, there was little relationship between spending and reduction in mortality per incident case, while other conditions such as COPD display a very strong relationship.

This research has several limitations. First, there is no single, agreed upon method for measuring health care value. This research focuses on measuring the ability for the health care system within each state to drive down MI ratios, relative to the amount spent on health and a standardized set of inputs. This intuitively captures the idea of value well, but alternative measurement methods can and have been conceptualized. Using MI ratios as a key outcome to measure spending against means that our research does not address quality of care (i.e., not correlated with mortality) or prevention. A well‐functioning health system should aim to prevent illness in the first place ‐ by driving down exposure to adverse risk factors that increase incidence or severity. The estimates from this study did not “reward” health systems that invest disproportionally in preventing illness and driving down exposure to these risks. Moreover, by focusing on mortality rather than morbidity or underlying patient satisfaction, we focused on quality only as it related to preventing mortality. This choice means that health systems' efforts to treat high‐morbidity but low‐mortality health conditions or to improve patient satisfaction will be disproportionally undervalued in these rankings. Future research should be expanded to account for this limitation. Second, the empirical model on which our estimates are derived can be specified in a diverse set of ways, including a different set of controls, relying on a subset of health conditions pre‐determined to be amenable to high quality health care,^31^, ^32^ and relying on morbidity data rather than mortality data. A diverse set of sensitivity analyses are included in the supplement and show that the qualitative findings of this study are robust. Third, the analyses assessing the association of health care value and health system characteristics and policies measured correlations of changes across time, and should not be considered causal. Additional research is needed to assess the causal connection between these key variables and health care value. Fourth, frontier methods derive an optimal production frontier relative to the best observed performers. This analysis considered each of the 50 US states. While a frontier model based on best‐performing US states is valuable because it identifies the best cases among peers, this is a relative ranking and cannot make any claims that the absolute level of value ‐ even among the top performer ‐ is optimal. It is quite possible that no US state achieves the optimal health outcomes. Fifth, this analysis only extended to 2014 as this was the last available year of health spending data from the Center for Medicare and Medicaid services. As more recent data become available, this research should be updated, especially in light of dynamic changes to health care delivery due to both health reform and COVID‐19. Finally, this research does not identify the point in the delivery cascade where value gains can be made. For example, one aspect of efficiently treating a disease is a timely and accurate diagnosis. This research does not distinguish between diagnosis and treatment ‐ which are both important for value. States which have poor value can make gains in either improving either diagnosis or treatment. Further research is needed to identify the point at which the greatest gains can be achieved.

Diverse innovations in health policy intended to improve health care value occurred across the 50 US states over this time series. In order to track the health impact of these state interventions, robust measures of health are needed. This study provides novel methods and estimates of health care delivery system value, using a broad set of health outcomes (136 health conditions) and a time series spanning 24 years, controlling for disease incidence in order to focus exclusively on the value of health care treatment (which makes up the majority of health care spending), adjusting for price variation, using non‐linear methods that more precisely track the complex relationship between health outcomes and spending, and controlling for other key drivers in health outcomes such as the age of the population, obesity, smoking, and education rates that are largely determined outside of the health sector. Controlling for key drivers of health that are not directly controlled by the health care system was especially important since population characteristics vary dramatically by state. Not controlling for these important determinants of health would lead to exaggerated value scores for the states with the healthiest populations, regardless of the role health care played in shaping those health outcomes. After controlling for these factors, substantial variation in the value of health care exists across states, with states changing ranking a great deal over the last several decades. Key health system characteristics such as market concentration and provider density are negatively associated with value and could be the focus of ongoing efforts to improve health system value. Tracking best‐performing states over time and identifying associations with state policies and health care system characteristics may provide transferable insights for less well‐performing states to consider.

## ACKNOWLEDGMENTS

All authors received support from the Stanford Clinical Excellence Research Center and have no financial conflicts of interest to report.

## Supporting information


**Appendix S1**: Supporting Information
