# Supplementary material for: Estimating health care delivery system value for each US state and testing key associations
Source: Health Serv Res. 2021 May 24;57(3):557–67. doi: 10.1111/1475-6773.13676 (PMC9108083; doi:10.1111/1475-6773.13676)
Supplement: Supplementary file 1 — Appendix S1: Supporting Information [file HESR-57-557-s001.docx]

# Supplement: Estimating healthcare delivery system value for each US state and testing key associations

**Version:** September 14, 2020

Table of Contents

[Section 1. Definitions and sources for all data 3](bookmark://_Toc40646358#_Toc40646358)

[Section 2. Descriptive Statistics for Key Variables 7](bookmark://_Toc40646359#_Toc40646359)

[Section 3. Spending and covariates over time, 1991-2014 14](bookmark://_Toc40646360#_Toc40646360)

[Section 4. Frontier Analysis Methodology 22](bookmark://_Toc40646361#_Toc40646361)

[Section 5. Primary Frontier Analysis - Estimated Covariate Coefficients 28](bookmark://_Toc40646362#_Toc40646362)

[Section 6. Comparisons with other estimates of value 39](bookmark://_Toc40646363#_Toc40646363)

[Section 7. Inefficiency Scores with Alternate Specifications 44](bookmark://_Toc40646364#_Toc40646364)

[Section 8. Sensitivity Analysis for Policy Variable Modeling 51](bookmark://_Toc40646365#_Toc40646365)

[References 61](bookmark://_Toc40646366#_Toc40646366)

# Section 1. Definitions and sources for all data

The following section includes a summary table (**Table 1**) of the variables included in this analysis, including a definition and information regarding the source from which each variable was obtained.

**Table 1.** Summary of Key Variables Included in Analysis

| **Variable** | **Full Definition** | **Source** |
| --- | --- | --- |
| **Spending** | | |
| Personal health care spending (unadjusted) | Personal healthcare spending estimates by state, according to the state of residence. These estimates were inclusive of all payers (Medicare, Medicaid, private health insurance, out-of-pocket, and other payers and programs) and of all health goods and services including hospital services, physician and clinical services, dental services, home healthcare, nursing care facilities, drug and other nondurable products, and durable medical equipment. | Centers for Medicare & Medicaid Services^1^ |
| Regional price-adjusted personal health spending | Personal healthcare spending estimates by state, according to the state of residence. These estimates were inclusive of all payers (Medicare, Medicaid, private health insurance, out-of-pocket, and other payers and programs) and of all health goods and services including hospital services, physician and clinical services, dental services, home healthcare, nursing care facilities, drug and other nondurable products, and durable medical equipment.  These values were inflation and price adjusted to reflect economy-wide state-specific 2019 US dollars, using implicit regional price deflation. | Centers for Medicare & Medicaid Services^1^, Bureau of Economic Analysis^2^ |
| Physician salary-adjusted personal health spending | This estimate uses the same measure of personal healthcare spending as above, inflation adjusted to 2019 US dollars, but the state specific adjustment is generated using average physician salaries for the state (i.e. spending is multiplied by a scalar of the average annual wage for physicians for the state divided by the mean annual wage for physicians nationally). | Centers for Medicare & Medicaid Services^1^, US Bureau of Labor Statistics^3^ |

| **Cause-specific deaths and incidence** | | |
| --- | --- | --- |
| Cause-specific deaths | Estimates of cause- and age- specific death counts for people under the age of 75 in each US state and the District of Columbia, 1991 to 2014, for each of the 136 causes studied | Global Burden of Disease Study^4^ |
| Cause specific incidence | Estimates of cause- and age- specific incidence rates for people under the age of 75 in each US state and the District of Columbia, 1991 to 2014, for each of the 136 causes studied | Global Burden of Disease Study^4^ |
| **Covariates** | | |
| Population over 65 (proportion) | Proportion of population over the age of 65. | Global Burden of Disease Study^4^ |
| Tobacco use (cigarettes per capita) | Number of cigarettes or cigarette equivalents consumed per adult aged 15 years or older per year. | Global Burden of Disease Study^4^ |
| Education (years per capita) | Average years per capita of education (for 15+ population only), aggregated by age and sex. | Global Burden of Disease Study^4^ |
| Prevalence of obesity | Proportion of the population with Body Mass Index ≥30 kg/m^2^. | Global Burden of Disease Study^4^ |
| Total Physical Activity, 10 year lag | Average per capita physical activity per week (in metabolic-equivalent minutes), age-specific. | Global Burden of Disease Study^4^ |
| Fraction of the population white, non-Hispanic | Proportion of the population identifying as white and non-Hispanic. | US Census Bureau^5^ |
| Regional-adjusted LDI (I$ per capita) | Lag distributed income per capita (I$): gross domestic product per capita that has been smoothed over the preceding 10 years. These values were price adjusted using implicit regional price deflation. | Global Burden of Disease Study^4^, Bureau of Economic Analysis^2^ |
| **Policy Variables*** | | |
| Hospital market concentration | Herfindahl-Hirschman Index** is calculated by squaring the market share for each firm (up to 50 firms) and then summing the squares; this was calculated as an average across counties using a variable radius capturing 90% of the hospital’s discharges. | Healthcare Cost and Utilization Project^6^ |
| Large group insurer market concentration | Herfindahl-Hirschman Index is calculated by squaring the market share for each firm (up to 50 firms) and then summing the squares. | Kaiser Family Foundation^7^ |
| Small group insurer market concentration | Herfindahl-Hirschman Index is calculated by squaring the market share for each firm (up to 50 firms) and then summing the squares. | Kaiser Family Foundation^7^ |
| Individual insurer market concentration | Herfindahl-Hirschman Index is calculated by squaring the market share for each firm (up to 50 firms) and then summing the squares. | Kaiser Family Foundation^7^ |
| Private insurance coverage (Employer & Non-Group) | Percent of the population covered by employer and non-group health insurance at the time of the survey. Employer insurance includes those covered through a current or former employer or union, either as policyholder or as dependent. Non-group insurance includes those covered by a policy purchased directly from an insurance company, either as policyholder or as dependent. | Kaiser Family Foundation^7^ |
| Public insurance coverage (Medicaid, Medicare, & Military) | Percent of the population covered by Medicaid, Medicare, and military health insurance at the time of the survey. Medicaid Includes those covered by Medicaid, Medical Assistance, Children's Health Insurance Plan (CHIP) or any kind of government-assistance plan for those with low incomes or a disability, as well as those who have both Medicaid and another type of coverage, such as dual eligibles who are also covered by Medicare.  Medicare includes those covered by Medicare, Medicare Advantage, and those who have Medicare and another type of non-Medicaid coverage where Medicare appears to be the primary payer. Excludes seniors who also report employer-sponsored coverage and full-time work, and those covered by Medicare and Medicaid (dual eligibles).  Military includes those covered under the military or Veterans Administration . | Kaiser Family Foundation^7^ |
| Medicare Advantage enrollment (all) | Proportion of the above 65 year old population enrolled in Medicare Advantage (any plan type). | Kaiser Family Foundation^7^ |
| Medicare Advantage enrollment (HMO) | Proportion of the above 65 year old population enrolled in Medicare Advantage (HMO plan type). | Kaiser Family Foundation^7^ |
| Medicare Advantage enrollment (PPO) | Proportion of the above 65 year old population enrolled in Medicare Advantage (PPO plan type). | Kaiser Family Foundation^7^ |
| Medicare prescription drug plan enrollment | Stand-alone Prescription Drug Plan enrollees as a percent of total Medicare population. | Kaiser Family Foundation^7^ |
| Medicare prescription drug plan premiums | Average premiums for Prescription Drug Plans. These values were price adjusted using implicit regional price deflation. | Kaiser Family Foundation^7^ |
| Medicaid inpatient reimbursement rate increase | Was there an increase in Medicaid inpatient reimbursement rate in the past year? (Yes/No) | Kaiser Family Foundation^7^ |
| Medicaid outpatient reimbursement rate increase | Was there an increase in Medicaid outpatient reimbursement rate in the past year? (Yes/No) | Kaiser Family Foundation^7^ |
| Medicaid physician reimbursement rate increase | Was there an increase in Medicaid physician reimbursement rate in the past year? (Yes/No) | Kaiser Family Foundation^7^ |
| Medicaid income eligibility limit (children) | Highest income eligible for Medicaid and CHIP for children, as percentage of the federal poverty line | Kaiser Family Foundation^7^ |
| Medicaid income eligibility limit (pregnant women) | Highest income eligible for Medicaid and CHIP for pregnant women, as percentage of the federal poverty line | Kaiser Family Foundation^7^ |
| Hospital admissions | Hospital admissions per 1000 population for community hospitals, which represent 85% of all hospitals. | Kaiser Family Foundation^7^ |
| Inpatient days | Hospital inpatient days per 1000 population for community hospitals, which represent 85% of all hospitals. | Kaiser Family Foundation^7^ |
| Outpatient visits | Hospital outpatient visits per 1000 population for community hospitals, which represent 85% of all hospitals. | Kaiser Family Foundation^7^ |
| Hospital density | Number of community hospitals per 100,000 population (community hospitals represent 85% of all hospitals). | Kaiser Family Foundation^7^ |
| Hospital bed density | Hospital beds per 1000 population. | Global Burden of Disease Study^7^ |
| Physician density | Number of employed medical doctors per 10,000 population. | Global Burden of Disease Study^7^ |
| Pharmacist density | Number of employed pharmacists and pharmaceutical assistants per 10,000 population. | Global Burden of Disease Study^7^ |

*All policy variables were in logarithmic form in the final regression analysis.

** As described in the Kaiser Family Foundation State Health Facts, “Herfindahl Hirschman Index is a measure of how evenly market share is distributed across insurers in the market. HHI values range from 0 to 10,000, with an HHI closer to zero indicating a more competitive market and closer to 10,000 indicating a less competitive market. An HHI index below 1,000 generally indicates a highly competitive market; an HHI between 1,000 and 1,500 indicates an unconcentrated market; a score between 1,500 and 2,500 indicates moderate concentration; and a value above 2,500 indicates a highly concentrated (uncompetitive) market.”^7^

# Section 2. Descriptive Statistics for Key Variables

The following section includes the descriptive statistics for key variables, including spending (unadjusted and adjusted for state-specific prices) (**Table 2.1**), a listing of all covariates included in the primary analysis (**Table 2.2**), a summary of all included policy variables and health system characteristics (**Table 2.3**), and the mean mortality incidence (MI) ratio for all 136 health causes included in this analysis (**Table 2.4**).

**Table 2.1.** Descriptive Statistics for Key Spending Variables

| Variable | Mean | Standard Deviation | Number of State-Years Available | Number of years available |
| --- | --- | --- | --- | --- |
| Spending (unadjusted) | 6781 | 1843 | 1224 | 24 |
| Regional adjusted spending | 6223 | 1641 | 1224 | 24 |
| Physician salary-adjusted spending | 6729 | 1815 | 1224 | 24 |

**Table 2.2.** Descriptive Statistics for Covariates Included in Analysis

| Variable | Mean | Standard Deviation | Number of State-Years Available | Number of years available |
| --- | --- | --- | --- | --- |
| Population Over 65 (proportion) | 0.12 | 0.02 | 1224 | 24 |
| Tobacco (cigarettes per capita) | 941 | 286 | 1224 | 24 |
| Education (years per capita) aggregated by age (15+) and sex | 13.9 | 0.4 | 1224 | 24 |
| Prevalence of obesity | 0.21 | 0.05 | 1224 | 24 |
| Total Physical Activity (MET-min/week), Age-specific, 10 year lag | 5298 | 378 | 1224 | 24 |
| Population Density (over 1000 ppl/sq km, proportion) | 0.56 | 0.19 | 1224 | 24 |
| regional-adjusted LDI (I$ per capita) | 44337 | 7194 | 1224 | 24 |
| Fraction of the population - white, non-Hispanic | 0.75 | 0.16 | 1224 | 24 |
| Fraction of the population - black, non-Hispanic | 0.11 | 0.12 | 1224 | 24 |
| Fraction of the population - native, non-Hispanic | 0.016 | 0.030 | 1224 | 24 |
| Fraction of the population - asian, non-Hispanic | 0.038 | 0.085 | 1224 | 24 |
| Fraction of the population - black, Hispanic | 0.005 | 0.005 | 1224 | 24 |
| Fraction of the population - native, Hispanic | 0.003 | 0.003 | 1224 | 24 |
| Fraction of the population - asian, Hispanic | 0.002 | 0.006 | 1224 | 24 |
| Fraction of the population - white, Hispanic | 0.076 | 0.087 | 1224 | 24 |

**Table 2.3.** Descriptive Statistics of Policy Variables Included in Analysis

| Variable | Mean | Standard Deviation | Number of State-Years Available | Number of years available |
| --- | --- | --- | --- | --- |
| Hospital admissions | 116 | 26 | 816 | 16 |
| Hospital bed density | 3.9 | 1.3 | 1224 | 24 |
| Medicaid income eligibility limit (children) | 2.28 | 0.51 | 712 | 14 |
| Hospital market concentration | 0.442 | 0.122 | 141 | 5 |
| Individual insurer market concentration | 4090 | 1792 | 204 | 4 |
| Large group insurer market concentration | 4346 | 1891 | 203 | 4 |
| Small group insurer market concentration | 3859 | 1671 | 203 | 4 |
| Hospital density | 2.28 | 1.35 | 816 | 16 |
| Inpatient days | 685 | 230 | 816 | 16 |
| Private insurance coverage (Employer & Non-Group) | 0.562 | 0.060 | 357 | 7 |
| Public insurance coverage (Medicaid, Medicare, & Military) | 0.305 | 0.049 | 357 | 7 |
| Outpatient visits | 2236 | 778 | 816 | 16 |
| Medicare Advantage enrollment (all) | 0.195 | 0.128 | 459 | 9 |
| Medicare Advantage enrollment (HMO) | 0.123 | 0.107 | 443 | 9 |
| Medicare Advantage enrollment (PPO) | 0.050 | 0.063 | 459 | 9 |
| Medicare prescription drug plan enrollment | 0.429 | 0.090 | 408 | 8 |
| Pharmacist density | 6.14 | 1.43 | 1224 | 24 |
| Physician density | 20.3 | 7.5 | 1224 | 24 |
| Medicaid income eligibility limit (pregnant women) | 1.91 | 0.41 | 612 | 12 |
| Medicare prescription drug plan premiums | 50.4 | 7.9 | 459 | 9 |
| Medicaid inpatient reimbursement rate increase (yes/no) | 1.452 | 0.498 | 611 | 12 |
| Medicaid outpatient reimbursement rate increase (yes/no) | 1.338 | 0.473 | 453 | 9 |
| Medicaid physician reimbursement rate increase (yes/no) | 1.278 | 0.448 | 609 | 12 |

**Table 2.4.** Mean Mortality Incidence (MI) Ratio Table

| Health Condition | Mean | Standard Deviation | Number of State-Years Available | Number of years available |
| --- | --- | --- | --- | --- |
| HIV/AIDS resulting in other diseases | 0.4502 | 0.3539 | 1224 | 24 |
| Diarrheal diseases | <0.0001 | <0.0001 | 1224 | 24 |
| Lower respiratory infections | 0.0021 | 0.0004 | 1224 | 24 |
| Upper respiratory infections | <0.0001 | <0.0001 | 1224 | 24 |
| Otitis media | <0.0001 | <0.0001 | 1224 | 24 |
| Pneumococcal meningitis | 0.0478 | 0.0172 | 1224 | 24 |
| Other meningitis | 0.0179 | 0.0065 | 1224 | 24 |
| Encephalitis | 0.0389 | 0.0117 | 1224 | 24 |
| Varicella and herpes zoster | <0.0001 | <0.0001 | 1224 | 24 |
| Maternal hemorrhage | 0.0003 | 0.0002 | 1224 | 24 |
| Maternal sepsis and other maternal infections | 0.0001 | <0.0001 | 1224 | 24 |
| Maternal hypertensive disorders | 0.0003 | 0.0002 | 1224 | 24 |
| Maternal obstructed labor and uterine rupture | 0.0001 | 0.0001 | 1224 | 24 |
| Neonatal preterm birth | 0.0171 | 0.0060 | 1224 | 24 |
| Neonatal encephalopathy due to birth asphyxia and trauma | 0.1754 | 0.1046 | 1224 | 24 |
| Neonatal sepsis and other neonatal infections | 0.0357 | 0.0171 | 1224 | 24 |
| Protein-energy malnutrition | 0.0006 | 0.0002 | 1224 | 24 |
| Syphilis | 0.0006 | 0.0002 | 1224 | 24 |
| Chlamydial infection | <0.0001 | <0.0001 | 1224 | 24 |
| Gonococcal infection | <0.0001 | <0.0001 | 1224 | 24 |
| Acute hepatitis A | 0.0003 | 0.0002 | 1224 | 24 |
| Acute hepatitis B | 0.0011 | 0.0007 | 1224 | 24 |
| Acute hepatitis C | 0.0005 | 0.0003 | 1224 | 24 |
| Acute hepatitis E | 0.0008 | 0.0005 | 1224 | 24 |
| Esophageal cancer | 0.8822 | 0.0534 | 1224 | 24 |
| Stomach cancer | 0.5364 | 0.0732 | 1224 | 24 |
| Liver cancer due to hepatitis C | 0.7746 | 0.0922 | 1224 | 24 |
| Larynx cancer | 0.2886 | 0.0385 | 1224 | 24 |
| Tracheal, bronchus, and lung cancer | 0.7616 | 0.0611 | 1224 | 24 |
| Breast cancer | 0.1759 | 0.0197 | 1224 | 24 |
| Cervical cancer | 0.2675 | 0.0221 | 1224 | 24 |
| Uterine cancer | 0.0880 | 0.0132 | 1224 | 24 |
| Prostate cancer | 0.0740 | 0.0180 | 1224 | 24 |
| Colon and rectum cancer | 0.3372 | 0.0314 | 1224 | 24 |
| Lip and oral cavity cancer | 0.2113 | 0.0158 | 1224 | 24 |
| Other pharynx cancer | 0.2414 | 0.0253 | 1224 | 24 |
| Gallbladder and biliary tract cancer | 0.4200 | 0.0583 | 1224 | 24 |
| Pancreatic cancer | 0.8731 | 0.0597 | 1224 | 24 |
| Malignant skin melanoma | 0.1196 | 0.0232 | 1224 | 24 |
| Ovarian cancer | 0.5384 | 0.0248 | 1224 | 24 |
| Testicular cancer | 0.0480 | 0.0093 | 1224 | 24 |
| Kidney cancer | 0.2120 | 0.0136 | 1224 | 24 |
| Bladder cancer | 0.2342 | 0.0147 | 1224 | 24 |
| Brain and central nervous system cancer | 0.5670 | 0.0631 | 1224 | 24 |
| Thyroid cancer | 0.0558 | 0.0090 | 1224 | 24 |
| Hodgkin lymphoma | 0.1061 | 0.0271 | 1224 | 24 |
| Non-Hodgkin lymphoma | 0.2825 | 0.0485 | 1224 | 24 |
| Multiple myeloma | 0.5100 | 0.0565 | 1224 | 24 |
| Rheumatic heart disease | 0.7480 | 0.1087 | 1224 | 24 |
| Ischemic heart disease | 0.3216 | 0.0763 | 1224 | 24 |
| Ischemic stroke | 0.0578 | 0.0134 | 1224 | 24 |
| Intracerebral hemorrhage | 0.4531 | 0.0529 | 1224 | 24 |
| Subarachnoid hemorrhage | 0.2187 | 0.0537 | 1224 | 24 |
| Atrial fibrillation and flutter | 0.0201 | 0.0018 | 1224 | 24 |
| Endocarditis | 0.0884 | 0.0181 | 1224 | 24 |
| Chronic obstructive pulmonary disease | 0.0710 | 0.0136 | 1224 | 24 |
| Asthma | 0.0018 | 0.0005 | 1224 | 24 |
| Interstitial lung disease and pulmonary sarcoidosis | 0.2498 | 0.0267 | 1224 | 24 |
| Cirrhosis and other chronic liver diseases due to hepatitis C | 0.4131 | 0.0669 | 1224 | 24 |
| Cirrhosis and other chronic liver diseases due to alcohol use | 0.4354 | 0.0665 | 1224 | 24 |
| Cirrhosis and other chronic liver diseases due to other causes | 0.2574 | 0.0524 | 1224 | 24 |
| Peptic ulcer disease | 0.0117 | 0.0031 | 1224 | 24 |
| Gastritis and duodenitis | 0.0001 | 0.0001 | 1224 | 24 |
| Appendicitis | 0.0021 | 0.0006 | 1224 | 24 |
| Paralytic ileus and intestinal obstruction | 0.0069 | 0.0012 | 1224 | 24 |
| Inguinal, femoral, and abdominal hernia | 0.0007 | 0.0001 | 1224 | 24 |
| Inflammatory bowel disease | 0.0089 | 0.0023 | 1224 | 24 |
| Vascular intestinal disorders | 0.0530 | 0.0107 | 1224 | 24 |
| Gallbladder and biliary diseases | 0.0040 | 0.0008 | 1224 | 24 |
| Pancreatitis | 0.0149 | 0.0043 | 1224 | 24 |
| Alzheimer's disease and other dementias | 0.1653 | 0.0161 | 1224 | 24 |
| Parkinson's disease | 0.1576 | 0.0163 | 1224 | 24 |
| Idiopathic epilepsy | 0.0148 | 0.0040 | 1224 | 24 |
| Multiple sclerosis | 0.2861 | 0.0582 | 1224 | 24 |
| Alcohol use disorders | 0.0037 | 0.0014 | 1224 | 24 |
| Opioid use disorders | 0.0500 | 0.0375 | 1224 | 24 |
| Cocaine use disorders | 0.0313 | 0.0198 | 1224 | 24 |
| Amphetamine use disorders | 0.0117 | 0.0088 | 1224 | 24 |
| Anorexia nervosa | 0.0005 | 0.0002 | 1224 | 24 |
| Bulimia nervosa | <0.0001 | <0.0001 | 1224 | 24 |
| Acute glomerulonephritis | 0.0009 | 0.0006 | 1224 | 24 |
| Chronic kidney disease due to hypertension | 0.2514 | 0.0730 | 1224 | 24 |
| Chronic kidney disease due to glomerulonephritis | 0.0573 | 0.0169 | 1224 | 24 |
| Chronic kidney disease due to other and unspecified causes | 0.0056 | 0.0016 | 1224 | 24 |
| Urolithiasis | <0.0001 | <0.0001 | 1224 | 24 |
| Uterine fibroids | 0.0001 | <0.0001 | 1224 | 24 |
| Polycystic ovarian syndrome | <0.0001 | <0.0001 | 1224 | 24 |
| Endometriosis | <0.0001 | <0.0001 | 1224 | 24 |
| Genital prolapse | <0.0001 | <0.0001 | 1224 | 24 |
| Other gynecological diseases | <0.0001 | <0.0001 | 1224 | 24 |
| G6PD deficiency | 0.0029 | 0.0009 | 1224 | 24 |
| Rheumatoid arthritis | 0.0161 | 0.0040 | 1224 | 24 |
| Congenital heart anomalies | 0.0901 | 0.0221 | 1224 | 24 |
| Congenital musculoskeletal and limb anomalies | 0.0061 | 0.0016 | 1224 | 24 |
| Urogenital congenital anomalies | 0.0377 | 0.0104 | 1224 | 24 |
| Digestive congenital anomalies | 0.0350 | 0.0095 | 1224 | 24 |
| Cellulitis | 0.0001 | <0.0001 | 1224 | 24 |
| Pyoderma | 0.0002 | 0.0001 | 1224 | 24 |
| Decubitus ulcer | 0.0019 | 0.0010 | 1224 | 24 |
| Other skin and subcutaneous diseases | <0.0001 | <0.0001 | 1224 | 24 |
| Pedestrian road injuries | 0.0233 | 0.0054 | 1224 | 24 |
| Cyclist road injuries | 0.0022 | 0.0006 | 1224 | 24 |
| Motorcyclist road injuries | 0.0137 | 0.0029 | 1224 | 24 |
| Motor vehicle road injuries | 0.0150 | 0.0043 | 1224 | 24 |
| Other road injuries | 0.0119 | 0.0066 | 1224 | 24 |
| Other transport injuries | 0.0083 | 0.0041 | 1224 | 24 |
| Falls | 0.0009 | 0.0002 | 1224 | 24 |
| Fire, heat, and hot substances | 0.0083 | 0.0026 | 1224 | 24 |
| Poisoning by carbon monoxide | 0.0251 | 0.0100 | 1224 | 24 |
| Poisoning by other means | 0.0035 | 0.0013 | 1224 | 24 |
| Unintentional firearm injuries | 0.0105 | 0.0040 | 1224 | 24 |
| Other exposure to mechanical forces | 0.0005 | 0.0002 | 1224 | 24 |
| Adverse effects of medical treatment | 0.0006 | 0.0003 | 1224 | 24 |
| Venomous animal contact | 0.0003 | 0.0002 | 1224 | 24 |
| Non-venomous animal contact | 0.0002 | 0.0001 | 1224 | 24 |
| Pulmonary aspiration and foreign body in airway | 0.0638 | 0.0191 | 1224 | 24 |
| Foreign body in other body part | 0.0006 | 0.0005 | 1224 | 24 |
| Other unintentional injuries | 0.0003 | 0.0001 | 1224 | 24 |
| Self-harm by other specified means | 0.0299 | 0.0071 | 1224 | 24 |
| Physical violence by firearm | 0.1451 | 0.0560 | 1224 | 24 |
| Physical violence by sharp object | 0.0078 | 0.0026 | 1224 | 24 |
| Physical violence by other means | 0.0027 | 0.0007 | 1224 | 24 |
| Environmental heat and cold exposure | 0.0077 | 0.0035 | 1224 | 24 |
| Chronic lymphoid leukemia | 0.2888 | 0.0577 | 1224 | 24 |
| Acute myeloid leukemia | 0.8256 | 0.0893 | 1224 | 24 |
| Non-melanoma skin cancer (squamous-cell carcinoma) | 0.0048 | 0.0025 | 1224 | 24 |
| Myocarditis | 0.0154 | 0.0044 | 1224 | 24 |
| Other leukemia | 0.5073 | 0.0668 | 1224 | 24 |
| Myelodysplastic, myeloproliferative, and other hematopoietic neoplasms | 0.2305 | 0.0343 | 1224 | 24 |
| Other benign and in situ neoplasms | 0.0001 | <0.0001 | 1224 | 24 |
| Non-rheumatic calcific aortic valve disease | 0.0032 | 0.0007 | 1224 | 24 |
| Non-rheumatic degenerative mitral valve disease | 0.0008 | 0.0002 | 1224 | 24 |
| Diabetes mellitus type 1 | 0.2650 | 0.0754 | 1224 | 24 |
| Diabetes mellitus type 2 | 0.0284 | 0.0080 | 1224 | 24 |
| Maternal abortion and miscarriage | 0.0003 | 0.0004 | 1224 | 24 |
| Chronic kidney disease due to diabetes mellitus type 2 | 0.0703 | 0.0250 | 1224 | 24 |

# Section 3. Spending and covariates over time, 1991-2014

This section includes a descriptive comparison of baseline (1991) and endline (2014) estimates across states for the primary independent variable, personal health care spending, as well as for all covariates included in the model. **Figures 3.1- 3.8** display states with the highest level of spending (or the respective covariate) in 2014 at the top of the graph to the state with the lowest spending or covariate level in 2014 at the bottom of the graph. For example, **Figure 3.1** shows that District of Columbia had the highest unadjusted health care spending per capita in 2014 ($13,600 per person) while Utah had the lowest ($6467 per person). After adjusting for differences in regional prices as done in our final model of value (**Figure 3.2**), North Dakota had the highest level of spending in 2014 and Hawaii had the lowest level of spending. **Figure 3.3** shows that Florida had the greatest percentage of the population over 65 years of age in 2014 (18%) while Alaska had the lowest percentage over 65 years old (9%).

**3.1. Personal health care spending per capita (Current US $)**


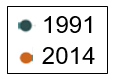

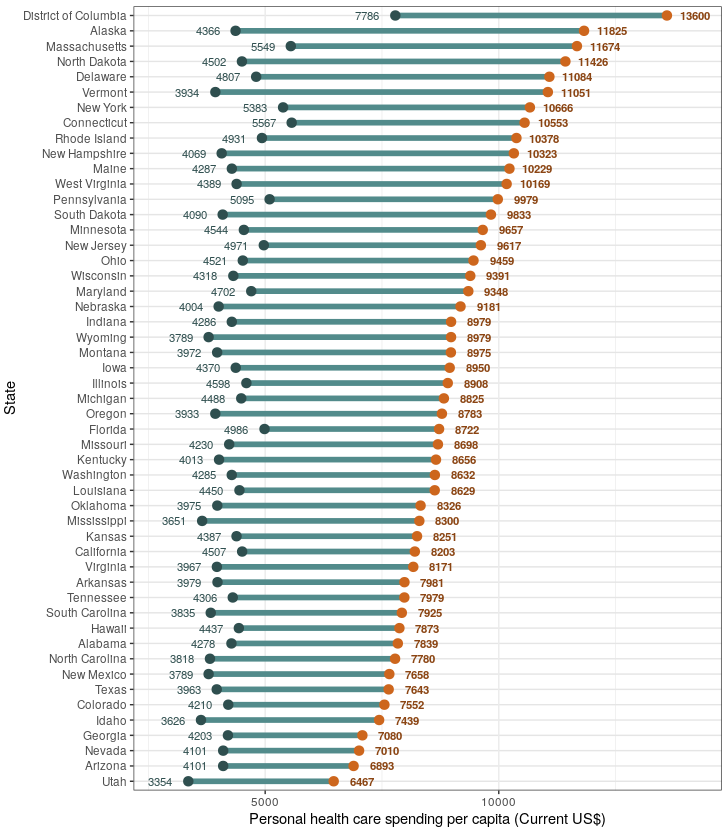


**3.2. Personal health care spending per capita (Regional price-adjusted 2019 US$)**


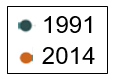

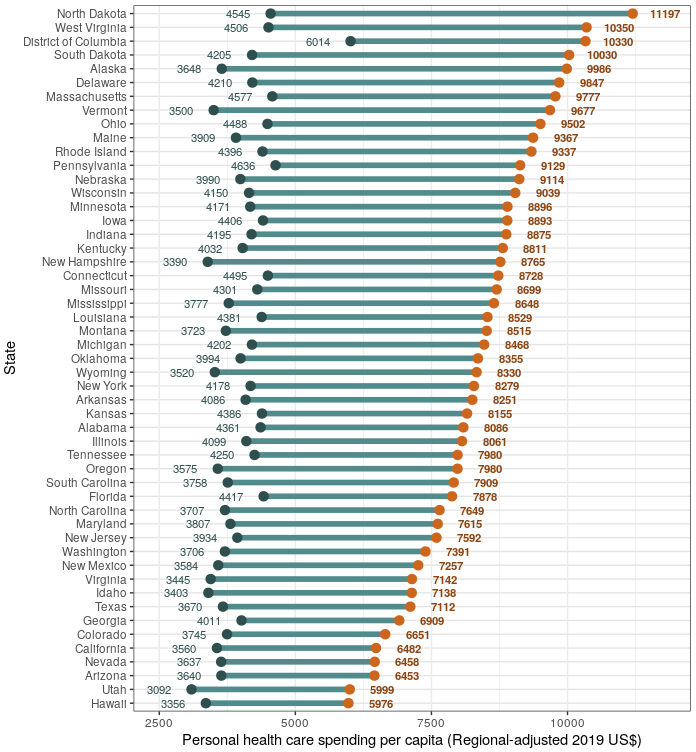


**3.3. Proportion of the population over 65**


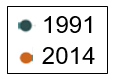

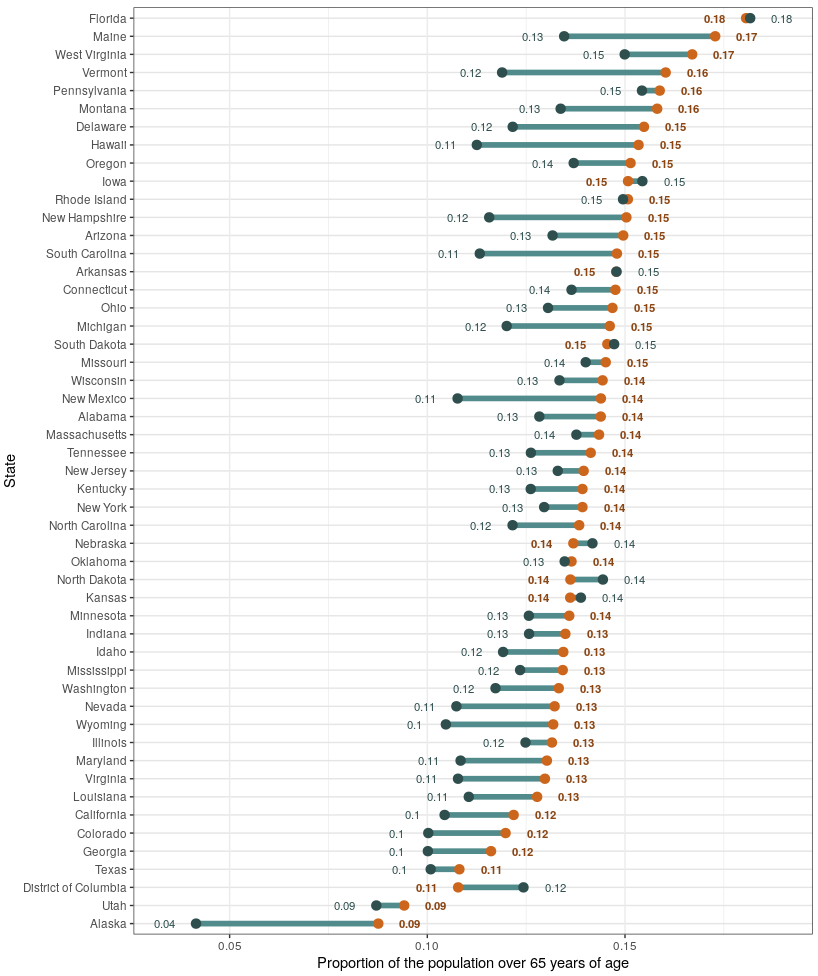


**3.4. Cigarette consumption per capita**


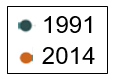

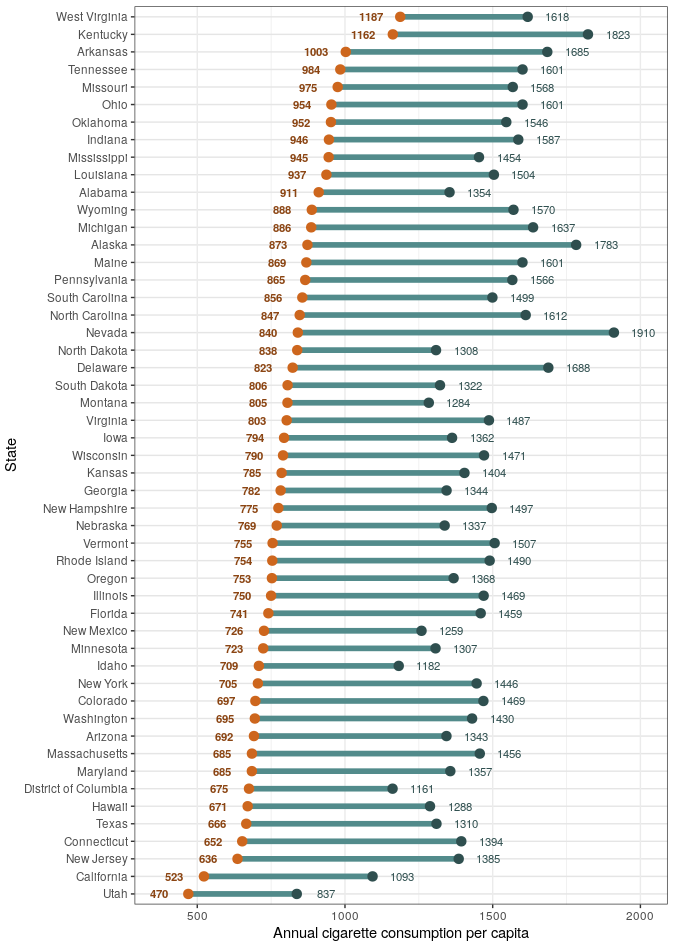


**3.5. Average years of education**


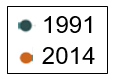

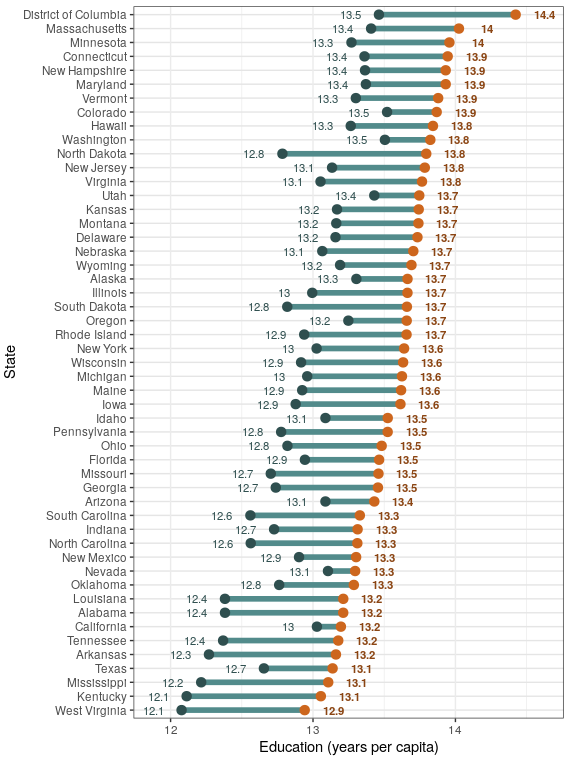


**3.6. Prevalence of obesity**


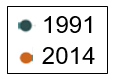

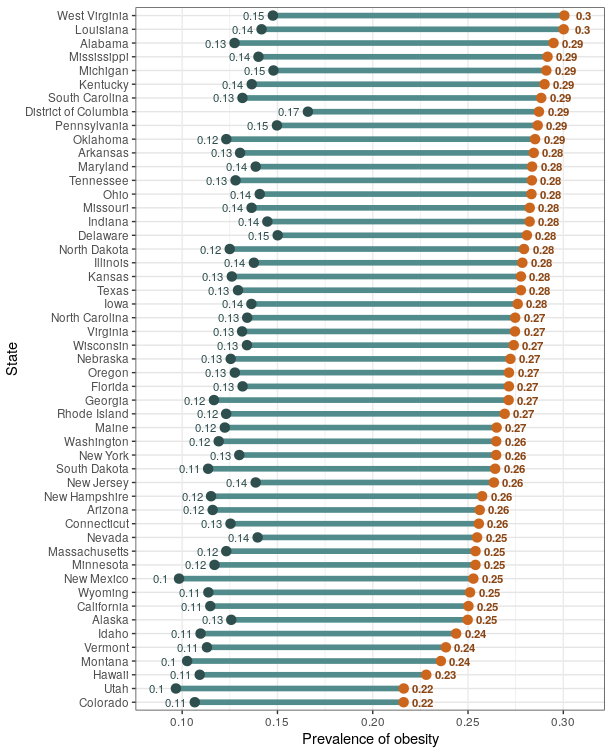


**3.7. Minutes of physical activity per week**


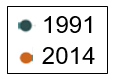

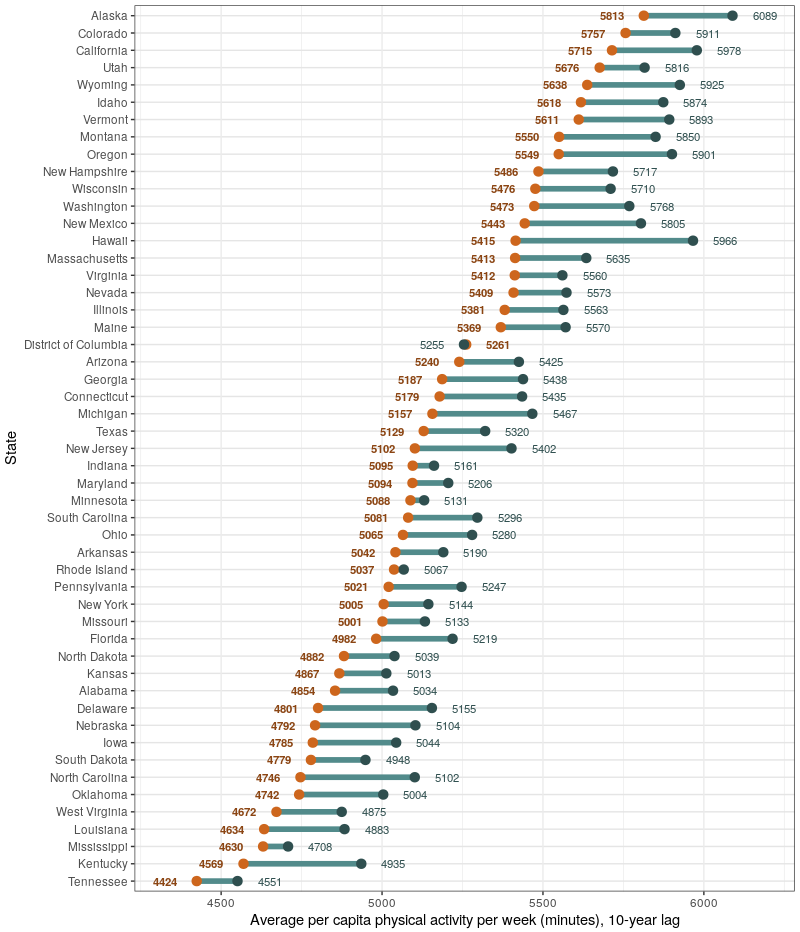


**3.8. Proportion of the population white and non-Hispanic**


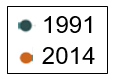

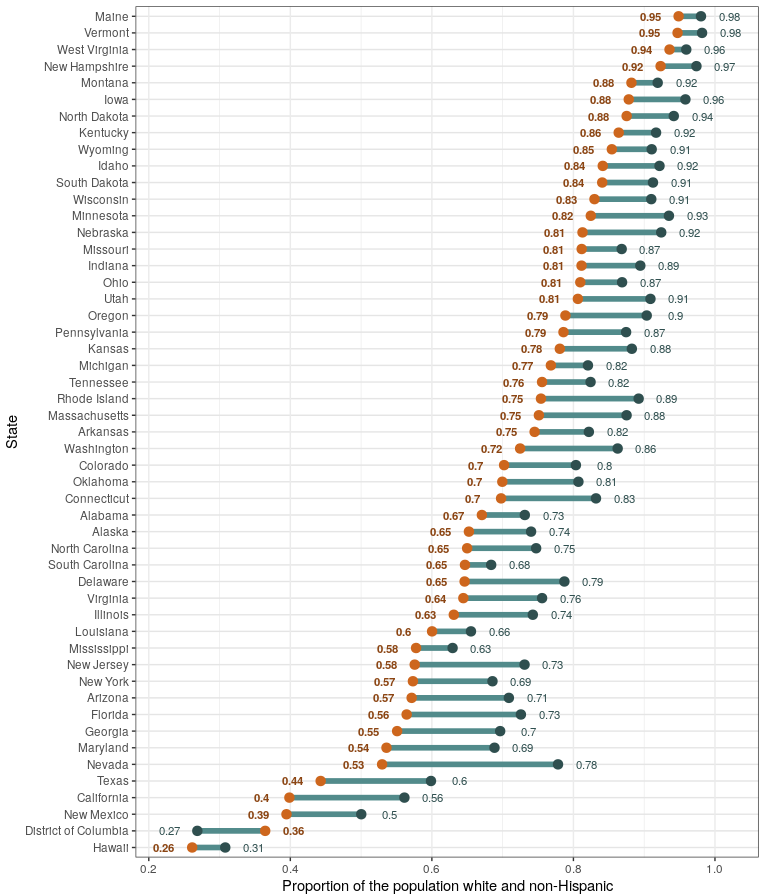


# Section 4. Frontier Analysis Methodology

**4.1. Stochastic Frontier Meta-Analysis (SFM)**

Stochastic frontier analysis (SFA)^8^ is a stochastic analysis of the frontier production function, which expresses the maximum amount of output obtainable from a linear combination of variables of interest. The SFA model we start with is given by


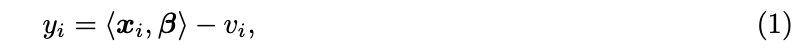


where *y_i_* are observations, *<x_i_, β>*  is the linear model (linear combination of variables in ***x****_i_* with weights ***β***), while *v_i_* is the deviation either from the *maximum output*, modeled as a *non-negative* random effect, or *minimum output*, modeled as a *non-positive* random effect. We focus on deviations from the maximum below, with the analysis for deviation from minimum completely analogous.

This section of the Supplement provides technical details for the Stochastic Frontier Meta-analysis (SFM) extension. Every observation *y_i_* is subject to random error (computed from aggregated data). We consider the model


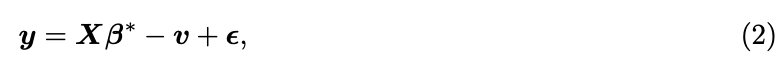


with each entry *v_i_* of ***v*** a half-normal non-negative random effect with unknown variance *η*, while each entry e*_i_* of e is Gaussian N (0*,σ*^2^_i_), and represents the reported study-specific error sources with known variances *σ*^2^_i_.

**Roadmap.** The remainder of this section of the Supplement proceeds as follows. In Section 3.2 we formulate the likelihood problem for the SFM model, assuming a half-normal model for the non-negative random effects *v_i_*.  In Section 3.3, we describe spline models, as well as how to impose priors and constraints on the estimation parameters. Finally, in Section 3.4 we discuss the robust trimming extension that is used to guard against outliers. The fitting procedure is detailed in Section 3.5, and closed form solutions for the inefficiencies are given in Section 3.6.

**4.2. SFM: modeling non-negative random effects**

In this section we derive all likelihood formulations for the Stochastic Frontier Meta-analysis (SFM) approach. We use the half-normal model for the random effects *v_i_*:


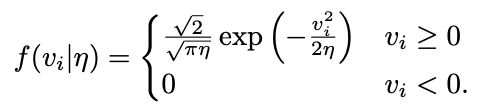


The goal is to estimate ***β***∗  and *η*∗  from observations. The mixed effects framework provides a natural statistical model which can be used for this inference. The joint distribution of fixed and random effects is then given by


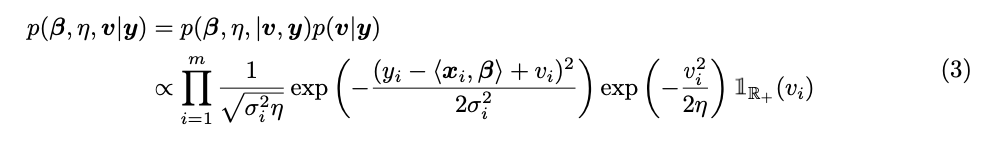


Integrating out the random effects, and taking the negative log of the resulting distribution, we arrive at equivalent maximum likelihood formulation that does not depend on the random effects ***v***, but only depends on ***β*** and *η*. Define Φ to be the complementary error function


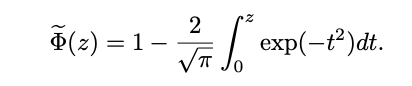


Then we have the following closed form likelihood.


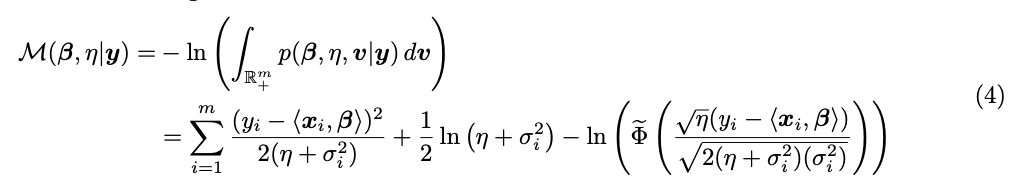


The SFM approach optimizes these likelihoods to estimate (***β****,η*).

**4.3. Priors, Constraints, and Splines.**

In this section we describe how to set up Bayesian priors, constraints for parameters of interest, and spline models for nonlinear relationships in the SFM setup.

**4.3.1. Priors**

The likelihood M can be updated using prior information.  Imposing priors is equivalent to adding penalties to the likelihood function. For the SFM analysis, the only priors we use are those related to the final section of the frontier.

Given a Gaussian prior on ***β*** ∼ *N* (***β***), we find the *a posteriori* estimate by solving the problem


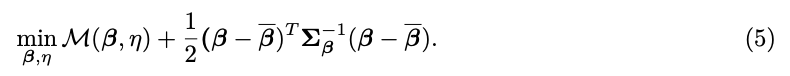


**4.3.2. Constraints**

We allow box constraints and general linear inequality constraints on (***β****,η*). Taking (5) as a running example, we can impose constraints of the form


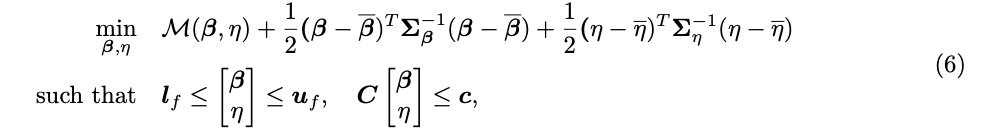


where (***l****_f_ ,* ***u****_f_* ) are lower and upper bounds on the variables, while ***C*** is any matrix.  This functionality can be used to impose shape constraints on spline models, including increasing/decreasing, con- vex/concave, and combinations of these designs.

**4.3.3. Splines**

In this section we discuss spline models for dose-response relationships.  For general background on splines and spline regression see De Boor et al. 1978^9^ and Friedman 1991.^10^

**B-splines and bases.** A spline basis is a set of piecewise polynomial functions with designated degree and domain. If we denote polynomial order by *p*, and the number of knots by *k*, we need *p* + *k* basis elements *s^p^*_j_, which can be generated recursively as illustrated in Figure 1.

Given such a basis, we can represent any dose-response relationship as the linear combination of the spline basis elements, with coefficients ***β*** ∈ R*^p^*^+^*^k^* :


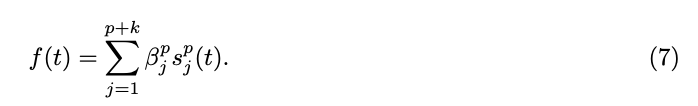


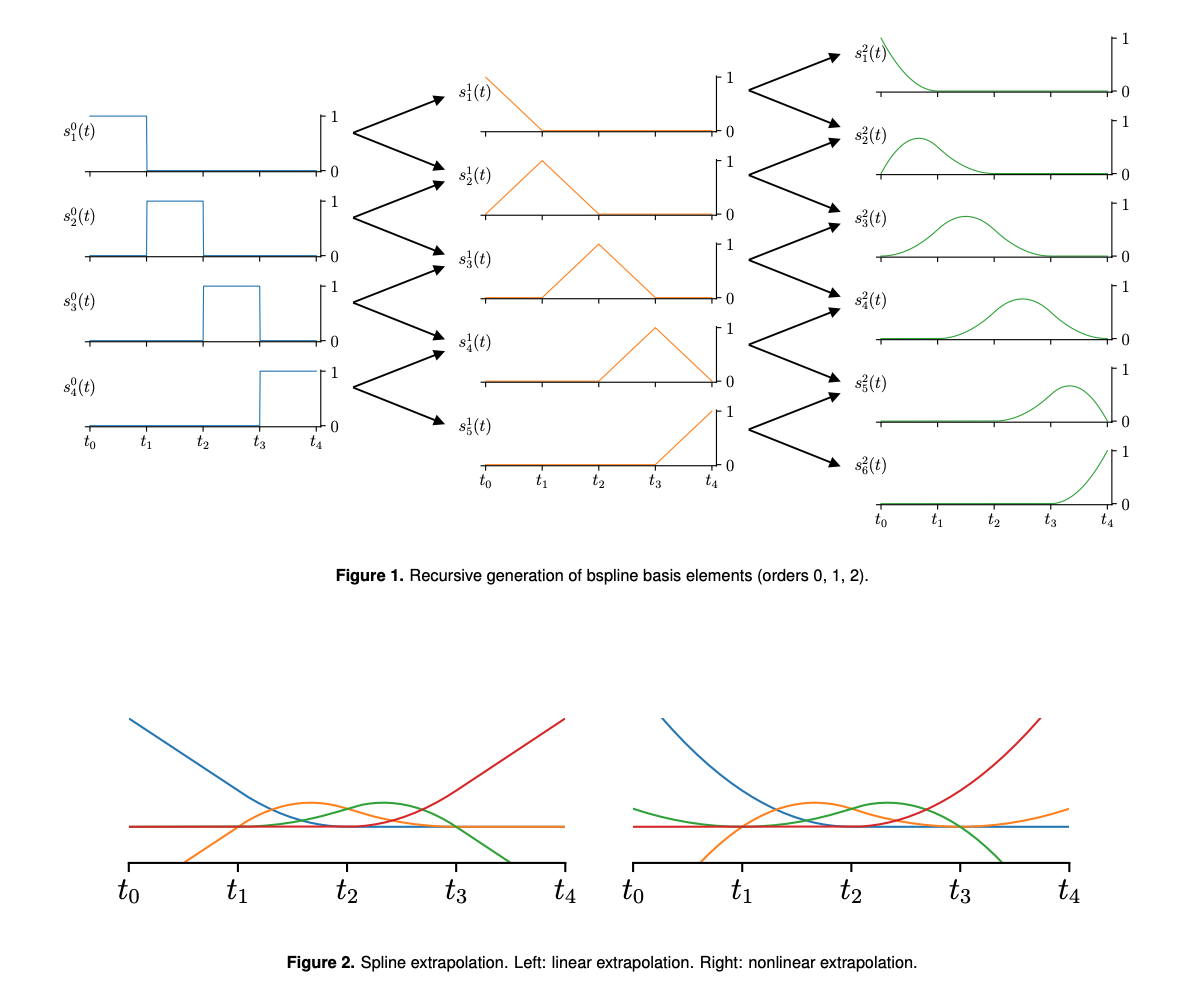


An explicit representation of (7) is obtained by building a design matrix **X**. Given a set of *t* values at which we have data, the *j*th column of **X** is given by the expression


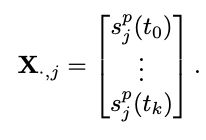


The model for direct observations data coming from the spline (7) can now be written compactly as


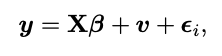


and has the same form as (1).

**Shape constraints.** We can use constraints to enforce monotonicity, convexity, and concavity. Monotonicity across the domain of interest follows from monotonicity of the spline coefficients. This relationship is derived for particular basis constructions elsewhere^9^, and has been used previously in the literature to enforce shape constraints^11^. Current approaches work around the natural inequality constraints by using additional ‘exponentiated’ variables. Instead we impose these constraints directly as described below.

Focusing just on α, the relationship α_1_ <= α_2_ can be written as α_1_ – α_2_ <= 0. Stacking these inequality constraints for each pair (α_i_, α_i+1_) we can write all constraints simultaneously as


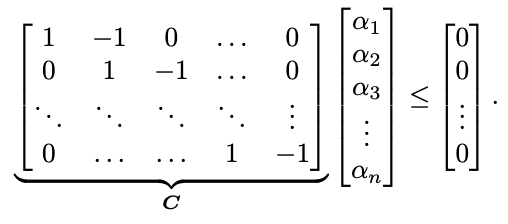


These constraints are directly imposed through the IPOPT interface, along with any lower- and upper- limit constraints on *α*.

**Convexity and Concavity.** For any C^2^ (twice continuously differentiable) function *f* : R → R, convexity and concavity are captured by the signs of the second derivative.  Specifically, *f* is convex if *f”*(*t*) ≥ 0 is everywhere, an concave if *f”*(*t*) ≤ 0 everywhere. We impose linear inequality constraints on the expressions for *f”*(*t*) over each interval.  We can therefore easily pick any of the eight shape combinations given in Pya et al. 2015, Table 1^11^, as well as imposing any other constraints on *α* (including bounds).

**4.4. Optimization**

The SFM model is fit using an algorithm based on variable projection^12–14^, which allows us to leverage a third-party solver, IPOPT^15^ to optimize over ***θ***, significantly reducing complexity. In particular, defining the value function *v*(***w***) by


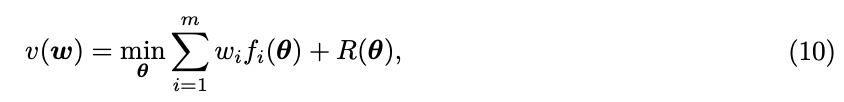


the top level algorithm is simply a projected gradient method to solve


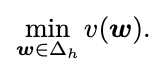


The projection onto the capped simplex is detailed elsewhere.^16^

**4.5. Estimating Random Effects (Inefficiencies)**

Once fixed effects ***θ*** have been estimated, we want to obtain estimates of inefficiency from the joint likelihood (3). We optimize


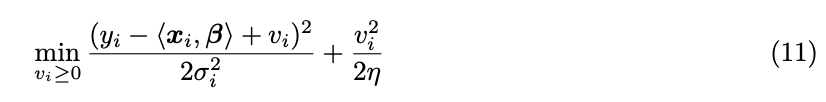


We get the closed form solution


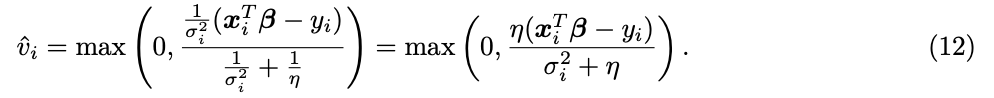


# Section 5. Primary Frontier Analysis - Estimated Covariate Coefficients

This section summarizes the regression coefficients from the meta-stochastic frontier model. For each condition-specific model, a health spending frontier was estimated and the covariate coefficients were evaluated. Coefficients whose direction did not correspond with our prior were dropped from the condition-specific model and the model was rerun. We expected higher levels of obesity, cigarettes consumed per capita, and proportion of the population older than 65 to be associated with higher mortality-incidence ratios. Education, physical activity, and proportion of the population identifying as non-Hispanic white were assumed to be associated with lower mortality-incidence ratios. **Table 5.1** summarizes the number of models (out of the 1000 models run per cause) included each covariate. **Table 5.2** summarizes the mean value of each covariate, by cause, for the models in which it was included.

**Table 5.1.** Number of Models (out of N=1000) Including Named Covariate, by Health Condition

|  | Number of models using covariate | | | | | |
| --- | --- | --- | --- | --- | --- | --- |
| Health condition | % population over 65 | Cigarettes per capita | Education | Obesity | % population non-Hispanic white | Physical activity |
| Acute glomerulonephritis | 778 | 557 | 64 | 845 | 821 | 971 |
| Acute hepatitis A | 510 | 180 | 154 | 848 | 975 | 995 |
| Acute hepatitis B | 778 | 281 | 166 | 876 | 691 | 897 |
| Acute hepatitis C | 549 | 210 | 133 | 851 | 749 | 952 |
| Acute hepatitis E | 569 | 238 | 160 | 752 | 579 | 927 |
| Acute myeloid leukemia | 976 | 321 | 771 | 966 | 40 | 283 |
| Adverse effects of medical treatment | 479 | 228 | 47 | 484 | 836 | 991 |
| Alcohol use disorders | 91 | 233 | 201 | 754 | 665 | 890 |
| Alzheimer's disease and other dementias | 270 | 440 | 622 | 978 | 275 | 907 |
| Amphetamine use disorders | 922 | 480 | 347 | 876 | 816 | 710 |
| Anorexia nervosa | 849 | 289 | 157 | 667 | 625 | 894 |
| Appendicitis | 307 | 247 | 155 | 586 | 479 | 962 |
| Asthma | 435 | 232 | 195 | 527 | 549 | 947 |
| Atrial fibrillation and flutter | 655 | 324 | 414 | 960 | 480 | 810 |
| Bladder cancer | 666 | 253 | 798 | 994 | 435 | 931 |
| Brain and central nervous system cancer | 865 | 702 | 849 | 983 | 325 | 730 |
| Breast cancer | 444 | 241 | 689 | 983 | 231 | 887 |
| Bulimia nervosa | 680 | 93 | 189 | 650 | 977 | 985 |
| Cellulitis | 669 | 170 | 136 | 852 | 925 | 972 |
| Cervical cancer | 914 | 349 | 443 | 846 | 385 | 765 |
| Chlamydial infection | 162 | 397 | 78 | 203 | 954 | 978 |
| Chronic kidney disease due to diabetes mellitus type 2 | 168 | 782 | 599 | 979 | 866 | 962 |
| Chronic kidney disease due to glomerulonephritis | 131 | 784 | 642 | 979 | 846 | 934 |
| Chronic kidney disease due to hypertension | 849 | 881 | 761 | 986 | 774 | 919 |
| Chronic kidney disease due to other and unspecified causes | 162 | 399 | 475 | 956 | 480 | 841 |
| Chronic lymphoid leukemia | 385 | 337 | 754 | 975 | 465 | 776 |
| Chronic obstructive pulmonary disease | 778 | 274 | 607 | 939 | 271 | 665 |
| Cirrhosis and other chronic liver diseases due to alcohol use | 769 | 234 | 841 | 978 | 167 | 488 |
| Cirrhosis and other chronic liver diseases due to hepatitis C | 931 | 268 | 799 | 965 | 199 | 380 |
| Cirrhosis and other chronic liver diseases due to other causes | 889 | 515 | 635 | 950 | 370 | 470 |
| Cocaine use disorders | 936 | 763 | 557 | 976 | 441 | 509 |
| Colon and rectum cancer | 382 | 206 | 773 | 985 | 444 | 807 |
| Congenital heart anomalies | 933 | 305 | 643 | 963 | 380 | 522 |
| Congenital musculoskeletal and limb anomalies | 951 | 222 | 616 | 973 | 312 | 587 |
| Cyclist road injuries | 619 | 269 | 216 | 694 | 486 | 895 |
| Decubitus ulcer | 469 | 339 | 313 | 884 | 517 | 884 |
| Diabetes mellitus type 1 | 958 | 802 | 754 | 971 | 777 | 810 |
| Diabetes mellitus type 2 | 917 | 349 | 444 | 938 | 460 | 765 |
| Diarrheal diseases | 637 | 192 | 376 | 193 | 851 | 960 |
| Digestive congenital anomalies | 877 | 562 | 577 | 951 | 613 | 567 |
| Encephalitis | 187 | 488 | 148 | 776 | 513 | 949 |
| Endocarditis | 559 | 536 | 475 | 940 | 506 | 830 |
| Endometriosis | 805 | 158 | 128 | 770 | 933 | 976 |
| Environmental heat and cold exposure | 104 | 358 | 353 | 864 | 500 | 917 |
| Esophageal cancer | 774 | 332 | 648 | 961 | 433 | 781 |
| Falls | 928 | 173 | 164 | 572 | 546 | 965 |
| Fire, heat, and hot substances | 155 | 420 | 299 | 956 | 662 | 954 |
| Foreign body in other body part | 920 | 290 | 213 | 811 | 786 | 900 |
| G6PD deficiency | 935 | 377 | 404 | 954 | 420 | 777 |
| Gallbladder and biliary diseases | 438 | 175 | 438 | 637 | 285 | 841 |
| Gallbladder and biliary tract cancer | 129 | 491 | 910 | 977 | 617 | 845 |
| Gastritis and duodenitis | 288 | 365 | 44 | 342 | 834 | 988 |
| Genital prolapse | 712 | 96 | 174 | 770 | 977 | 985 |
| Gonococcal infection | 273 | 84 | 363 | 445 | 979 | 979 |
| HIV/AIDS resulting in other diseases | 970 | 648 | 22 | 946 | 834 | 329 |
| Hodgkin lymphoma | 474 | 516 | 744 | 975 | 464 | 762 |
| Idiopathic epilepsy | 658 | 415 | 465 | 968 | 317 | 776 |
| Inflammatory bowel disease | 958 | 283 | 319 | 954 | 559 | 789 |
| Inguinal, femoral, and abdominal hernia | 401 | 203 | 174 | 678 | 569 | 930 |
| Interstitial lung disease and pulmonary sarcoidosis | 906 | 280 | 459 | 762 | 187 | 727 |
| Intracerebral hemorrhage | 375 | 788 | 754 | 975 | 238 | 857 |
| Ischemic heart disease | 369 | 519 | 627 | 955 | 561 | 873 |
| Ischemic stroke | 613 | 540 | 528 | 963 | 375 | 752 |
| Kidney cancer | 967 | 216 | 531 | 948 | 286 | 452 |
| Larynx cancer | 475 | 252 | 804 | 982 | 517 | 580 |
| Lip and oral cavity cancer | 796 | 430 | 653 | 955 | 422 | 556 |
| Liver cancer due to hepatitis C | 258 | 660 | 773 | 979 | 256 | 859 |
| Lower respiratory infections | 575 | 262 | 229 | 631 | 563 | 929 |
| Malignant skin melanoma | 825 | 156 | 779 | 988 | 259 | 834 |
| Maternal abortion and miscarriage | 944 | 278 | 290 | 939 | 602 | 888 |
| Maternal hemorrhage | 684 | 180 | 297 | 653 | 423 | 914 |
| Maternal hypertensive disorders | 773 | 217 | 229 | 826 | 593 | 927 |
| Maternal obstructed labor and uterine rupture | 576 | 198 | 172 | 841 | 796 | 954 |
| Maternal sepsis and other maternal infections | 598 | 179 | 207 | 807 | 805 | 950 |
| Motor vehicle road injuries | 484 | 292 | 528 | 863 | 391 | 867 |
| Motorcyclist road injuries | 974 | 477 | 575 | 947 | 363 | 833 |
| Multiple myeloma | 665 | 372 | 810 | 977 | 186 | 636 |
| Multiple sclerosis | 975 | 439 | 389 | 977 | 259 | 416 |
| Myelodysplastic, myeloproliferative, and other hematopoietic neoplasms | 342 | 647 | 149 | 806 | 338 | 878 |
| Myocarditis | 374 | 325 | 467 | 944 | 648 | 926 |
| Neonatal encephalopathy due to birth asphyxia and trauma | 872 | 255 | 752 | 856 | 208 | 400 |
| Neonatal preterm birth | 850 | 587 | 284 | 951 | 661 | 755 |
| Neonatal sepsis and other neonatal infections | 711 | 630 | 599 | 964 | 706 | 832 |
| Non-Hodgkin lymphoma | 783 | 326 | 702 | 976 | 426 | 781 |
| Non-melanoma skin cancer (squamous-cell carcinoma) | 555 | 397 | 202 | 876 | 484 | 862 |
| Non-rheumatic calcific aortic valve disease | 360 | 275 | 153 | 870 | 814 | 902 |
| Non-rheumatic degenerative mitral valve disease | 501 | 189 | 121 | 630 | 682 | 956 |
| Non-venomous animal contact | 74 | 188 | 158 | 904 | 881 | 969 |
| Opioid use disorders | 924 | 306 | 718 | 898 | 752 | 394 |
| Other benign and in situ neoplasms | 395 | 111 | 193 | 627 | 950 | 984 |
| Other exposure to mechanical forces | 453 | 245 | 164 | 940 | 663 | 959 |
| Other gynecological diseases | 288 | 79 | 154 | 333 | 975 | 986 |
| Other leukemia | 514 | 680 | 921 | 986 | 499 | 555 |
| Other meningitis | 225 | 241 | 579 | 962 | 917 | 960 |
| Other pharynx cancer | 441 | 257 | 774 | 978 | 491 | 798 |
| Other road injuries | 482 | 304 | 628 | 959 | 364 | 505 |
| Other skin and subcutaneous diseases | 283 | 201 | 146 | 624 | 857 | 976 |
| Other transport injuries | 43 | 401 | 501 | 883 | 436 | 675 |
| Other unintentional injuries | 568 | 127 | 91 | 787 | 958 | 986 |
| Otitis media | 114 | 363 | 97 | 163 | 970 | 982 |
| Ovarian cancer | 971 | 365 | 519 | 972 | 228 | 831 |
| Pancreatic cancer | 566 | 631 | 717 | 917 | 191 | 549 |
| Pancreatitis | 214 | 430 | 552 | 931 | 408 | 813 |
| Paralytic ileus and intestinal obstruction | 772 | 293 | 267 | 933 | 560 | 851 |
| Parkinson's disease | 361 | 432 | 582 | 961 | 279 | 859 |
| Pedestrian road injuries | 863 | 360 | 616 | 928 | 595 | 665 |
| Peptic ulcer disease | 764 | 542 | 185 | 888 | 551 | 883 |
| Physical violence by firearm | 294 | 747 | 646 | 965 | 794 | 779 |
| Physical violence by other means | 651 | 246 | 261 | 841 | 593 | 905 |
| Physical violence by sharp object | 714 | 466 | 386 | 911 | 704 | 815 |
| Pneumococcal meningitis | 159 | 413 | 369 | 958 | 592 | 898 |
| Poisoning by carbon monoxide | 142 | 473 | 675 | 850 | 251 | 931 |
| Poisoning by other means | 140 | 247 | 384 | 860 | 445 | 929 |
| Polycystic ovarian syndrome | 547 | 167 | 141 | 338 | 886 | 971 |
| Prostate cancer | 736 | 184 | 697 | 976 | 413 | 753 |
| Protein-energy malnutrition | 195 | 194 | 350 | 874 | 347 | 907 |
| Pulmonary aspiration and foreign body in airway | 563 | 546 | 649 | 900 | 414 | 733 |
| Pyoderma | 333 | 234 | 280 | 635 | 371 | 915 |
| Rheumatic heart disease | 88 | 436 | 933 | 986 | 63 | 156 |
| Rheumatoid arthritis | 926 | 193 | 570 | 975 | 330 | 575 |
| Self-harm by other specified means | 968 | 193 | 58 | 396 | 434 | 652 |
| Stomach cancer | 246 | 267 | 738 | 971 | 591 | 723 |
| Subarachnoid hemorrhage | 263 | 778 | 674 | 967 | 784 | 889 |
| Syphilis | 398 | 231 | 202 | 639 | 581 | 955 |
| Testicular cancer | 335 | 308 | 740 | 985 | 343 | 866 |
| Thyroid cancer | 693 | 566 | 539 | 944 | 342 | 746 |
| Tracheal, bronchus, and lung cancer | 256 | 272 | 705 | 979 | 267 | 827 |
| Unintentional firearm injuries | 122 | 469 | 759 | 951 | 285 | 715 |
| Upper respiratory infections | 231 | 399 | 435 | 271 | 829 | 778 |
| Urogenital congenital anomalies | 700 | 461 | 593 | 966 | 304 | 685 |
| Urolithiasis | 564 | 108 | 168 | 749 | 970 | 981 |
| Uterine cancer | 708 | 316 | 689 | 974 | 579 | 807 |
| Uterine fibroids | 644 | 181 | 242 | 724 | 404 | 913 |
| Varicella and herpes zoster | 761 | 150 | 136 | 695 | 964 | 974 |
| Vascular intestinal disorders | 449 | 422 | 605 | 965 | 358 | 647 |
| Venomous animal contact | 680 | 289 | 311 | 924 | 554 | 900 |

**Table 5.2.** Mean Value for Included Covariates, by Health Condition

|  | Coefficient estimate | | | | | |
| --- | --- | --- | --- | --- | --- | --- |
| Health condition | % population over 65 | Cigarettes per capita | Education | Obesity | % population non-Hispanic white | Physical activity |
| Acute glomerulonephritis | -0.025 | -0.038 | 0.077 | 2.053 | 0.247 | -0.079 |
| Acute hepatitis A | 0.897 | -0.005 | 0.027 | 1.062 | -0.071 | -0.026 |
| Acute hepatitis B | 0.158 | -0.010 | 0.002 | 0.305 | -0.050 | -0.016 |
| Acute hepatitis C | 0.014 | -0.035 | 0.006 | 0.173 | -0.047 | -0.019 |
| Acute hepatitis E | 0.508 | -0.008 | 0.004 | 0.636 | -0.038 | -0.019 |
| Acute myeloid leukemia | 1.458 | 0.077 | 0.019 | 0.531 | 0.367 | -0.007 |
| Adverse effects of medical treatment | 0.364 | 0.005 | 0.019 | 1.325 | 0.066 | -0.031 |
| Alcohol use disorders | 0.043 | -0.004 | 0.005 | 0.525 | -0.147 | -0.048 |
| Alzheimer's disease and other dementias | 3.604 | 0.037 | 0.023 | 3.700 | 0.135 | -0.023 |
| Amphetamine use disorders | 0.253 | -0.008 | 0.002 | 0.827 | -0.072 | -0.027 |
| Anorexia nervosa | 0.285 | -0.040 | -0.008 | 0.239 | -0.041 | -0.016 |
| Appendicitis | 0.916 | -0.036 | 0.014 | 1.031 | -0.059 | -0.033 |
| Asthma | -0.071 | -0.052 | 0.924 | 0.575 | -0.008 | -0.154 |
| Atrial fibrillation and flutter | 0.991 | -0.005 | -0.005 | 0.693 | -0.029 | -0.069 |
| Bladder cancer | 2.328 | 0.061 | -0.014 | 3.869 | 0.081 | -0.013 |
| Brain and central nervous system cancer | 1.554 | 0.009 | -0.027 | 3.718 | 0.050 | -0.014 |
| Breast cancer | 2.663 | -0.028 | -0.007 | 3.021 | 0.363 | -0.021 |
| Bulimia nervosa | -0.193 | -0.013 | 0.274 | -0.355 | -0.138 | -0.149 |
| Cellulitis | 0.079 | -0.046 | 0.015 | 0.704 | -0.011 | -0.012 |
| Cervical cancer | 5.283 | -0.008 | 0.018 | 10.794 | -0.298 | -0.060 |
| Chlamydial infection | -0.003 | -0.008 | -0.007 | -0.002 | -0.016 | -0.006 |
| Chronic kidney disease due to diabetes mellitus type 2 | 1.175 | -0.128 | 0.048 | 5.921 | -0.376 | -0.238 |
| Chronic kidney disease due to glomerulonephritis | 0.453 | -0.041 | 0.090 | 3.955 | -0.212 | -0.200 |
| Chronic kidney disease due to hypertension | 1.617 | 0.039 | 0.000 | 2.921 | -0.057 | -0.029 |
| Chronic kidney disease due to other and unspecified causes | 0.565 | -0.005 | 0.009 | 0.790 | -0.025 | -0.033 |
| Chronic lymphoid leukemia | 5.253 | 0.127 | 0.003 | 7.976 | -0.218 | -0.056 |
| Chronic obstructive pulmonary disease | 0.723 | 0.004 | 0.003 | 1.470 | 0.012 | -0.028 |
| Cirrhosis and other chronic liver diseases due to alcohol use | 4.312 | 0.054 | -0.017 | 7.548 | 0.383 | -0.039 |
| Cirrhosis and other chronic liver diseases due to hepatitis C | 6.296 | 0.187 | -0.045 | 12.251 | 0.512 | 0.003 |
| Cirrhosis and other chronic liver diseases due to other causes | 8.444 | 0.054 | 0.039 | 19.693 | -0.412 | -0.235 |
| Cocaine use disorders | 1.097 | -0.007 | 0.002 | 2.317 | -0.067 | -0.036 |
| Colon and rectum cancer | 4.748 | -0.029 | -0.007 | 9.157 | 0.009 | -0.092 |
| Congenital heart anomalies | 7.486 | -0.087 | -0.078 | 15.768 | 0.664 | -0.057 |
| Congenital musculoskeletal and limb anomalies | 0.717 | -0.005 | 0.000 | 1.587 | 0.326 | 0.004 |
| Cyclist road injuries | 0.557 | -0.012 | 0.142 | 1.038 | -0.166 | -0.061 |
| Decubitus ulcer | 1.118 | -0.012 | 0.006 | 0.854 | 0.011 | -0.021 |
| Diabetes mellitus type 1 | 4.669 | 0.038 | -0.013 | 8.972 | -0.095 | -0.036 |
| Diabetes mellitus type 2 | 1.285 | 0.009 | -0.011 | 2.183 | 0.222 | 0.005 |
| Diarrheal diseases | 0.094 | -0.008 | 0.010 | 0.808 | -0.080 | -0.015 |
| Digestive congenital anomalies | 0.554 | 0.004 | -0.001 | 1.211 | -0.017 | -0.007 |
| Encephalitis | 0.338 | 0.001 | 0.054 | 2.383 | -0.037 | -0.030 |
| Endocarditis | 14.236 | -0.004 | 0.044 | 24.345 | -1.174 | -0.262 |
| Endometriosis | 0.078 | -0.115 | 0.046 | 0.233 | -0.110 | -0.040 |
| Environmental heat and cold exposure | 0.111 | -0.005 | 0.011 | 0.866 | -0.001 | -0.024 |
| Esophageal cancer | 7.765 | 0.046 | -0.020 | 13.357 | 0.097 | -0.001 |
| Falls | 0.065 | -0.011 | 0.003 | 0.175 | -0.033 | -0.011 |
| Fire, heat, and hot substances | 0.134 | -0.008 | 0.017 | 0.592 | -0.030 | -0.028 |
| Foreign body in other body part | 0.019 | 0.019 | -0.054 | -0.099 | -0.030 | -0.009 |
| G6PD deficiency | 1.023 | 0.001 | 0.010 | 0.941 | 0.370 | -0.040 |
| Gallbladder and biliary diseases | 0.443 | -0.032 | 0.020 | 0.630 | -0.038 | -0.031 |
| Gallbladder and biliary tract cancer | 0.163 | 0.007 | -0.018 | 0.934 | -0.051 | -0.013 |
| Gastritis and duodenitis | 0.234 | -0.138 | 0.012 | 1.343 | -0.577 | -0.149 |
| Genital prolapse | 0.025 | -0.015 | 0.122 | 0.095 | -0.079 | -0.039 |
| Gonococcal infection | 0.023 | -0.066 | 0.319 | 0.109 | -0.324 | -0.036 |
| HIV/AIDS resulting in other diseases | 1.794 | -0.111 | 0.067 | 2.643 | -0.095 | -0.105 |
| Hodgkin lymphoma | 5.390 | 0.000 | 0.051 | 9.532 | -0.307 | -0.220 |
| Idiopathic epilepsy | 2.917 | 0.008 | 0.006 | 6.479 | -0.048 | -0.039 |
| Inflammatory bowel disease | 0.312 | -0.009 | 0.019 | 0.908 | -0.098 | -0.034 |
| Inguinal, femoral, and abdominal hernia | -0.072 | -0.157 | 0.010 | 0.491 | -0.193 | -0.053 |
| Interstitial lung disease and pulmonary sarcoidosis | 3.635 | 0.073 | 0.006 | 6.569 | 0.282 | -0.028 |
| Intracerebral hemorrhage | 2.196 | 0.032 | 0.016 | 6.437 | -0.101 | -0.078 |
| Ischemic heart disease | 9.380 | -0.112 | 0.179 | 19.192 | 0.241 | -0.351 |
| Ischemic stroke | 0.680 | 0.006 | 0.003 | 2.373 | 0.000 | -0.023 |
| Kidney cancer | 1.553 | 0.009 | -0.011 | 3.256 | 0.166 | -0.028 |
| Larynx cancer | 3.152 | 0.014 | 0.012 | 6.869 | 0.104 | 0.001 |
| Lip and oral cavity cancer | 5.026 | -0.061 | -0.003 | 9.738 | 0.312 | -0.086 |
| Liver cancer due to hepatitis C | 7.094 | 0.070 | -0.053 | 9.689 | -0.016 | -0.032 |
| Lower respiratory infections | 0.400 | -0.024 | -0.004 | 0.573 | -0.012 | -0.020 |
| Malignant skin melanoma | 2.770 | -0.032 | 0.020 | 4.838 | 0.070 | -0.040 |
| Maternal abortion and miscarriage | 0.124 | -0.033 | 0.009 | 0.716 | -0.136 | -0.040 |
| Maternal hemorrhage | 0.428 | -0.018 | 0.034 | 0.616 | -0.015 | -0.026 |
| Maternal hypertensive disorders | -0.128 | -0.013 | 0.004 | 0.054 | -0.139 | -0.024 |
| Maternal obstructed labor and uterine rupture | 0.093 | -0.028 | 0.017 | 0.247 | -0.018 | -0.018 |
| Maternal sepsis and other maternal infections | 0.062 | -0.022 | 0.045 | 0.585 | -0.072 | -0.045 |
| Motor vehicle road injuries | 0.308 | -0.004 | 0.025 | 1.301 | -0.093 | -0.048 |
| Motorcyclist road injuries | 0.878 | -0.011 | 0.019 | 1.301 | -0.165 | -0.031 |
| Multiple myeloma | 1.803 | 0.013 | -0.026 | 2.840 | 0.062 | 0.024 |
| Multiple sclerosis | 3.556 | -0.013 | -0.007 | 4.320 | 0.007 | -0.037 |
| Myelodysplastic, myeloproliferative, and other hematopoietic neoplasms | 8.271 | 0.061 | 0.295 | 11.023 | 0.142 | -0.135 |
| Myocarditis | 0.489 | -0.051 | 0.009 | 1.355 | -0.024 | -0.031 |
| Neonatal encephalopathy due to birth asphyxia and trauma | 1.728 | -0.043 | 0.016 | 5.385 | 0.438 | -0.129 |
| Neonatal preterm birth | 0.927 | -0.012 | 0.007 | 1.789 | -0.034 | -0.033 |
| Neonatal sepsis and other neonatal infections | 5.088 | -0.010 | 0.048 | 13.736 | -0.278 | -0.184 |
| Non-Hodgkin lymphoma | 5.804 | 0.069 | -0.066 | 13.258 | 0.203 | -0.006 |
| Non-melanoma skin cancer (squamous-cell carcinoma) | 1.232 | 0.026 | 0.015 | 1.821 | -0.108 | -0.017 |
| Non-rheumatic calcific aortic valve disease | 0.246 | -0.032 | 0.034 | 1.033 | -0.020 | -0.040 |
| Non-rheumatic degenerative mitral valve disease | 0.105 | 0.006 | 0.039 | 0.558 | -0.024 | -0.025 |
| Non-venomous animal contact | 0.342 | -0.401 | 0.200 | 0.167 | -0.376 | -0.071 |
| Opioid use disorders | 0.750 | -0.003 | -0.014 | 1.592 | 0.019 | 0.020 |
| Other benign and in situ neoplasms | 0.043 | -0.049 | 0.045 | 0.533 | -0.068 | -0.029 |
| Other exposure to mechanical forces | 0.205 | -0.045 | 0.032 | 0.200 | -0.047 | -0.023 |
| Other gynecological diseases | 0.025 | -0.078 | 0.017 | 0.032 | -0.032 | -0.017 |
| Other leukemia | 0.483 | 0.015 | -0.023 | 1.894 | 0.039 | -0.006 |
| Other meningitis | 0.164 | 0.015 | 0.021 | 1.165 | -0.114 | -0.041 |
| Other pharynx cancer | 3.342 | 0.013 | -0.026 | 4.901 | -0.023 | -0.014 |
| Other road injuries | 1.100 | -0.001 | -0.002 | 1.710 | -0.049 | -0.024 |
| Other skin and subcutaneous diseases | -0.007 | -0.019 | 0.129 | 0.077 | -0.160 | -0.049 |
| Other transport injuries | 0.112 | -0.005 | -0.015 | 1.047 | -0.094 | -0.035 |
| Other unintentional injuries | 0.332 | -0.042 | -0.005 | 0.447 | -0.096 | -0.026 |
| Otitis media | 0.148 | -0.062 | 0.008 | 0.555 | -0.267 | -0.074 |
| Ovarian cancer | 6.122 | -0.013 | 0.025 | 9.721 | -0.280 | -0.062 |
| Pancreatic cancer | 5.922 | 0.081 | 0.009 | 8.585 | 0.062 | -0.036 |
| Pancreatitis | 0.564 | 0.012 | 0.030 | 1.263 | -0.085 | -0.057 |
| Paralytic ileus and intestinal obstruction | 0.180 | -0.008 | 0.018 | 1.034 | -0.030 | -0.029 |
| Parkinson's disease | 2.904 | 0.030 | -0.006 | 7.151 | 0.042 | -0.029 |
| Pedestrian road injuries | 0.368 | -0.012 | 0.010 | 1.368 | -0.042 | -0.040 |
| Peptic ulcer disease | 0.840 | -0.029 | 0.045 | 2.403 | -0.075 | -0.073 |
| Physical violence by firearm | 8.526 | 0.081 | 0.160 | 17.819 | -0.406 | -0.263 |
| Physical violence by other means | 0.513 | -0.014 | 0.006 | 0.602 | -0.046 | -0.023 |
| Physical violence by sharp object | 0.673 | -0.019 | 0.004 | 2.041 | -0.060 | -0.049 |
| Pneumococcal meningitis | 1.234 | -0.007 | 0.013 | 3.644 | -0.324 | -0.075 |
| Poisoning by carbon monoxide | 1.188 | 0.007 | 0.106 | 6.845 | -0.110 | -0.157 |
| Poisoning by other means | 0.153 | -0.006 | 0.042 | 0.766 | -0.025 | -0.034 |
| Polycystic ovarian syndrome | 0.059 | 0.000 | 0.000 | 0.106 | -0.007 | -0.007 |
| Prostate cancer | 0.950 | 0.036 | -0.001 | 2.439 | 0.040 | 0.005 |
| Protein-energy malnutrition | 0.048 | -0.018 | 0.011 | 0.455 | -0.026 | -0.020 |
| Pulmonary aspiration and foreign body in airway | 0.815 | -0.008 | 0.105 | 3.185 | -0.247 | -0.213 |
| Pyoderma | 0.242 | -0.049 | 0.008 | 0.433 | -0.066 | -0.025 |
| Rheumatic heart disease | 1.988 | 0.041 | -0.050 | 2.621 | 0.102 | 0.000 |
| Rheumatoid arthritis | 0.815 | 0.003 | 0.002 | 1.571 | 0.091 | -0.017 |
| Self-harm by other specified means | 0.778 | -0.010 | 0.001 | 1.960 | 0.004 | -0.014 |
| Stomach cancer | 3.152 | 0.063 | -0.065 | 8.203 | 0.249 | -0.012 |
| Subarachnoid hemorrhage | 12.981 | -0.084 | 0.049 | 18.789 | -0.200 | -0.268 |
| Syphilis | 0.014 | -0.074 | 0.017 | 0.266 | 0.006 | -0.055 |
| Testicular cancer | 1.145 | 0.000 | 0.004 | 1.632 | -0.017 | -0.020 |
| Thyroid cancer | 0.786 | 0.002 | 0.005 | 1.552 | 0.009 | -0.019 |
| Tracheal, bronchus, and lung cancer | 5.144 | 0.065 | 0.001 | 11.099 | -0.207 | -0.067 |
| Unintentional firearm injuries | 0.548 | 0.004 | 0.005 | 1.052 | -0.069 | -0.027 |
| Upper respiratory infections | -0.336 | 0.523 | 0.372 | -1.065 | 0.132 | -0.170 |
| Urogenital congenital anomalies | 1.420 | 0.017 | 0.004 | 2.982 | 0.186 | -0.008 |
| Urolithiasis | 0.046 | -0.045 | 0.041 | 0.177 | -0.059 | -0.025 |
| Uterine cancer | 2.261 | 0.008 | -0.002 | 3.101 | -0.050 | -0.014 |
| Uterine fibroids | 0.175 | -0.035 | 0.002 | 0.352 | -0.310 | -0.023 |
| Varicella and herpes zoster | 0.176 | -0.103 | 0.025 | 0.332 | -0.144 | -0.046 |
| Vascular intestinal disorders | 0.904 | 0.006 | 0.007 | 1.630 | -0.007 | -0.050 |
| Venomous animal contact | 0.080 | -0.018 | 0.019 | 0.206 | -0.073 | -0.026 |

# Section 6. Comparisons with other estimates of value

This section provides comparisons of this study’s estimates of healthcare value rankings with six other rankings of value found in the literature: (1) Altarum Health Care Affordability Scorecard,^17^ (2) Commonwealth Fund State Scorecard on Health System Performance,^18^ (3) US News & World Report Health Care Rankings,^19^ (4) Money Rates Composite Ranking on Best States for Healthcare,^20^ (5) United Health Foundation America’s Health Rankings,^21^ and (6) WalletHub States with the Best and Worst Health Return on Investment (ROI).^22^ Comparisons with both the final value rankings generated by this study (referred to as Value Ranking, 2014 in the figures) as well as unadjusted value rankings (i.e. 2014 Value Rankings generated without adjusting for covariates) are presented below. Given that the data used for the current study were only available up through 2014, the closest year to 2014 or above was prioritized for comparison. However, as the six peer-estimates listed above were available primarily for 2018-2020 (year of comparison is specified in each summary below), the comparability with this study’s most recent year of data of 2014 may be limited. This section highlights that adjusting for known drivers of health, such as age, obesity rates, and education levels has a large impact, and that by-and-large these this study’s unadjusted estimates are more correlated with other studies’ rankings of value.

**6.1. Altarum Health Care Affordability Scorecard, Overall affordability ranking (2020)**^17^

Altarum’s overall affordability ranking for outcome scores is a composite of four other rankings, focusing primarily on process variables rather than health outcomes: (1) extending coverage to all residents, (2) making out-of pocket costs affordable, (3) reducing low-value care, and (4) addressing excess prices. The outcome indicator for extending coverage to all residents is the total uninsured rate for the state. The indicators for making out-of-pocket costs affordable is the overall prevalence of adults who needed but could not afford medical care, delayed seeking medical care because of worry about cost, made changes to medical drugs because of cost, and had trouble paying bills. The indicators for reducing low-value care are Cesarean section rates among births to first-time, low-risk mothers and the antibiotic prescribing per 1000 residents. The indicator for curbing excess prices in the system is private payer prices relative to the national median. All outcome indicators included are given as a relative score compared with the best state.

**Figure 6.1** Current Study Value Rankings Versus Altarum Scorecard, Adjusted and Unadjusted Results


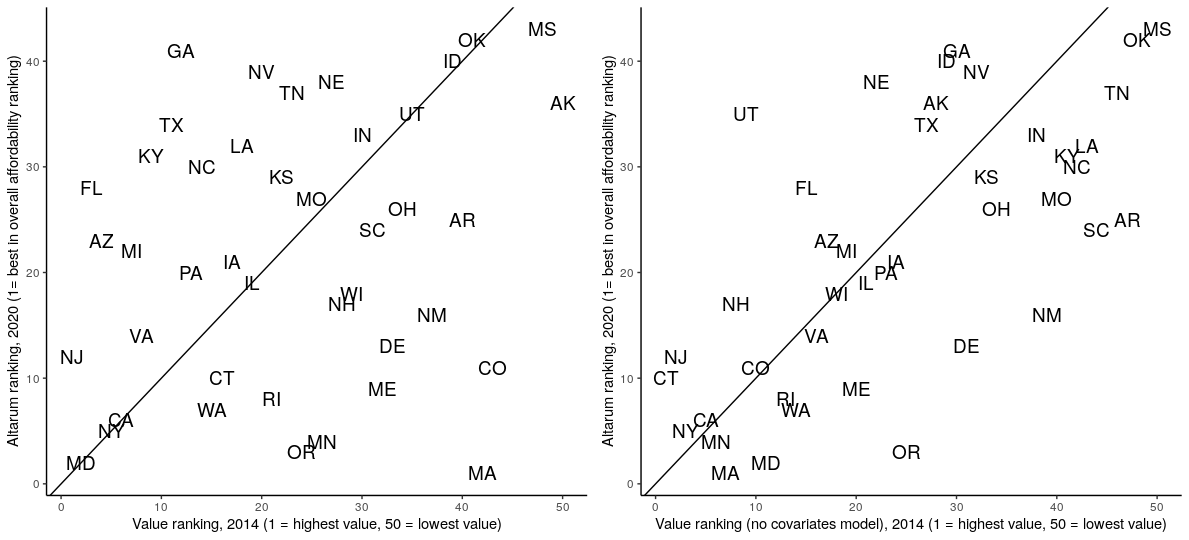


**6.2. Commonwealth Fund State Scorecard on Health System Performance, Overall ranking (2018)**^18^

The Commonwealth Fund State Scorecard creates rankings across several health system domains and focuses primarily on process variables rather than health outcomes, though 5 mortality indicators are also included. The overall ranking is a composite of 43 performance indicators grouped into 4 dimensions: Access and Affordability, Prevention and Treatment, Potentially Avoidable Hospital Use and Cost, and Healthy Lives. Performance indicators include metrics such as uninsured rates and out-of-pocket spending, adults not covered by preventive treatments, potentially avoidable hospital visits and admissions, mortality, and prevalence of risk factors including smoking and obesity.

**Figure 6.2** Current Study Value Rankings Versus Commonwealth Scorecard, Adjusted and Unadjusted Results


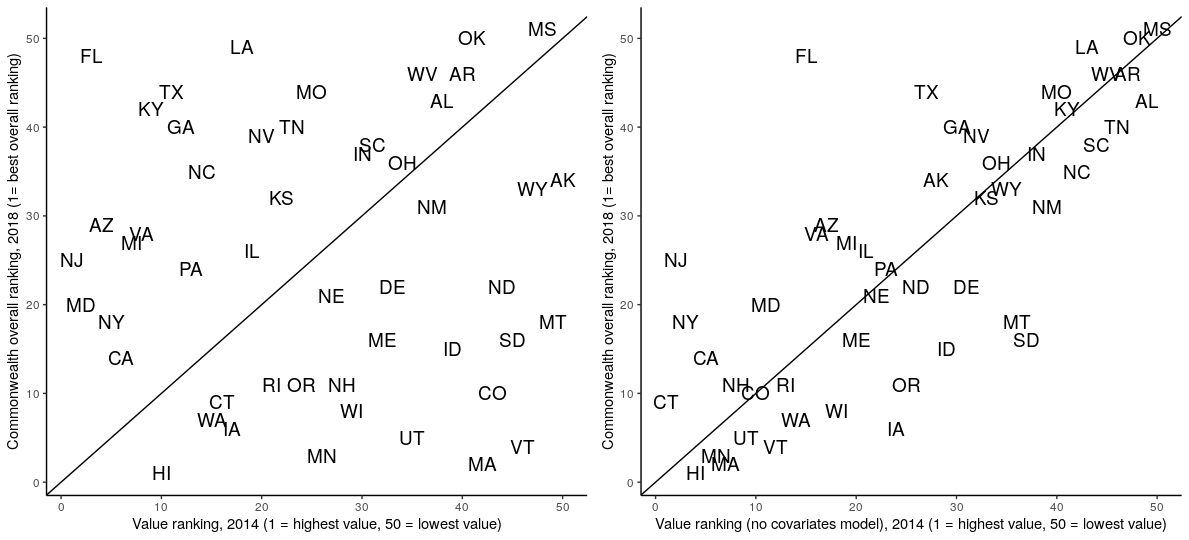


**6.3. US News & World Report Health Care Rankings (2020)**^19^

The US News & World Report Health Care Rankings rank states using health care access, health care quality, and public health as equally-weighted dimensions. This composite ranking is generated from a variety of indicators including both process indicators and health outcomes, and includes sources such as survey responses on ability to pay, US News' in-house algorithm for rankings of hospitals and nursing facilities, Medicare quality, and health risk factors as well as outcomes such as smoking, suicide, infant and all-cause mortality.

**Figure 6.3** Current Study Value Rankings Versus US News & World Report Ranking, Adjusted and Unadjusted Results


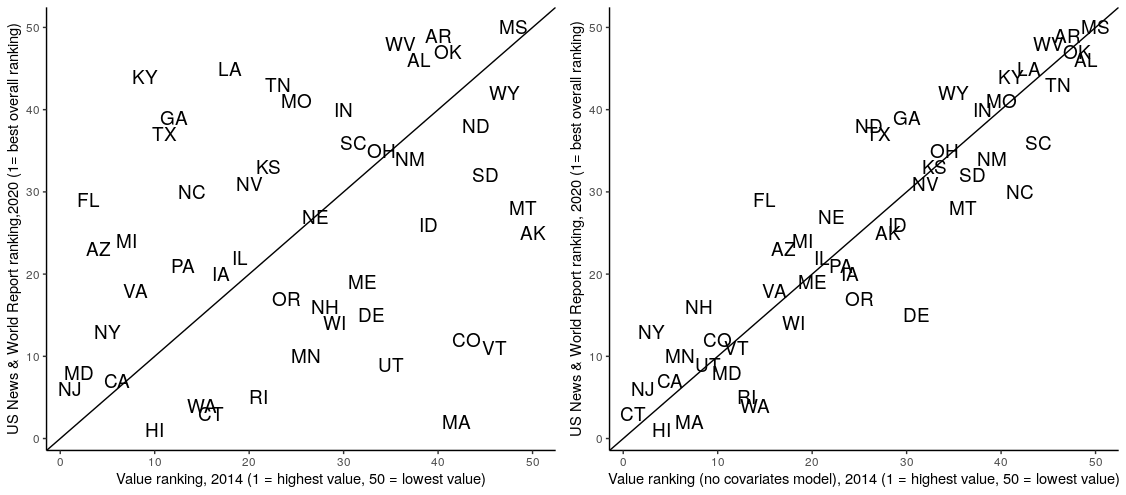


**6.4. MoneyRates Composite Ranking on Best States for Healthcare (2020)**^20^

The MoneyRates Composite Ranking generated by financial analyst Richard Barrington uses primarily process variables obtained from the US Census Bureau and Kaiser Family Foundation to rank states for “overall health care system condition”. Specific health system characteristics entering into the rankings include: Health insurance coverage, Self-reported health, Child immunization coverage, Infant survival rates, Adequacy of nursing home staffing, Adequacy of doctors' office staffing, Daily inpatient expenses, and Annual healthcare premium affordability. Then the mean of each of these rankings is combined into the composite ranking.

**Figure 6.4** Current Study Value Rankings Versus MoneyRates Ranking, Adjusted and Unadjusted Results


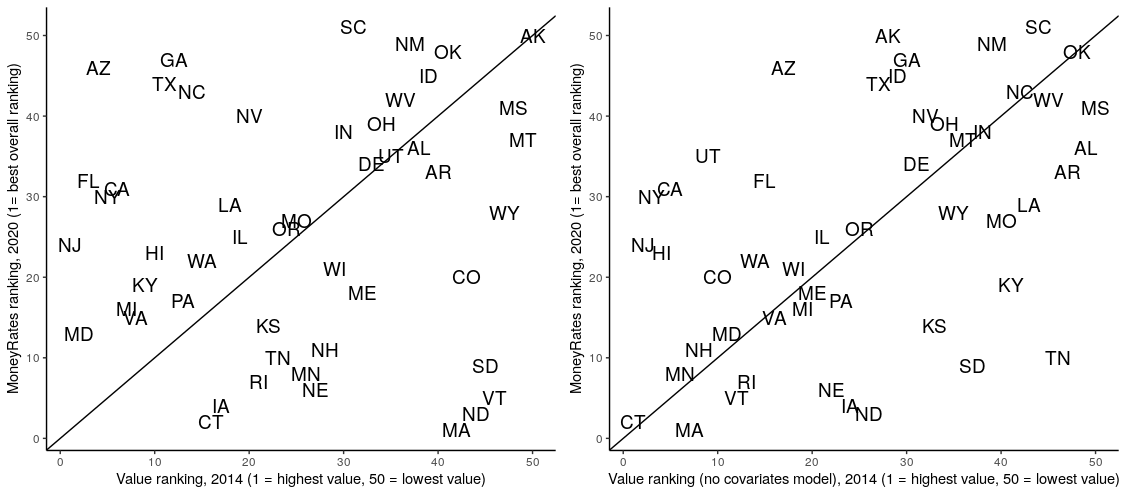


**6.5. United Health Foundation: America's Health Rankings (2014)**^21^

The United Health Foundation America’s Health rankings focus on maximizing health outcomes and do not use a cost component. US states are ranked from healthiest to least healthy using the weighted sum of the number of standard deviations each core outcome is from the national average. This ranking covers 34 indicators across the following six domains: Behaviors (drug deaths, drinking, etc.), Community and Environment (air pollution, infectious diseases, violent crime), Policy (immunization coverage, public health funding and uninsured rates), Clinical care (dentists, preventable hospitalizations, etc.), and Outcomes (cancer deaths, cardiovascular deaths, infant mortality, diabetes, disparity in health status, etc.).

**Figure 6.5** Current Study Value Rankings Versus United Health Foundation Ranking, Adjusted and Unadjusted Results


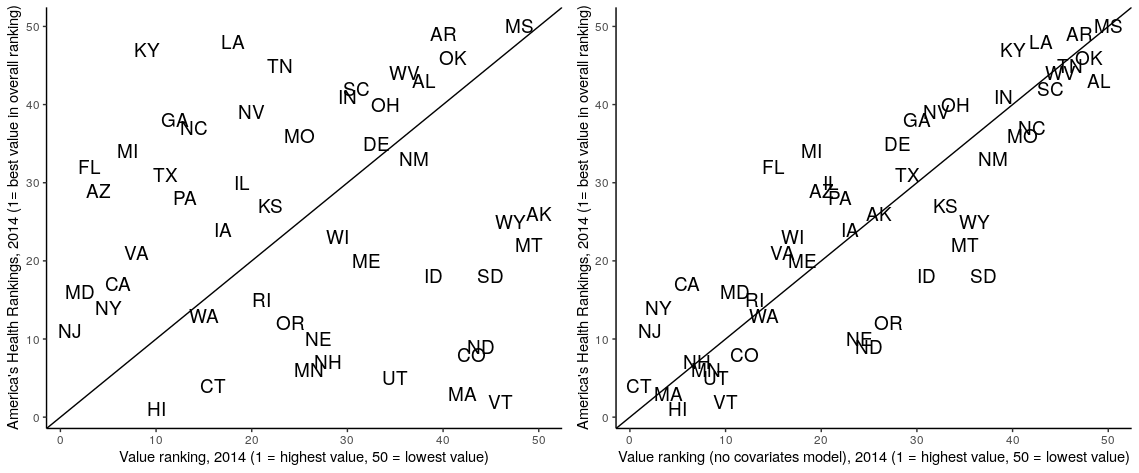


**6.6. WalletHub: States with the Best and Worst Health ROI (2014)**^22^

WalletHub’s ranking of States with the Best and Worst Health ROI, published by financial writer Richie Bernardo, is generated using three key metrics: age-standardized death rates and America’s Health Rankings (from **Section 6.5** above) are used as indicators of quality, and average individual health insurance premiums is used to estimate costs. Health ROI is then generated by dividing the Quality of Health Ranking by the Health Care Costs Ranking.

**Figure 6.6** Current Study Value Rankings Versus WalletHub Ranking, Adjusted and Unadjusted Results


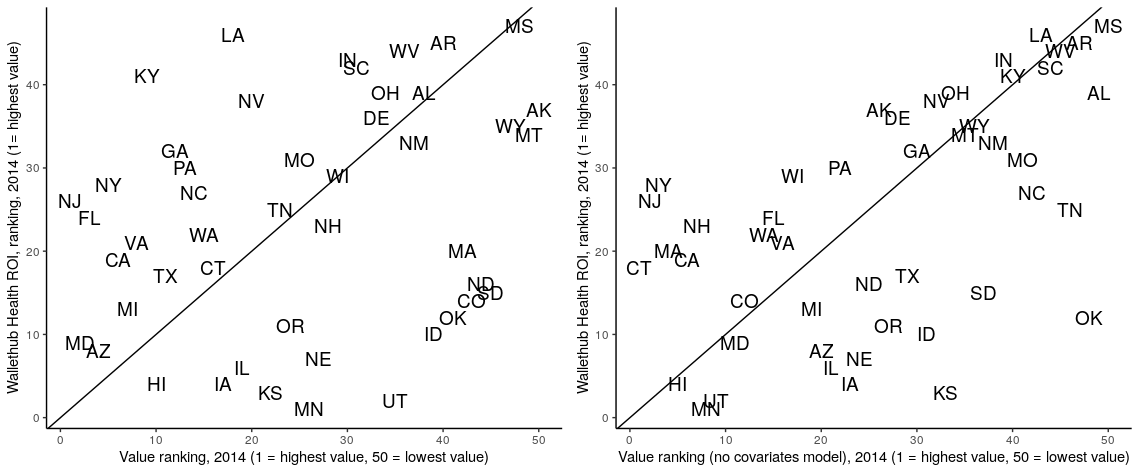


# Section 7. Inefficiency Scores with Alternate Specifications

To assess the robustness of the analysis, we modeled estimates with the following ten alternative specifications (**Figures 7.1 – 7.10**). Scatter plots show ranks of the value score from 2014 with the primary model on the X axis and the sensitivity model on the Y-axis. The grey bars represent 95% confidence intervals of the ranks, calculated by ranking each draw of the estimated value score and taking the 2.5th and 97.5th quantiles.

**Figure 7.1.** **Disability-adjusted life-years instead of deaths:** *The frontier model was run with DALYs per incident case as the outcome variable instead of mortality-incidence ratio.*


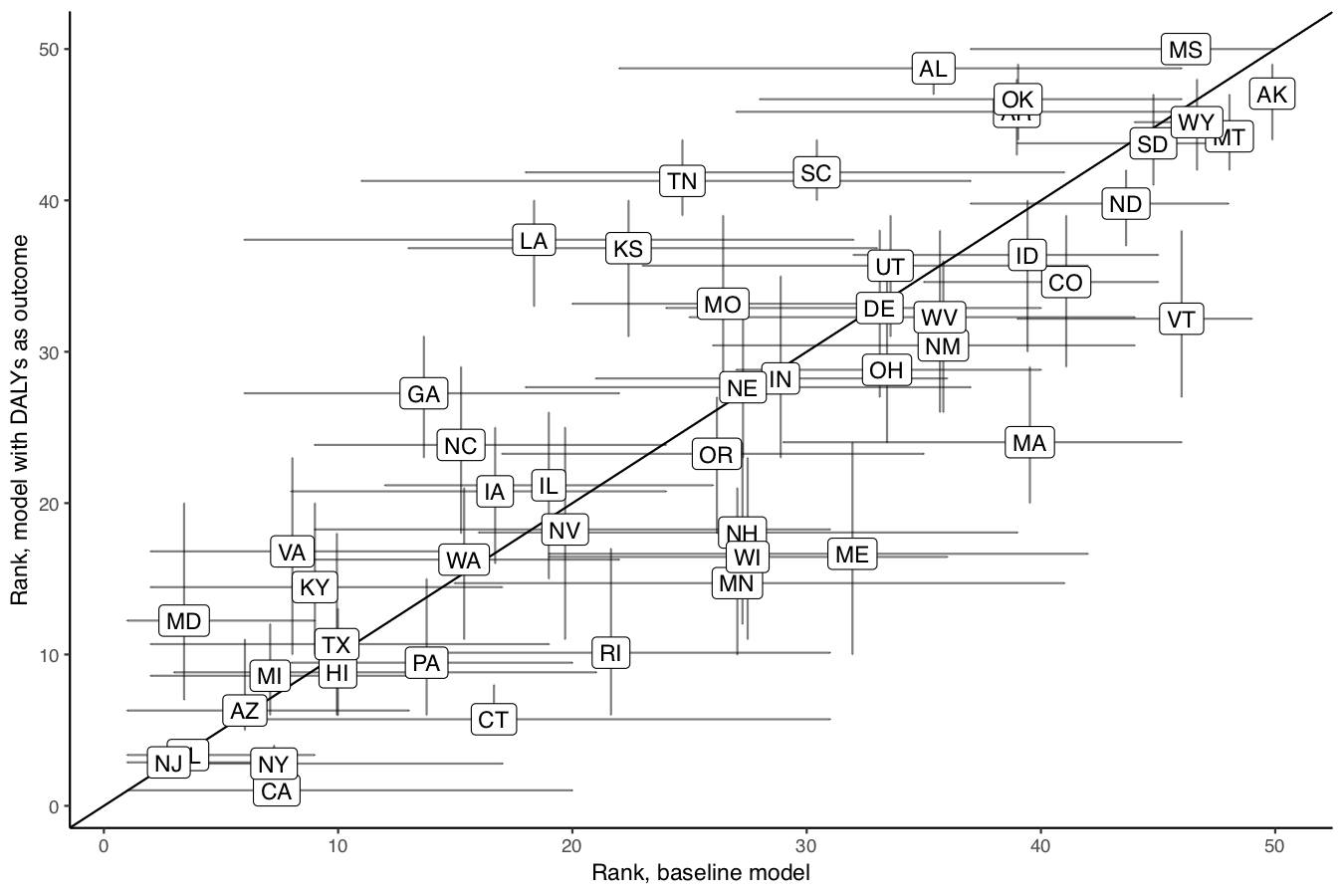


**Figure 7.2. Physician salary-adjusted spending**: *Health care spending was adjusted for physician salaries instead of using BEA implicit price deflation estimates.*


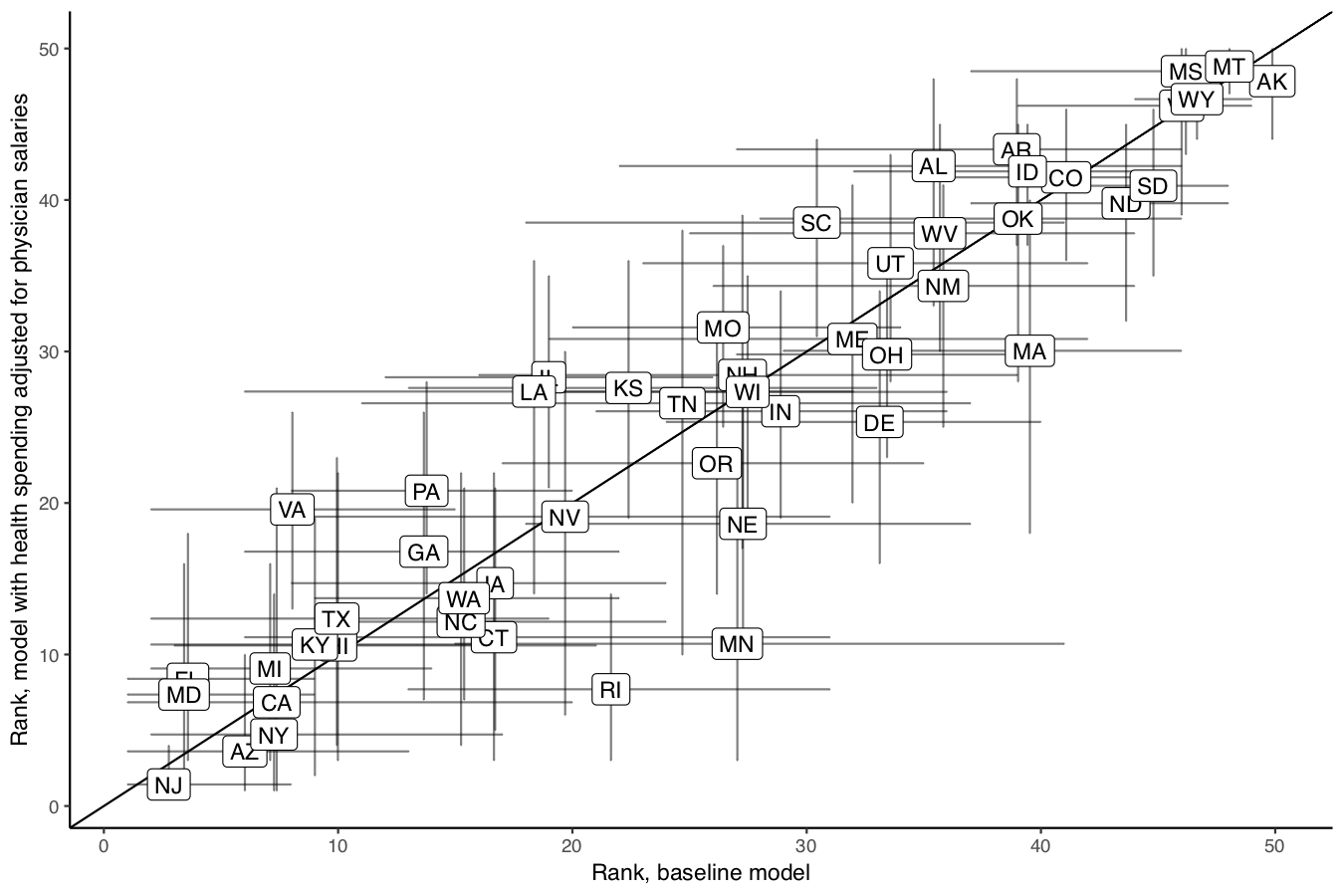


**Figure 7.3. All covariates:** *The model was run with all covariates from the primary model (obesity, cigarettes consumed per capita, proportion of the population older than 65, education, physical activity, and proportion of the population identifying as non-Hispanic white) included by default, rather than running the two-step covariate selection process described above.*


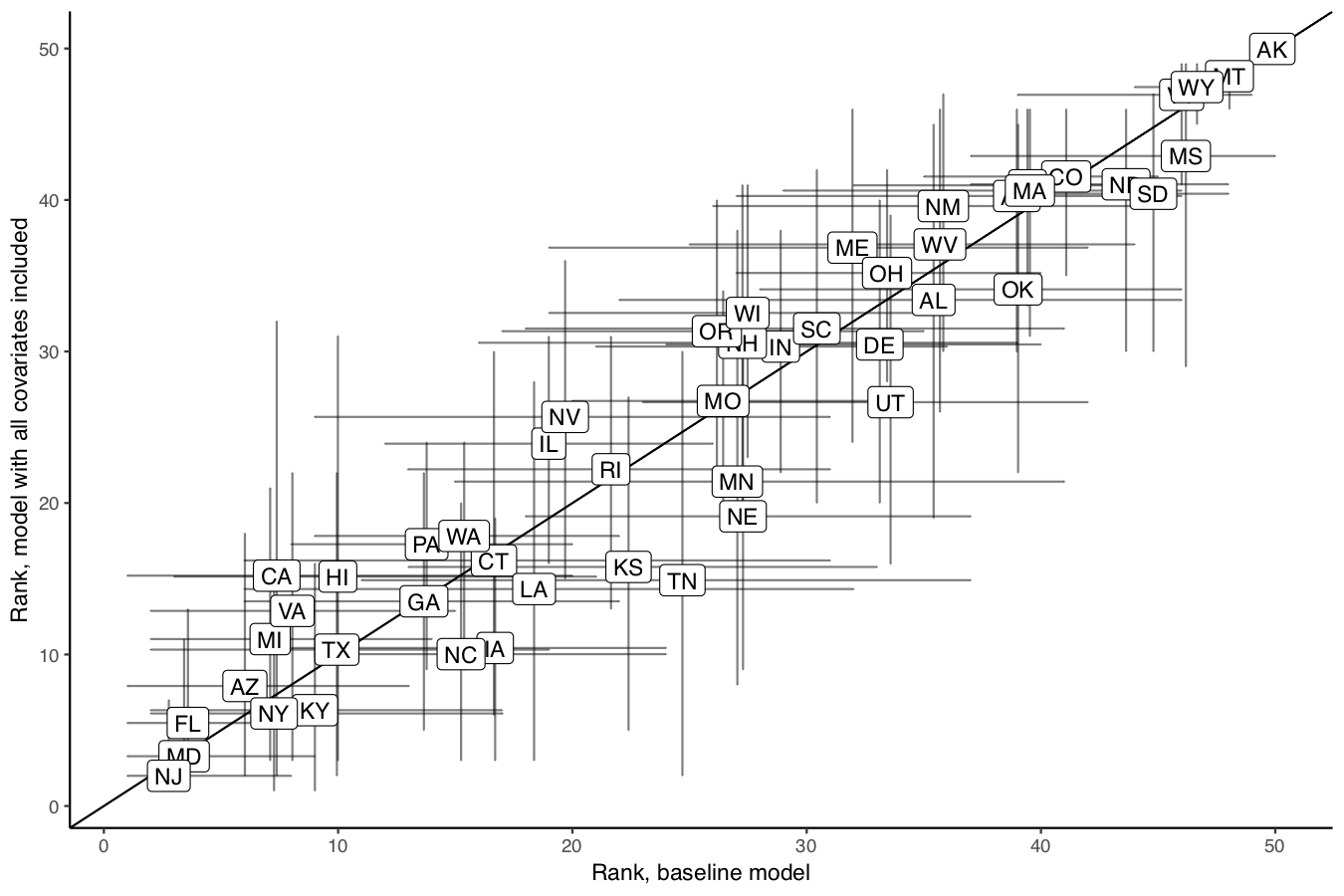


**Figure 7.4. No covariates:** *Frontier model was run with no covariates.*
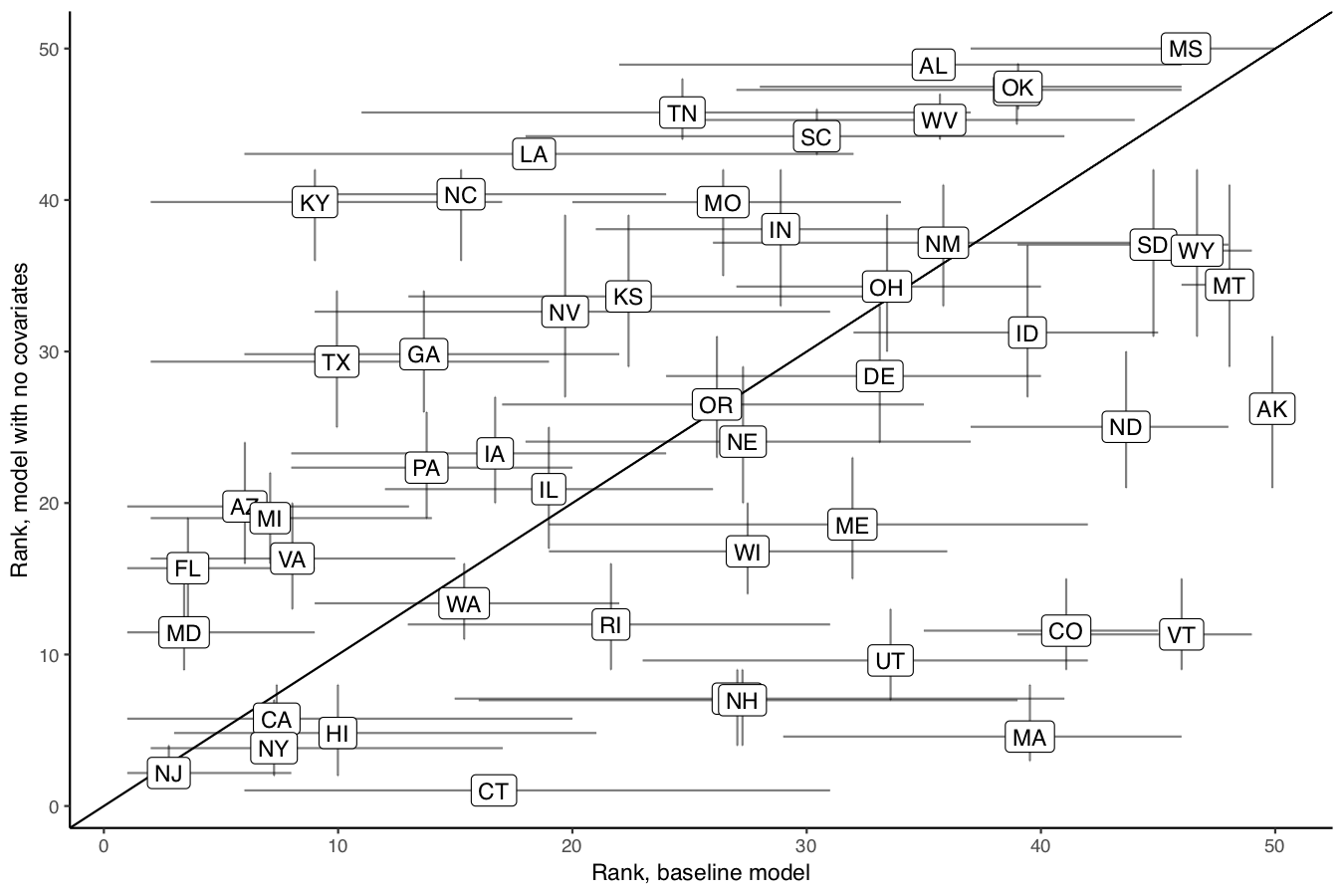


**Figure 7.5. No education:** *The frontier model was run with the standard covariate set and two-step covariate selection process, but without education.*
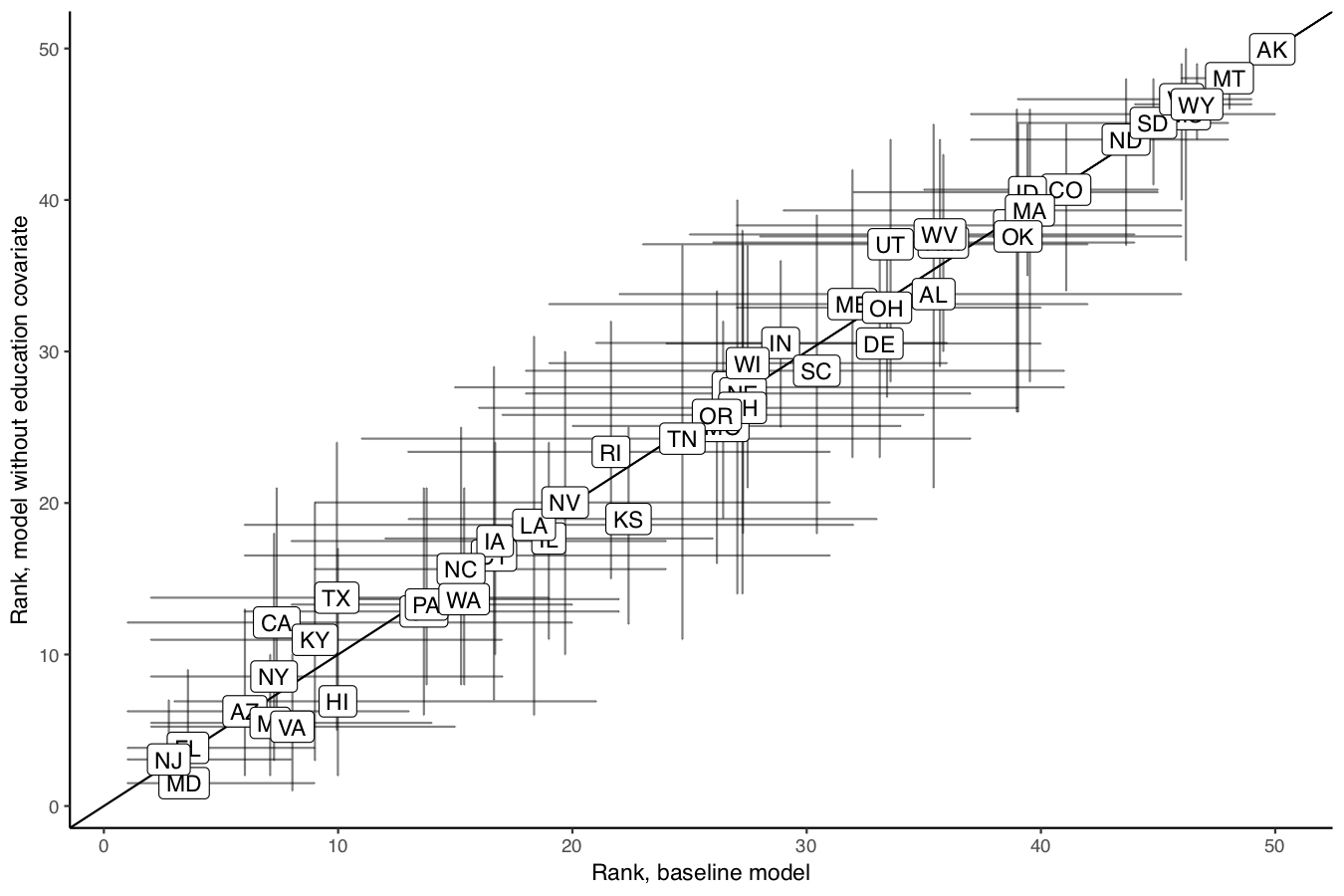


**Figure 7.6. Income instead of education:** *Model run with income covariate in place of education*.
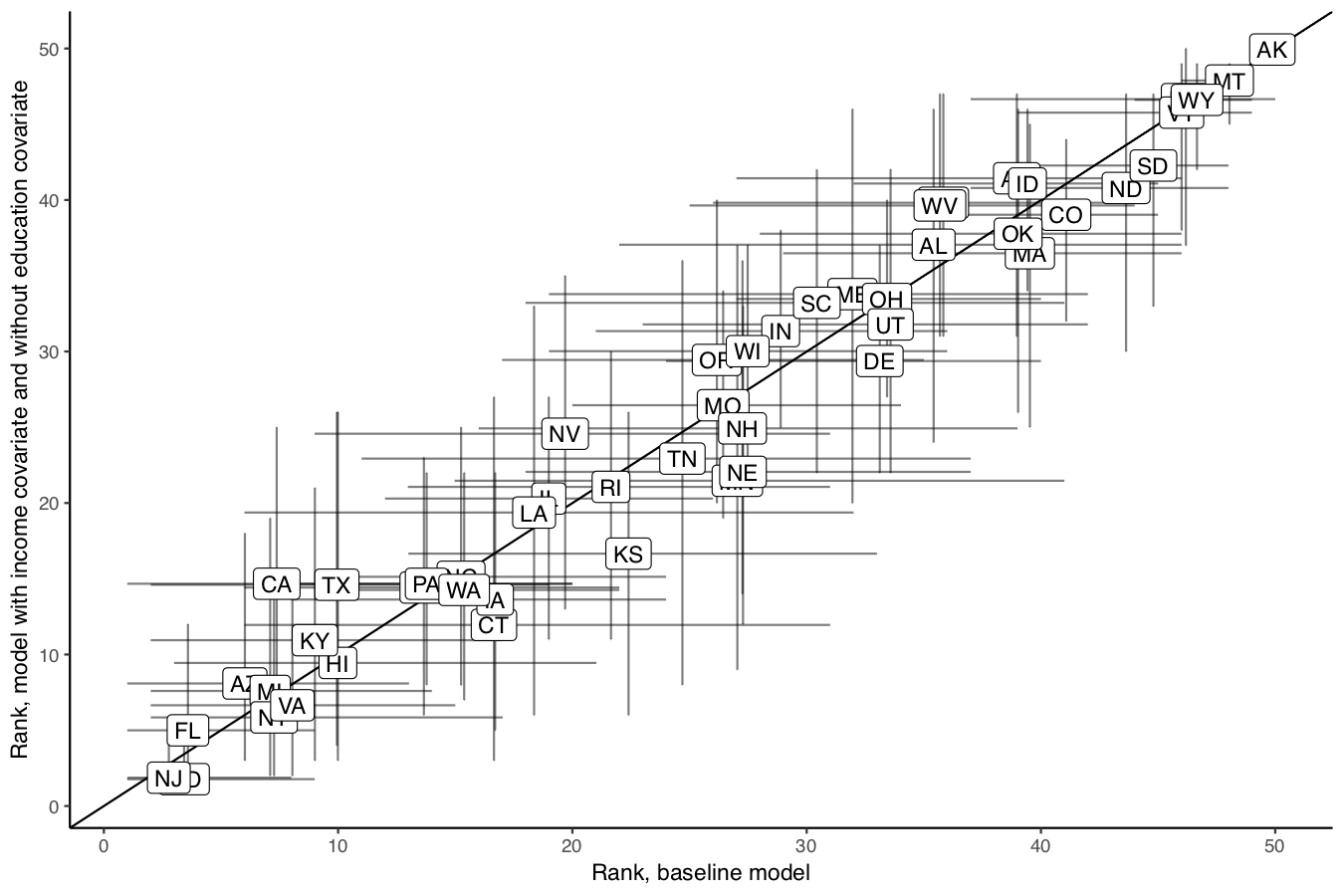


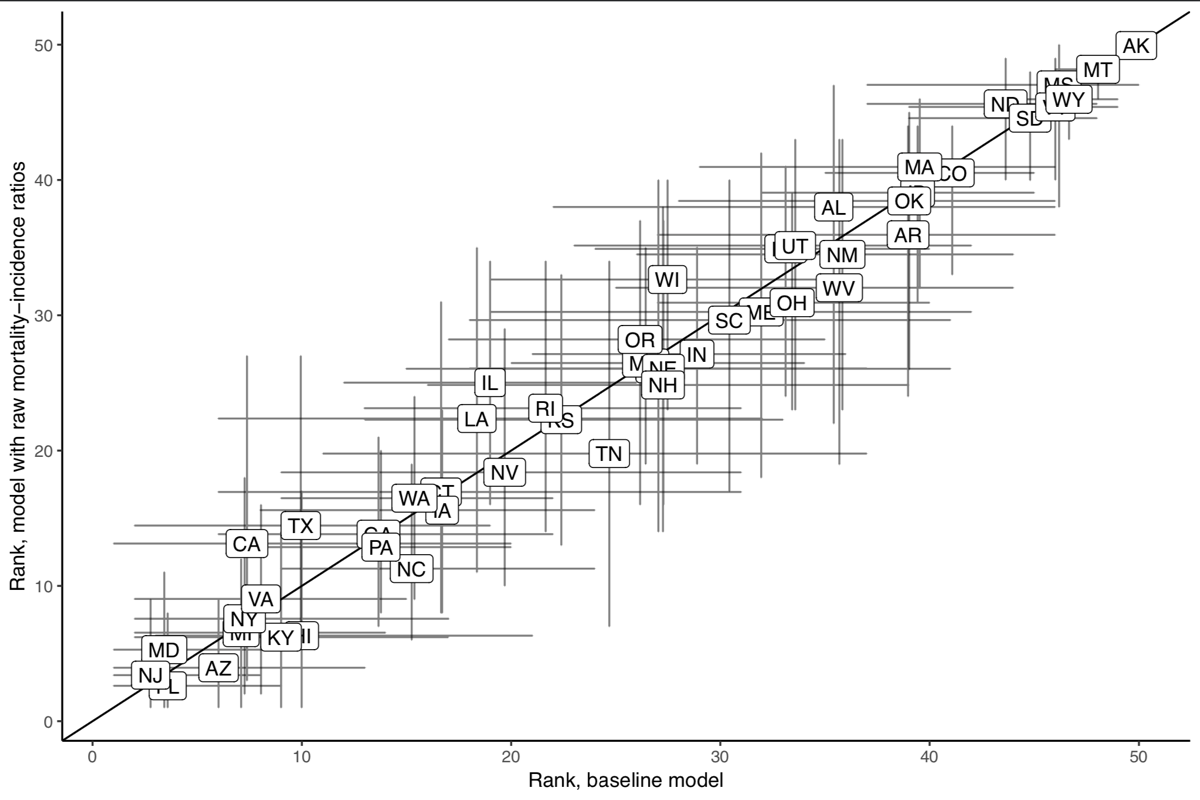
**Figure 7.7. Raw MI ratios:** *Model run without de-meaning mortality-incidence ratios*

**Figure 7.8. Including population density covariate:** *Model run with covariate for population density.*


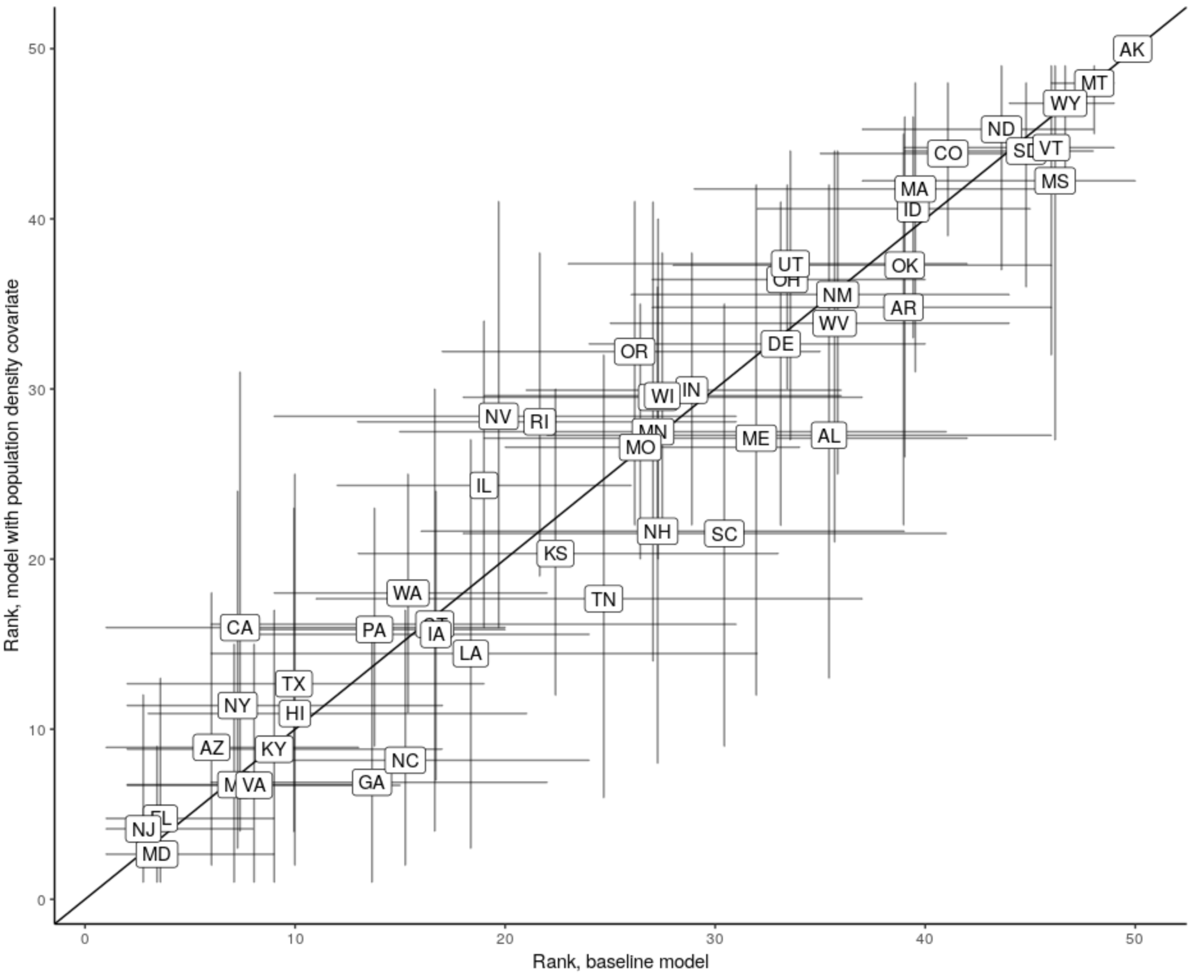


**Figure 7.9. Model with 10% of outliers trimmed:** *To test the sensitivity to outliers, we rerun the analysis using a trimming function with 10% of outliers removed*


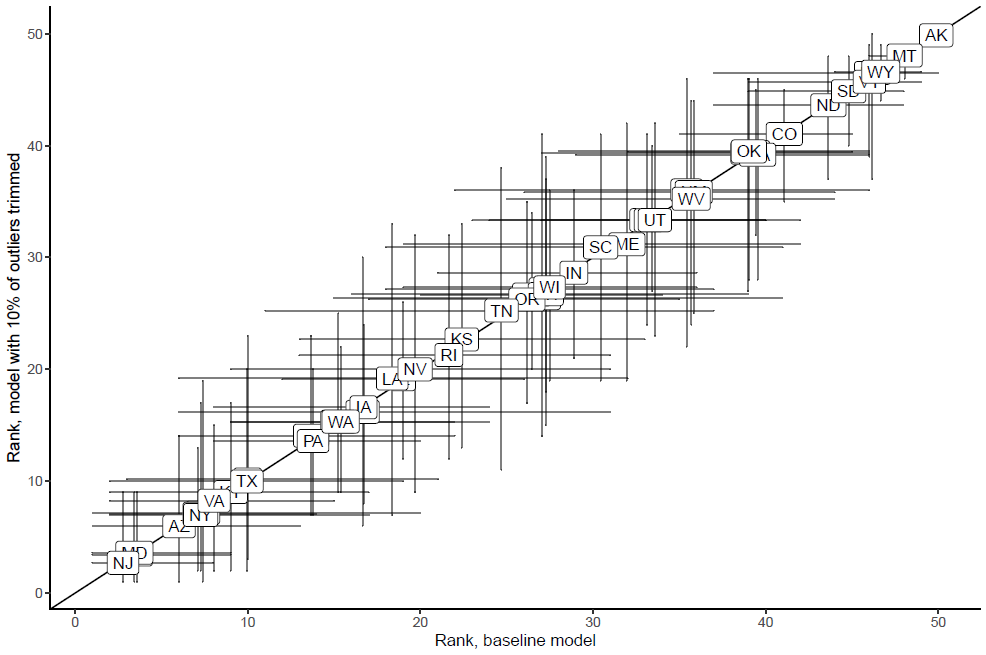


**Figure 7.10. Model using MI ratios for acute conditions and cancers, and MP ratios for other chronic conditions:** *We explore the effect of using Mortality-Prevalence ratios for chronic conditions such as heart disease, COPD, and dementia - instead of Mortality-Incidence ratios. For acute conditions (e.g. infectious diseases and injuries) as well as cancers, we continue to use Mortality-Incidence ratios for this sensitivity analysis.*


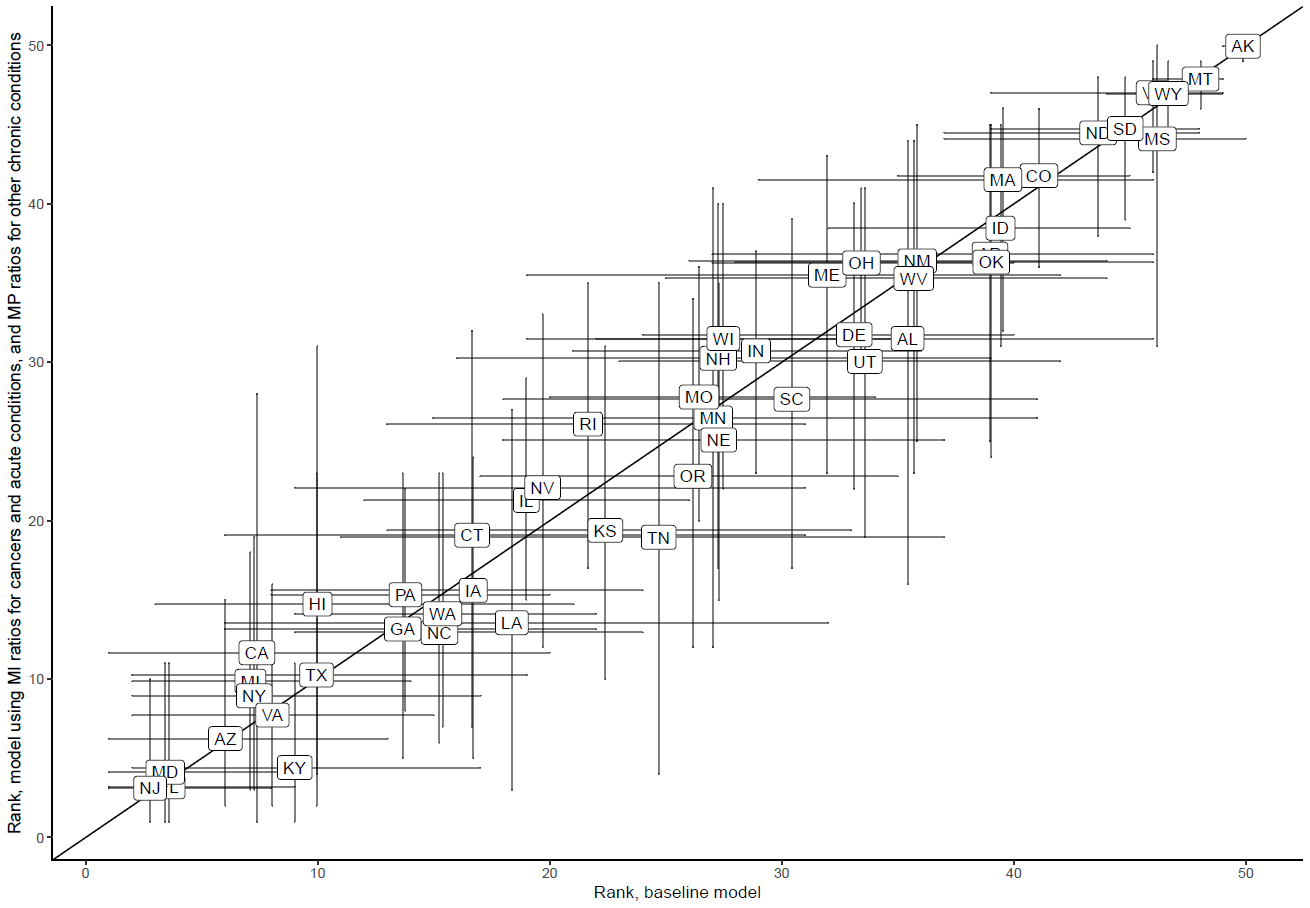


# Section 8. Sensitivity Analysis for Policy Variable Modeling

The policy analysis was additionally assessed for robustness using sensitivity analysis specifications described in **Section 7**, plus one additional analysis removing the state-specific fixed effects. **Figure 8.1** shows the original baseline model, which includes state fixed effects and **Figure 8.2** shows results that remove state fixed effects. The subsequent **Figures** **8.3-8.12** reflect the **Section 7** specifications.

**Figure 8.1. Policy Variable Results, Baseline Model (includes state fixed effects)**


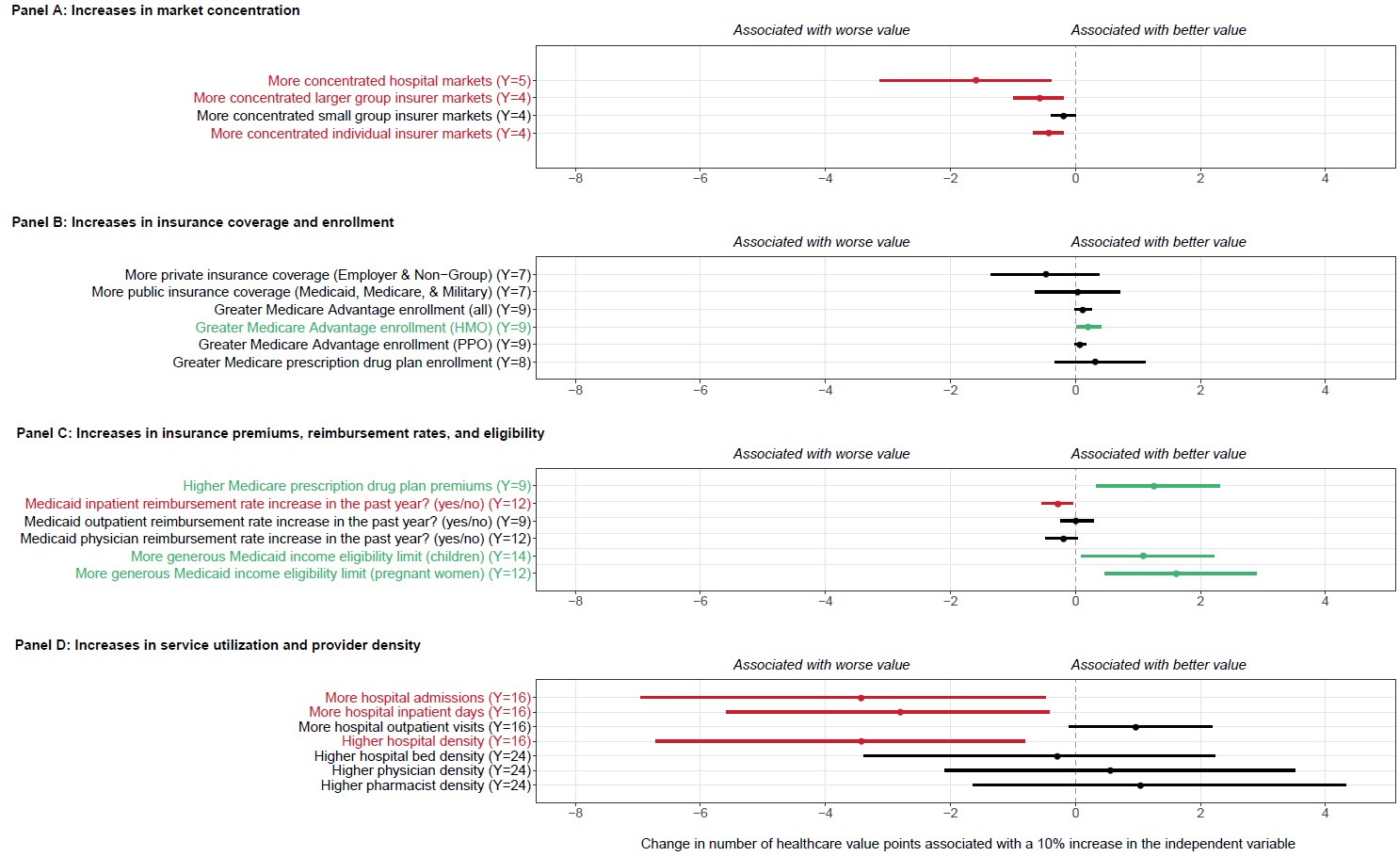


**Figure 8.2. Policy Variable Results, Excluding State Fixed Effects**


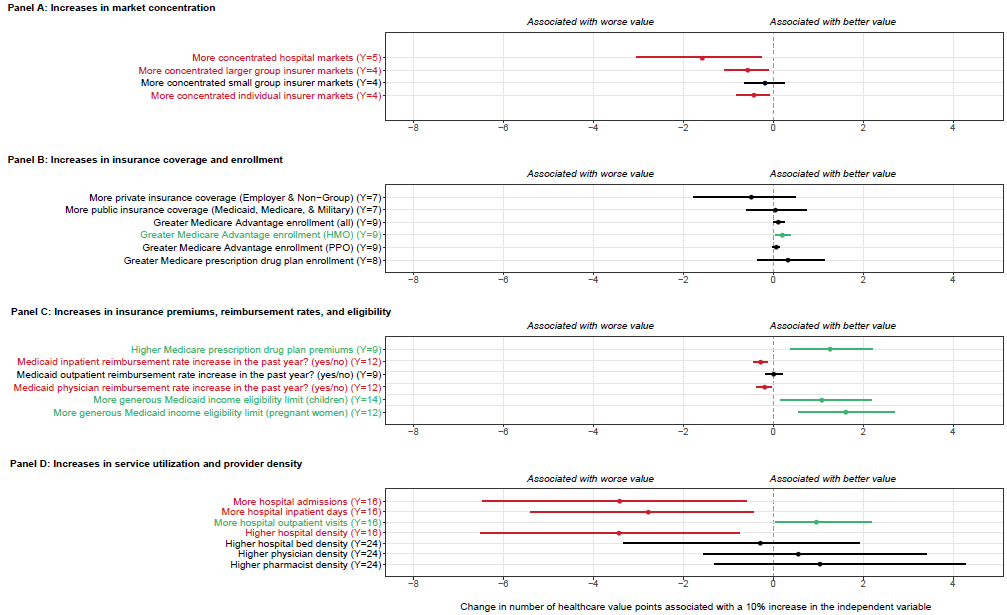


**Figure 8.3. Disability-adjusted life-years instead of deaths**


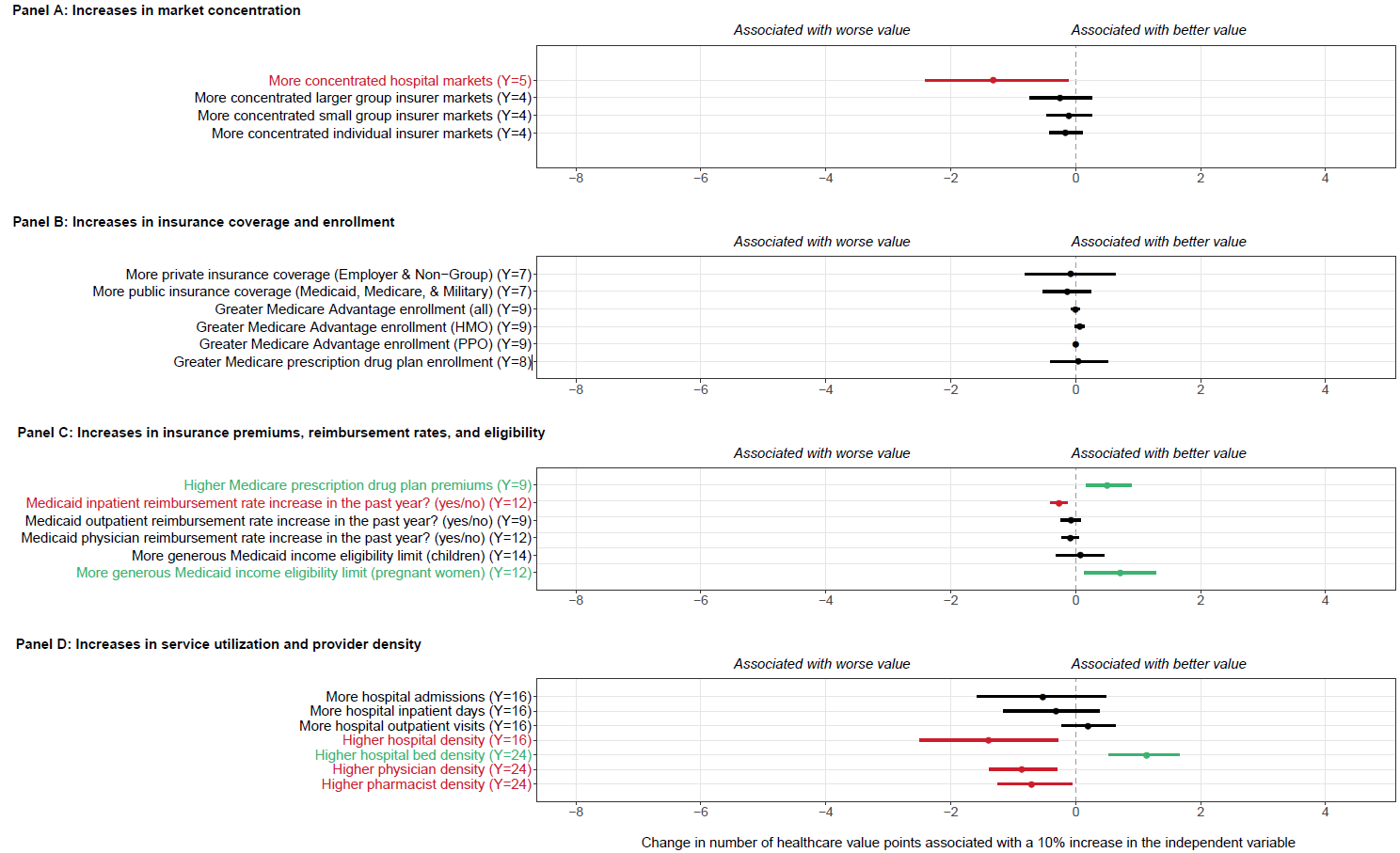


**Figure 8.4. Physician salary-adjusted spending**


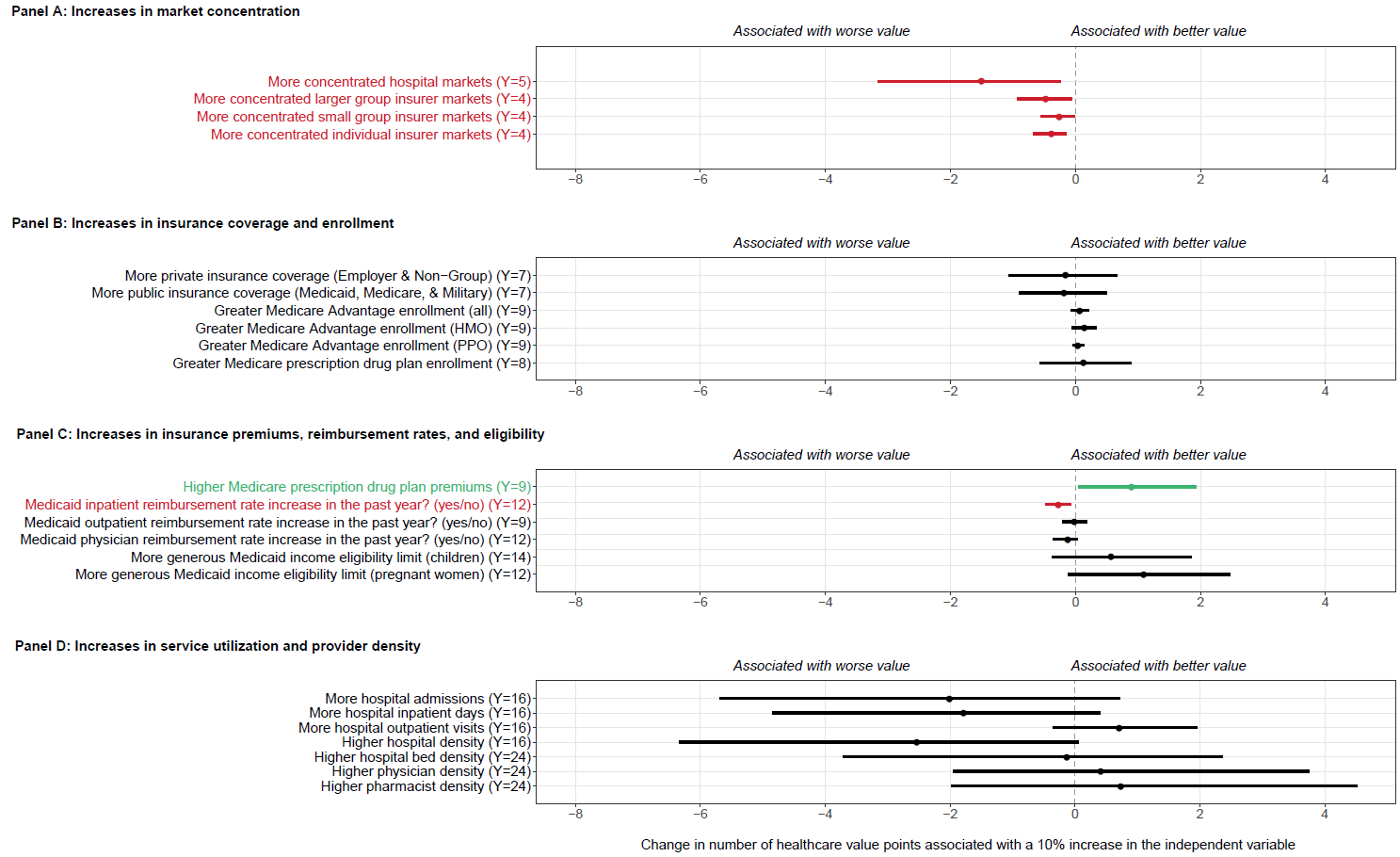


**Figure 8.5. All covariates**


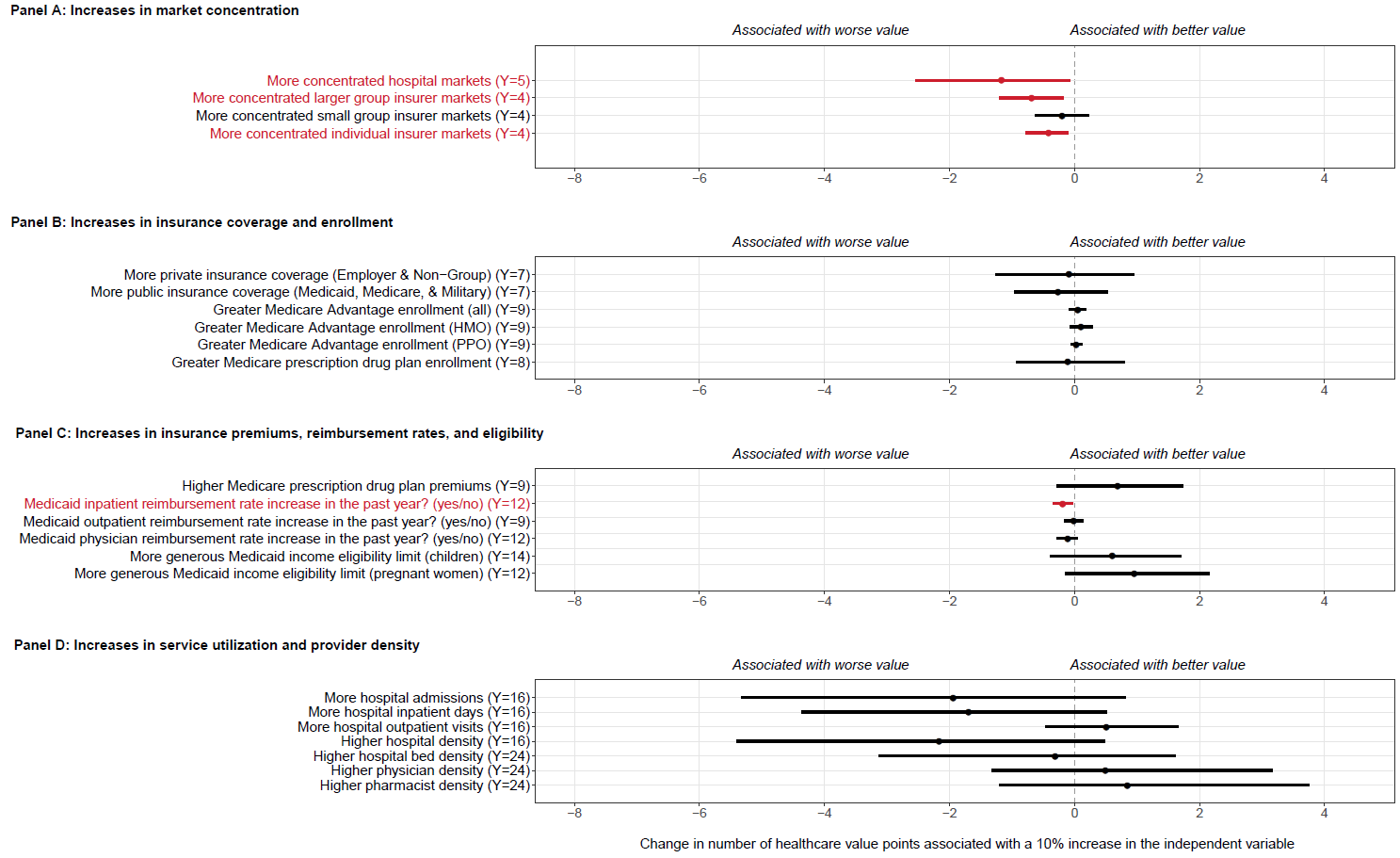


**Figure 8.6. No covariates**


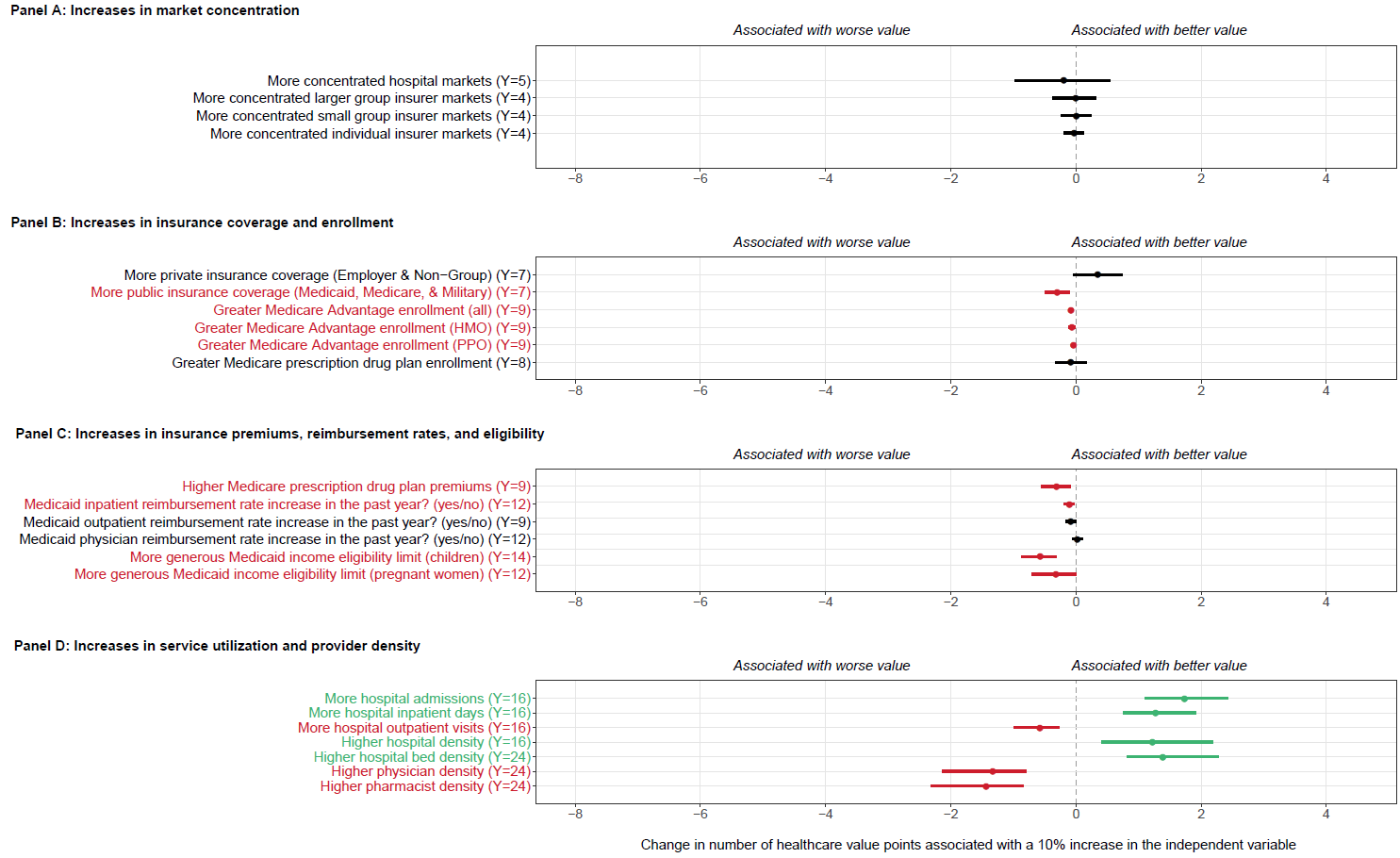


**Figure 8.7. No education**


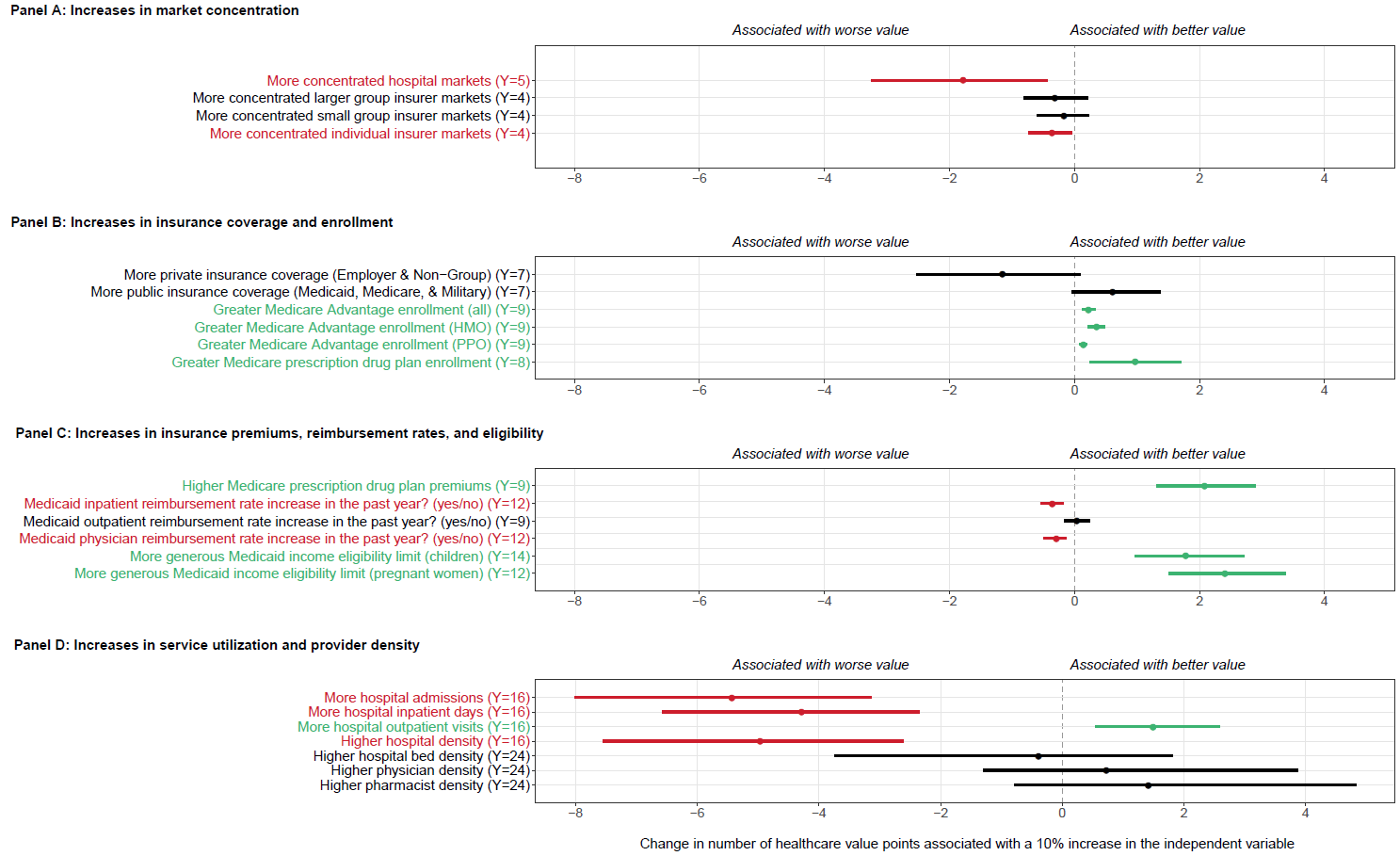


**Figure 8.8. Income instead of education**


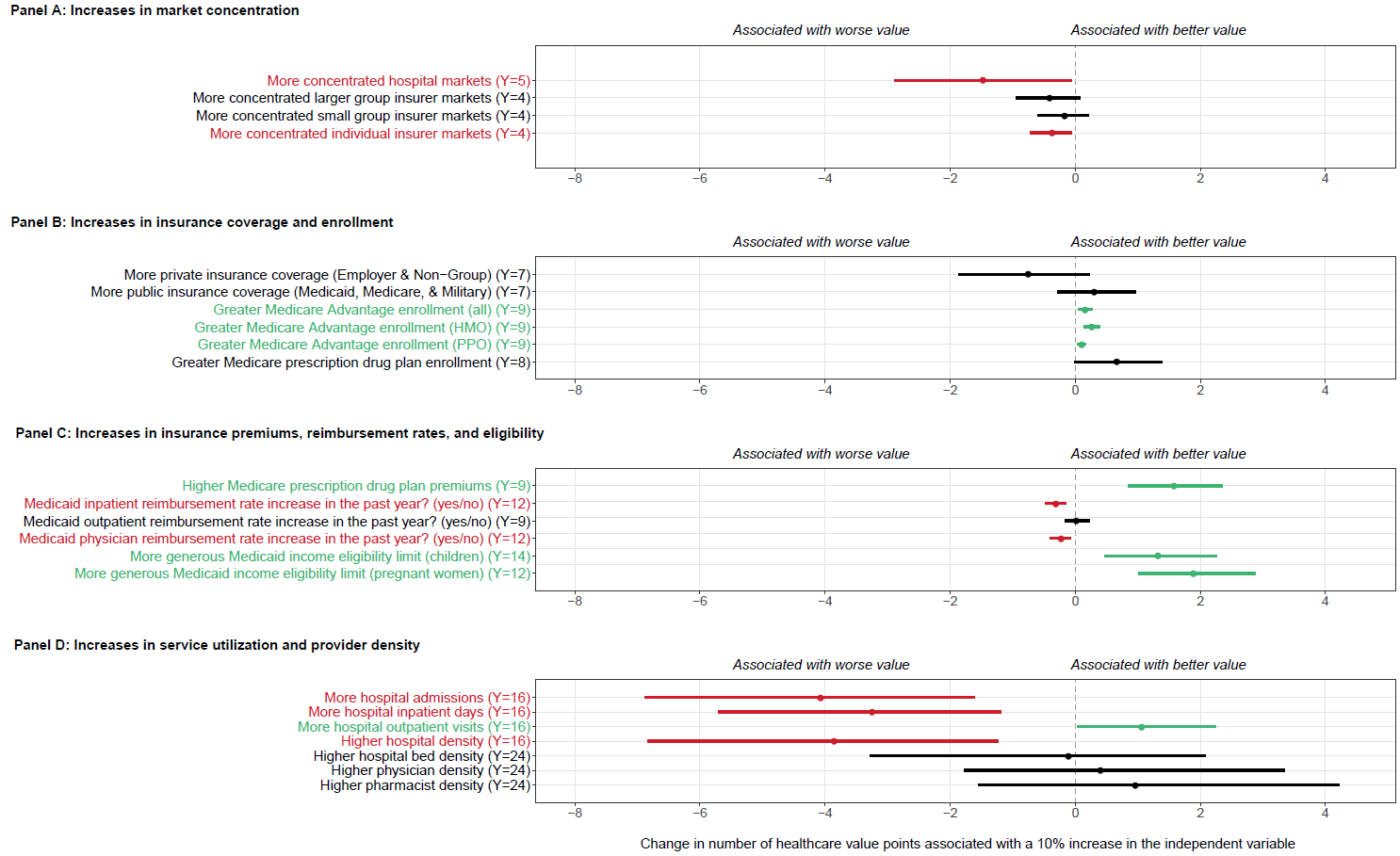


**Figure 8.9. Raw MI ratios (without demeaning)**


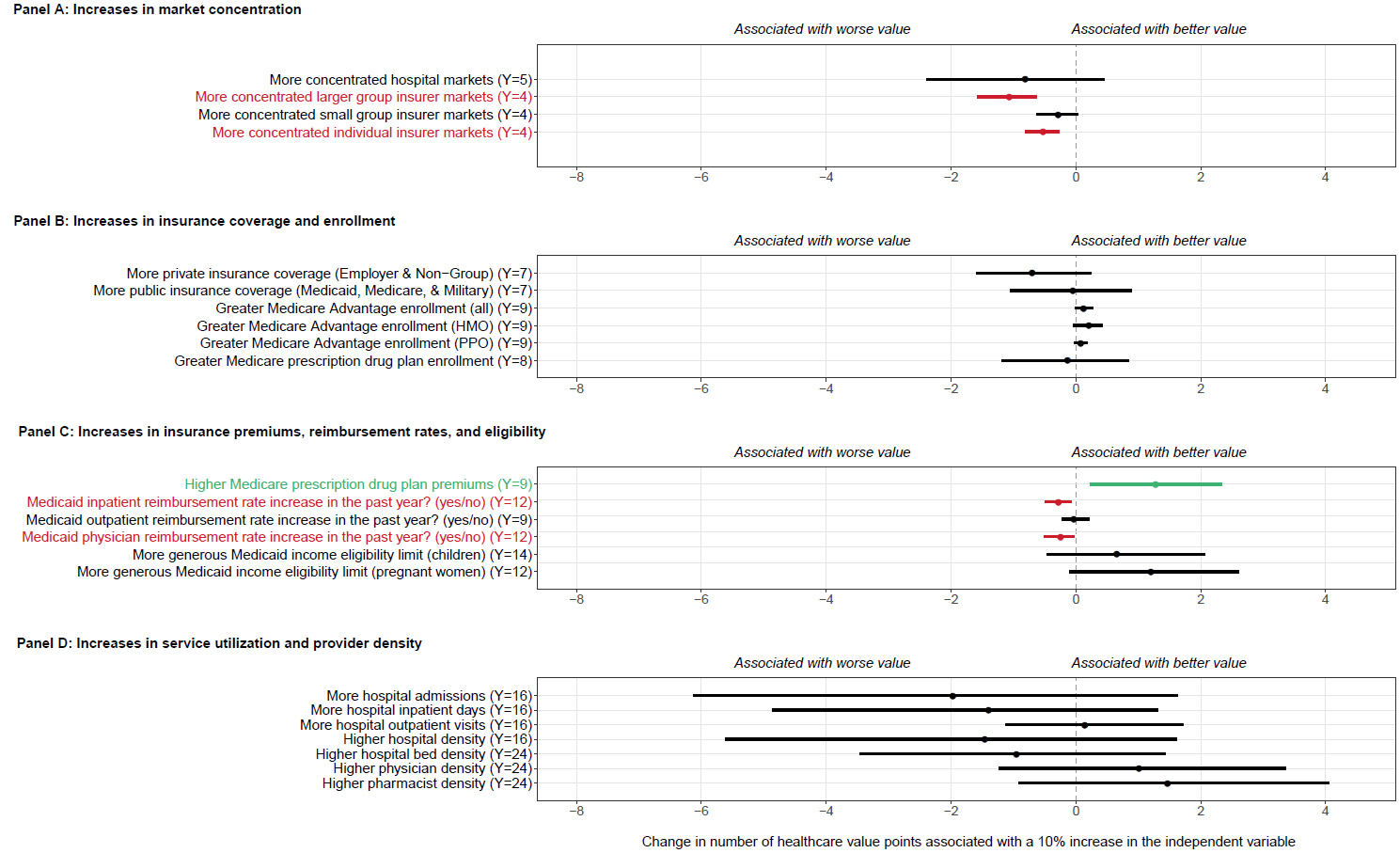


**Figure 8.10 Include population density covariate**


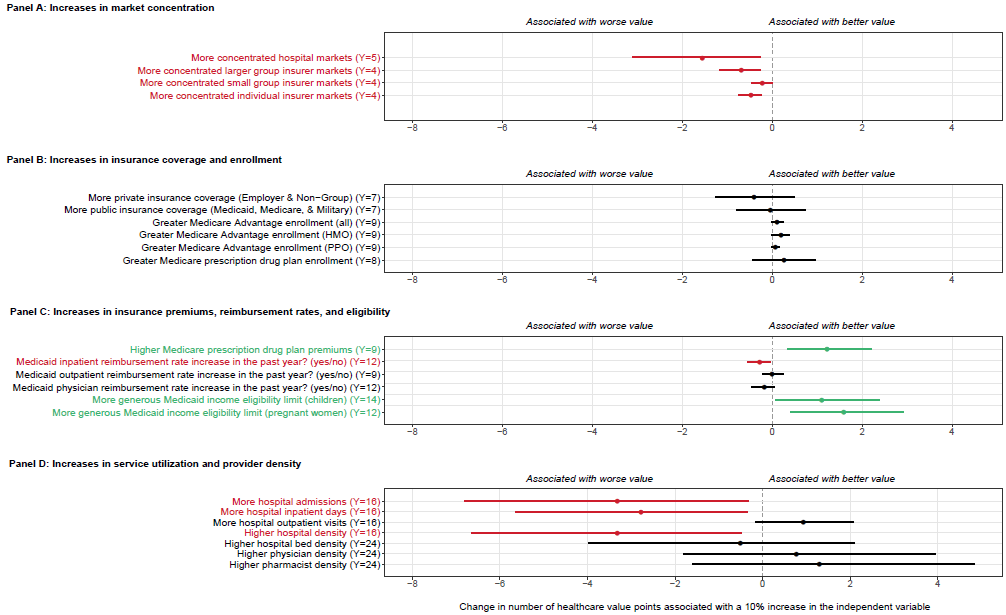


**Figure 8.11 Model with 10% of outliers trimmed**


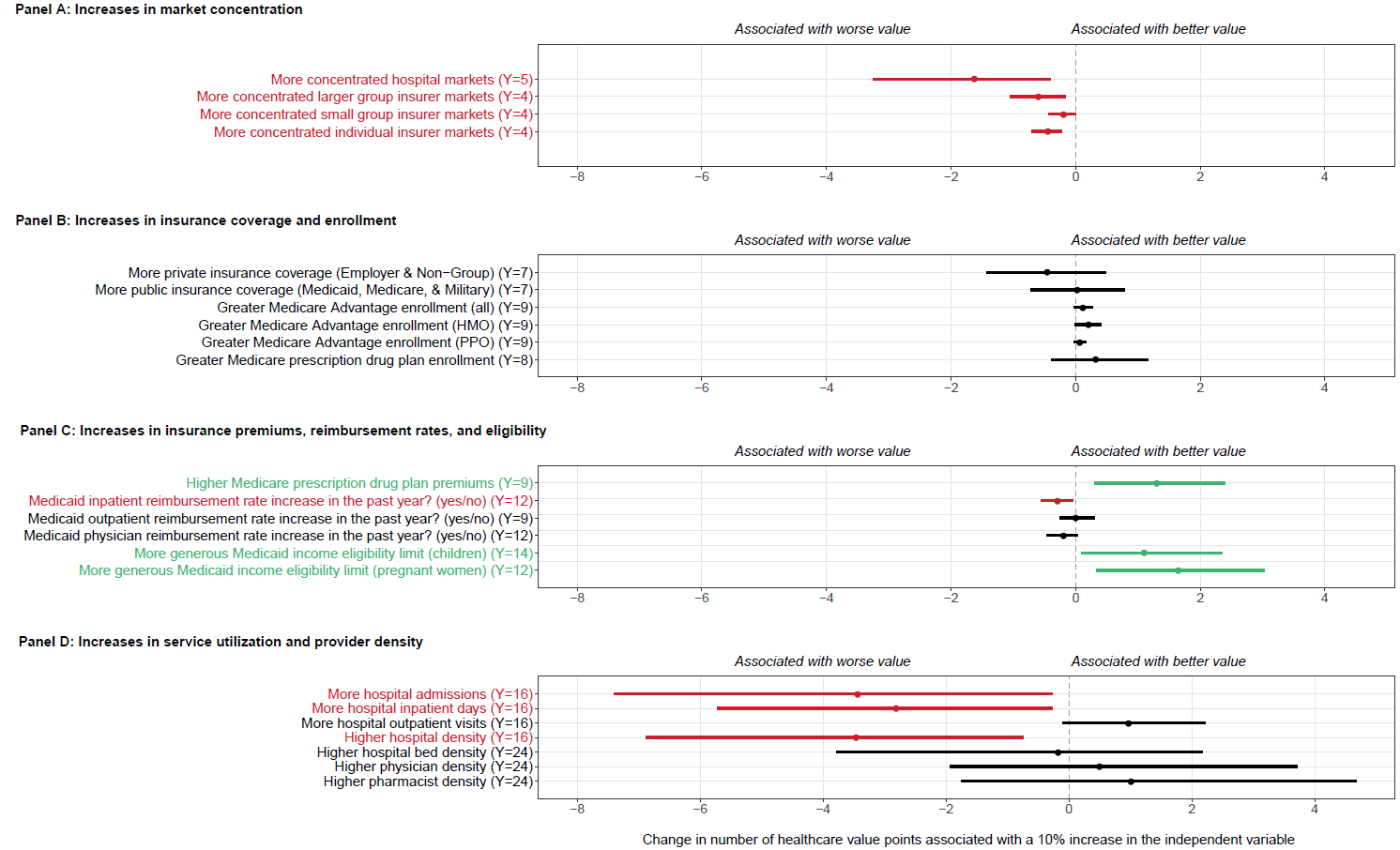


**Figure 8.12 MI ratios for acute conditions and cancers, and MP ratios for other chronic conditions**


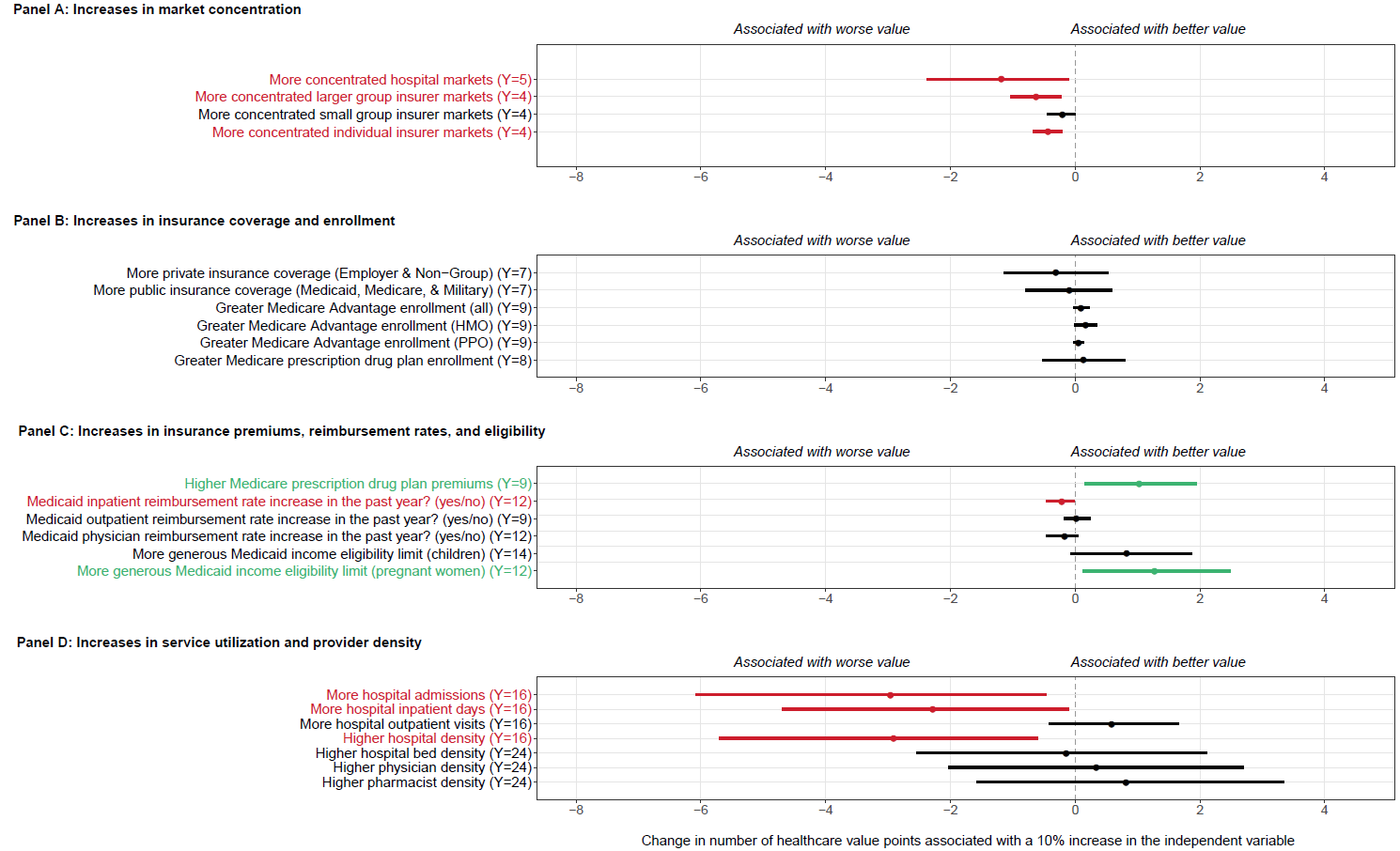


# References

1. Centers for Medicare & Medicaid Services. *Health Expenditures by State of Residence, 1991-2014*. Accessed October 24, 2019. https://www.cms.gov/Research-Statistics-Data-and-Systems/Statistics-Trends-and-Reports/NationalHealthExpendData/NationalHealthAccountsStateHealthAccountsResidence

2. Bureau of Economic Analysis. SAIRPD Implicit Price Deflators by state. Accessed March 20, 2020. https://apps.bea.gov/iTable/iTable.cfm?reqid=70&step=1#reqid=70&step=1

3. U.S. Bureau of Labor Statistics. Occupational Employment Statistics. Healthcare Practitioners and Technical Occupations (Major Group). Accessed April 2, 2020. https://www.bls.gov/oes/2018/may/oes290000.htm

4. Vos T, Allen C, Arora M, et al. Global, regional, and national incidence, prevalence, and years lived with disability for 310 diseases and injuries, 1990–2015: a systematic analysis for the Global Burden of Disease Study 2015. *The Lancet*. 2016;388(10053):1545-1602. doi:10.1016/S0140-6736(16)31678-6

5. United States Census Bureau. US Census Data. Race. https://data.census.gov/cedsci/

6. Healthcare Cost and Utilization Project (HCUP), Agency for Healthcare Research and Quality. Introduction to the HCUP National Inpatient Sample (NIS) 2012. Published online 2012.

7. Kaiser Family Foundation. State Health Facts. Accessed April 18, 2020. https://www.kff.org/statedata/

8. Aigner D, Lovell CAK, Schmidt P. Formulation and estimation of stochastic frontier production function models. *Journal of Econometrics*. 1977;6(1):21-37. doi:10.1016/0304-4076(77)90052-5

9. De Boor C. A Practical Guide to Splines, volume 27 of. *Applied mathematical sciences*. Published online 1978:15-16.

10. Friedman JH. Multivariate adaptive regression splines. *The annals of statistics*. Published online 1991:1-67.

11. Pya N, Wood SN. Shape constrained additive models. *Statistics and computing*. 2015;25(3):543-559.

12. Bell BM, Burke JV, Schumitzky A. A relative weighting method for estimating parameters and variances in multiple data sets. *Computational Statistics & Data Analysis*. 1996;22(2):119-135. doi:10.1016/0167-9473(95)00043-7

13. Golub G, Pereyra V. Separable nonlinear least squares: the variable projection method and its applications. *Inverse problems*. 2003;19(2):R1.

14. Aravkin AY, van Leeuwen T. Estimating nuisance parameters in inverse problems. *Inverse Problems*. 2012;28(11):115016. doi:10.1088/0266-5611/28/11/115016

15. Wächter A, Biegler LT. On the implementation of an interior-point filter line-search algorithm for large-scale nonlinear programming. *Mathematical programming*. 2006;106(1):25-57.

16. Aravkin A, Davis D. Trimmed Statistical Estimation via Variance Reduction. *Mathematics of OR*. 2019;45(1):292-322. doi:10.1287/moor.2019.0992

17. Altarum Healthcare Value Hub. *Healthcare Affordability State Policy Scorecard: Summary Report.*; 2020. https://www.healthcarevaluehub.org/application/files/5715/8162/2389/Healthcare_Affordability_Scorecard_-_Summary_Report.pdf

18. McCarthy DM, Radley DCR, Hayes SLH. *2018 Scorecard on State Health System Performance*. Commonwealth Fund; 2018. doi:10.15868/socialsector.30564

19. U.S. News & World Report. Health care rankings: Measuring how well states are meeting citizens’ health care needs. Published 2020. Accessed May 15, 2020. https://www.usnews.com/news/best-states/rankings/health-care

20. Barrington R. Vital Signs: Best States for Healthcare 2020. MoneyRates. Published March 5, 2020. Accessed May 15, 2020. https://www.money-rates.com/research-center/compare-healthcare-by-state.htm

21. United Health Foundation. America’s Health Rankings. Public Health Impact: Overall. Published 2020. Accessed May 15, 2020. https://www.americashealthrankings.org/explore/annual/

22. Bernardo R. 2014’s States with the Best & Worst Health ROI. WalletHub. Published July 30, 2014. Accessed May 20, 2020. https://wallethub.com/edu/states-with-the-best-worst-health-roi/5247/#methodology
